# Cross‐Modal Denoising and Integration of Spatial Multi‐Omics Data with CANDIES

**DOI:** 10.1002/advs.202523754

**Published:** 2026-04-27

**Authors:** Ye Liu, Wanpeng Zou, Yuekai Li, Jiayi Wang, Mingxuan Cai, Hongmin Cai

**Affiliations:** ^1^ School of Future Technology South China University of Technology Guangzhou Guangdong China; ^2^ Department of Biostatistics City University of Hong Kong Hong Kong China; ^3^ School of Computer Science and Engineering South China University of Technology Guangzhou Guangdong China

**Keywords:** complex traits, diffusion model, multi‐omics integration, spatial transcriptomics

## Abstract

Spatial multi‐omics data offer a powerful framework for integrating diverse molecular profiles while maintaining the spatial organization of cells. However, inherent variations in data quality and noise levels across different modalities pose significant challenges to accurate integration and analyses. In this paper, we introduce CANDIES, which leverages a conditional diffusion model and contrastive learning to effectively denoise and integrate spatial multi‐omics data. With our innovative model and algorithm designs, CANDIES not only enhances the quality of spatial multi‐omics data, but also yields a unified and comprehensive joint representation, thereby empowering many downstream analyses. We conduct extensive evaluations on diverse synthetic and real datasets, including MISAR‐seq data from the mouse brain, spatial CITE‐seq data from human skin biopsy tissue, spatial Mux‐seq, and spatial ATAC‐RNA‐seq data from the mouse embryo, and 10× Visium data from human lymph nodes. CANDIES shows superior performance on various downstream tasks, including denoising, spatial domain identification, spatiotemporal trajectory reconstruction, and spatial association mapping for complex human traits. In particular, we show that CANDIES representations can be integrated with the rich resources from genome‐wide association studies (GWASs), allowing the spatial domains to be linked with complex human traits, yielding spatially resolved interpretations of complex traits in their relevant tissues.

## Graphical abstract

In this paper, we introduce CANDIES, which leverages a conditional diffusion model and contrastive learning to effectively denoise and integrate spatial multi‐omics data. We conduct extensive evaluations on diverse synthetic and real datasets, CANDIES shows superior performance on various downstream tasks, including denoising, spatial domain identification, spatiotemporal trajectory reconstruction, and spatial association mapping for complex human traits.

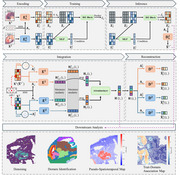

## 1 Introduction

Groundbreaking spatially resolved omics technologies have become powerful tools for understanding cellular states and cellular organization within tissue. Recent advancements in spatial multi‐omics techniques have enabled the simultaneous spatial measurement of multiple molecular profiles within the same tissue section [1]. For example, co‐detection methods for protein and gene expression include DBiT‐seq [2], Spatial CITE‐seq [3], Stereo‐CITE‐seq [4], and CosMxTM Spatial Molecular Imager (SMI) [5]. Additionally, the comapping of the chromatin accessibility and gene expression is achieved by spatial assay for transposase‐accessible chromatin and RNA using sequencing (ATAC–RNA‐seq) [6] and MISAR‐seq [7]. This development allows for a more comprehensive understanding of the molecular landscape within tissues, offering new insights into tissue organization, function, and disease mechanisms [8].

Despite advancements in spatial multi‐omics technologies, analyzing spatial multi‐omics data remains highly challenging. One major hurdle is the inherent noise within each modality, which can obscure meaningful biological signals and hinder accurate data integration. For instance, technical noise introduced during spatial transcriptomic sequencing, coupled with false zero counts in gene expression measurements due to low RNA capture efficiency, poses major obstacles to accurate analysis and downstream applications [9, 10, 11]. Second, extracting data from one modality might disrupt the molecular stability required for another, which could diminish the accuracy of subsequent molecular sequencing and lead to inconsistencies in data quality across different modalities [12]. For example, due to suboptimal antibody‐derived tags (ADTs) concentrations and enzymatic reaction parameters, competition between ADTs and mRNAs during reverse transcription can reduce transcript detection efficiency compared to single‐modality spatial transcriptomics [3]. Third, each modality provides unique insights into cellular identity, offering complementary yet distinct advantages and limitations. The integration of multi‐omics data is needed to leverage these diverse perspectives to improve the precision and sensitivity of identifying cell types and states, ultimately enabling a more comprehensive understanding of cellular heterogeneity and function [13].

Several methods, such as MultiVI [14], totalVI [15], and scCross [16] have been developed to integrate single‐cell multi‐omics data through deep generative models. For instance, scCross employed a bidirectional aligner and a discriminator for high‐quality modality generation, allowing researchers to derive data from one modality based on another. However, biological molecular regulatory relationships are not solely dependent on molecular characteristics, they are also influenced by the tissue microenvironment [17]. Recently, many computational algorithms, including SpaGCN [18], GraphST [19], SEDR [20], and Giotto [21], have been developed for spatially resolved data. But these tools are designed for specific data modalities, such as spatial transcriptomics (ST) [22, 23] or spatial proteomics [24], often providing a fragmented view of the cellular landscape. Although these methods can be extended to spatial multi‐omics data by concatenating features from various modalities, they remain suboptimal due to the inherent heterogeneity across these modalities. The field of spatial multi‐omics integration is still in its early stages, with several recent methods such as SpatialGlue [25], PRESENT [26], COSMOS [27] and PRAGA [28], attempting to address the challenges of cross‐modality representation with spatial context. However, these methods are often sensitive to noise, which can degrade their performance and limit their ability to uncover biologically meaningful insights.

To overcome these limitations, we proposed CANDIES, a novel framework leveraging Conditional diffusion model And coNtrastive learning, specifically designed for cross‐modal Denoising and IntEgration of Spatial multi‐omics data. By taking spatial multi‐omics data with modalities exhibiting varying levels of noise as input, CANDIES refines the lower‐quality modality by leveraging information from the higher‐quality modality and spatial context. It then integrates spatial and enhanced feature information across modalities to produce robust, unified representations of spatial multi‐omics data. CANDIES significantly improves the accuracy and reliability of downstream analysis, including denoising, spatial domain identification, pseudo‐Spatiotemporal Map (pSM) construction, and spatially resolved mapping of complex human traits. To validate its effectiveness, we first benchmarked CANDIES on a simulated dataset and spatial transcriptome‐epigenome data from mouse brain, where it outperformed state‐of‐the‐art methods in both spatial single‐modality denoising and multi‐modal integration across a wide range of noise levels. Further analysis of spatial transcriptome–protein data from human skin biopsy tissue revealed new biological insights enabled by the denoising module, where CANDIES detected a previously unrecognized cluster corresponding to an immune‐responsive niche within the pilosebaceous unit of human skin. In addition, experiments on spatial H3K27ac–H3K27me3 and spatial epigenome–transcriptome data from mouse embryos demonstrated that, by leveraging multi‐modal integration, CANDIES not only accurately distinguished major biological domains but also preserved the integrity of rare cell types that were undetectable in single‐modality analyses. Lastly, but importantly, CANDIES output can be integrated with summary statistics of genome‐wide association studies (GWASs) to identify spatially resolved tissue domains associated with complex human traits. We integrated CANDIES results from spatial epigenome‐transcriptome data from mouse brain and embryo, as well as spatial transcriptome‐protein data from human lymph nodes, with GWASs from 32 complex traits, uncovering interpretable associations between traits and relevant tissue regions. These results highlight CANDIES' superior capability to extract biologically meaningful insights, providing a powerful framework for advancing the understanding of spatially resolved molecular landscapes.

## 2 Results

### 2.1 CANDIES Model Overview

**FIGURE 1:**
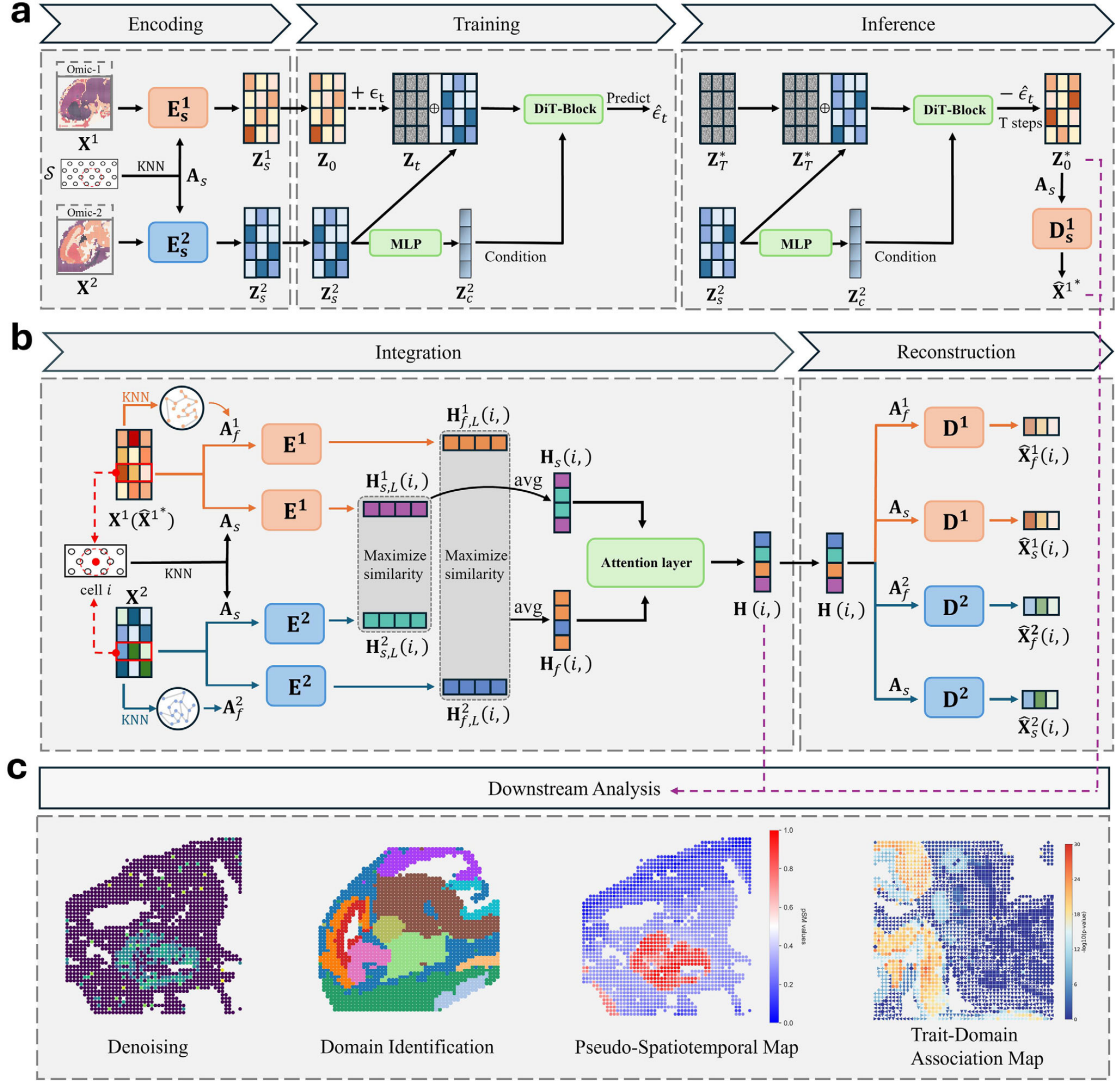
The framework of CANDIES model. (a) The denoising phase of CANDIES. CANDIES begins by constructing a spatial graph using the k‐nearest‐neighbor (KNN) algorithm based on spatial coordinates. A graph auto‐encoder (GAE) is then applied to this spatial graph with the features of each modality to generate low‐dimensional embeddings for each modality, which facilitate the efficient training of the denoising model. Next, a diffusion transformer (DiT)‐based conditional diffusion model enhances the embeddings of the lower‐quality modality by leveraging its spatial dependencies with the higher‐quality modality embeddings. Finally, the denoised embeddings of the lower‐quality modality are decoded back into the original data space. (b) The integration phase of CANDIES. CANDIES encodes denoised spatial multi‐omics data into latent space by applying a dual graph convolution network (GCN) on both spatial proximity and feature similarity graphs for each omic modality. Subsequently, CANDIES aligns cross‐modality embeddings via contrastive learning, and dynamically integrates spatial graph and feature graph through the attention mechanism, effectively preserving cellular heterogeneity while maintaining an optimal balance between spatial organization and molecular feature information. (c) CANDIES supports four downstream analysis including denoising, spatial domain identification, pseudo‐Spatiotemporal Map (pSM), and spatially resolved mapping of complex human traits.

CANDIES, shown in Figure 1, is a comprehensive and innovative framework specifically designed for spatial multi‐omics data with modalities exhibiting different levels of noise. As a two‐stage method, CANDIES first refines the lower‐quality modality by leveraging its spatially resolved dependence on the higher‐quality modality. Subsequently, CANDIES facilitates more precise and robust integration of enhanced spatial multi‐omics data by cross‐modal alignment and multi‐relation fusion, enabling a unified and biologically meaningful representation. By combining these stages, CANDIES effectively mitigates noise and improves the overall effectiveness of spatial multi‐omics data analysis.

In the first stage (Figure 1a), CANDIES enhances the lower‐quality modality data by a diffusion transformer (DiT)‐based conditional diffusion model. Specifically, CANDIES first constructs a spatial graph based on the shared spatial coordinates, then a dual Graph Auto‐Encoder (GAE) is trained to extract complementary information from the spatial graph with features of multiple modalities, generating embeddings for all spots within each modality. In the proposed DiT‐based conditional diffusion model, the embeddings of the lower‐quality modality are initialized as the starting point of the diffusion process, while the embeddings of the higher‐quality modality serve as conditions to guide the reverse process. During the reverse denoising process, the embeddings of the lower‐quality modality and the higher‐quality modality are concatenated and fed into a DiT architecture [29], which acts as the backbone to capture long‐range interactions and intricate patterns among spots. To enhance the conditioning effect, the embeddings of the higher‐quality modality are further processed through a Multi‐Layer Perceptron (MLP), generating refined representations to serve as the conditional input. This design combines the systematic refinement of noisy modality and the preservation of spatial dependencies, ensuring that the reliable information contained in the higher‐quality modality is effectively leveraged to guide the denoising of the lower‐quality modality, thereby resulting in denoised embeddings that are accurate and biologically interpretable. Finally, the pre‐trained decoder maps the enhanced embeddings of the lower‐quality modality back to the original feature space, enabling effective reconstruction of the lower‐quality modality data.

In the integration stage (Figure 1b), CANDIES constructs a joint low‐dimensional representation by taking the enhanced spatial multi‐omics data as input. To capture the shared biological signals among features, we construct a feature similarity graph for each modality with KNN. A dual Graph Convolutional Networks (GCN) [30] is then employed to integrate information from the feature graph and the spatial graph, producing latent embeddings for each spot within each modality. Unlike existing methods that directly work with the original omics data, CANDIES operates on the refined omics dataset with reduced noise, allowing the feature proximity to be better represented. To optimally align heterogeneous multi‐modal information, contrastive learning is leveraged to encourage similarity of spatial and feature embeddings between the spots of different modalities while allowing heterogeneity between non‐matching spots. This ensures that shared biological signals are captured while preserving modality‐specific variations. Furthermore, an attention mechanism is introduced to integrate spatial and feature information by dynamically adjusting the contributions of the spatial and feature graphs. Such flexibility enables the model to adaptively determines the relative importance of spatial coherence and feature‐level similarity. Finally, CANDIES uses spatial and feature graphs to reconstruct the expression profiles, enforcing the learned latent representations to preserve original expression patterns.

### 2.2 CANDIES Enhances Spatial Domain Segmentation via Denoising on Simulated Spatial Multi‐Omics Data

We conducted a comprehensive benchmarking study to evaluate the performance of CANDIES against several state‐of‐the‐art approaches using simulated data. Following SpatialGlue [25], we generated an RNA modality with lower quality and a protein modality with higher quality (see details in “Data description and preprocessing” section). We considered two types of noise for the RNA modality. First, we perturbed the underlying true gene expression levels by adding a Gaussian noise with standard deviation varied at [2,5]. Second, based on the perturbed expression, we simulated the observed gene expression levels with a Zero Inflated Negative Binomial (ZINB) distribution while varying the probability of zero inflation within [0.1,0.4] to mimic the dropout rate. For the protein modality with higher quality, we introduced a lower level of Gaussian perturbation with standard deviation of 1 and simulated its observed values with Negative binomial distribution without dropouts (detailed parameters are in Tables. S3 and S4). We first demonstrated the denoising capacity of CANDIES by performing spatial domain identification using the latent embeddings derived from the refined RNA modality in its first step. We compared our method with various representative ST analysis methods, including GAAEST [32], GraphST [19], SEDR [20], SCAN‐IT [33], SpaceFlow [34], SpaGCN [18], STAGATE [35], BANKSY [36], and DECIPHER [37]. The performance was assessed using commonly used clustering evaluation metrics, including Mutual Information (Mutual_Info), Normalized Mutual Information (NMI), Adjusted Mutual Information (AMI), V‐measure, Homogeneity, Completeness, Adjusted Rand Index (ARI), and Fowlkes‐Mallows Index (FMI).

**FIGURE 2:**
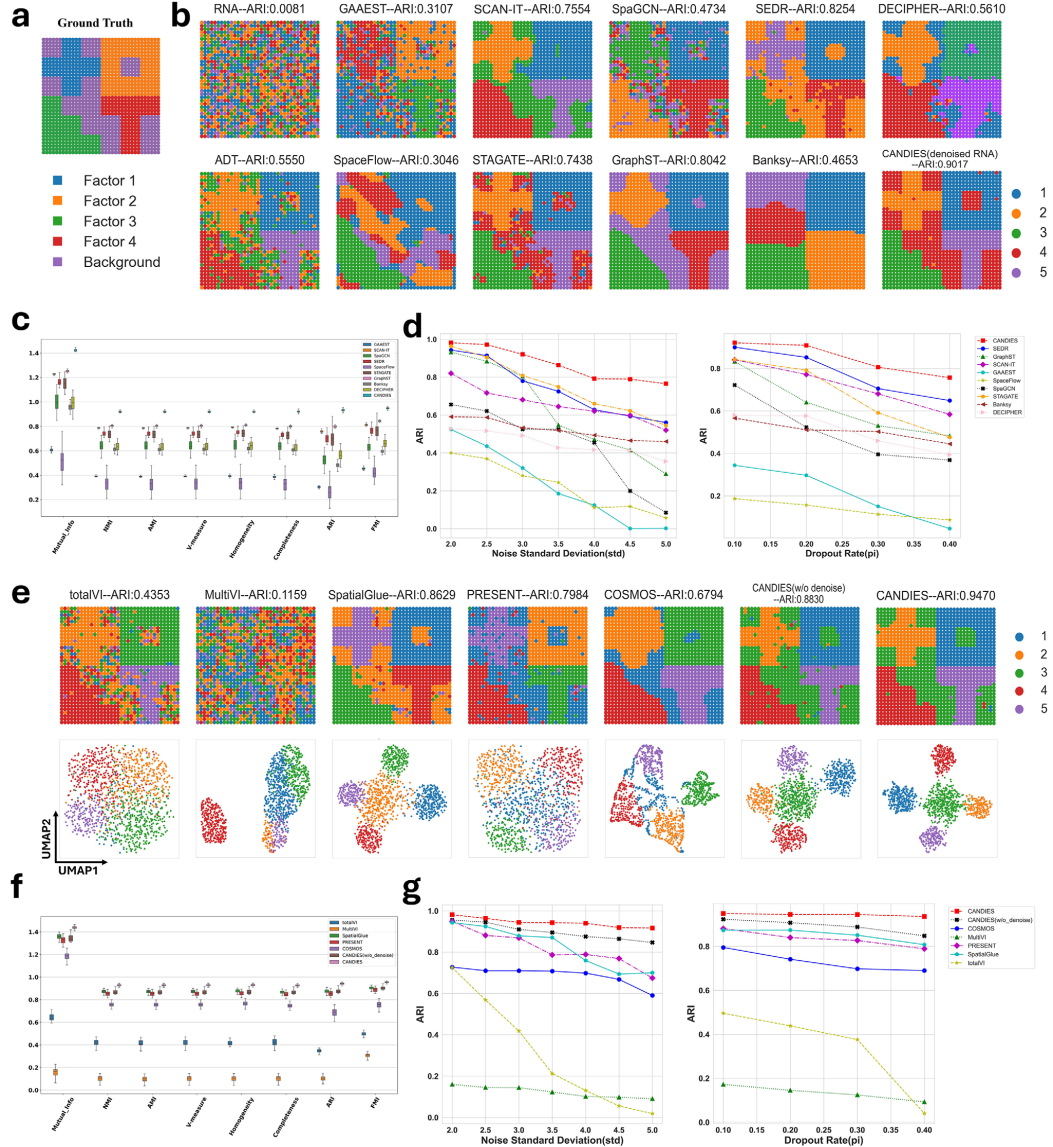
CANDIES accurately identified spatial domains in simulated spatial multi‐omics dataset. (a) Ground truth of the simulated spatial multi‐omics data. (b) Spatial plots of the simulated data with only RNA modality across nine methods designed for spatial transcriptomics (ST) data, the first column is identified by Leiden [31] on each modality. (c) Box plots of the eight supervised metrics across the ten ST analysis methods. (d) ARI scores with varying noise levels across ten ST methods. (e) Spatial plots and UMAP visualization of the simulated data across six single‐cell and spatial multi‐omics integration methods, CANDIES (w/o denoise) refers to the variant of CANDIES without the denoising phase. (f) Box plots of the eight supervised metrics across six methods. (g) The impact of varying noise levels on ARI across six single‐cell and spatial multi‐omics integration methods.

Figure 2b presents the results in the synthetic dataset with a dropout rate of 0.2, a standard deviation of 3.0 for the RNA modality, and a standard deviation 1.0 for the protein modality. The RNA modality exhibited no discernible spatial pattern, yielding a very low ARI. Leveraging its denoising mechanism, CANDIES effectively recovered all four spatial factors of the ST data, achieving an ARI of 0.92. Compared with the runner‐up, SEDR, CANDIES improved the ARI score by 11%, demonstrating its superior denoising capability through the integration of protein modality information. Besides, CANDIES and SEDR achieved higher ARI scores compared to other ST analysis methods without denoising mechanism, indicating that denoising ST data indeed enhances spatial domain identification. A box plot summarizing the performance of all methods across all evaluation metrics over thirty independent trials is shown in Figure 2c, suggesting that CANDIES stably outperformed compared methods across different evaluation metrics. Compared to SEDR and STAGATE, CANDIES' performance had little variability across different trials, indicating its high accuracy and robustness. To evaluate the impact of data quality on CANDIES denoising performance, we examined the ARI scores under increasing levels of noise (Figure 2d, left) and dropout rates (Figure 2d, right) in RNA modality. The performance of all exiting methods degraded substantially as the noise level increased. However, by effectively leveraging the higher‐quality protein modality, CANDIES exhibited a smaller performance decline compared to other methods, maintaining a superior and robust performance even under high‐noise conditions. As a comparison, although SEDR performed well under low noise levels and dropout rates, its accuracy dropped dramatically as data quality deteriorated.

With the promising results of CANDIES in denoising phase, we assessed the performance of CANDIES in multi‐omics integration by comparing it with several representative methods, including two non‐spatial multi‐omics methods: totalVI [15] and MultiVI [14], and three recently proposed spatial multi‐omics methods: SpatialGlue [25], PRESENT [26], and COSMOS [27]. Under a moderate noise level (i.e., noise with a standard deviation of 3.0 and a dropout rate of 0.2), CANDIES achieved the highest ARI score of 0.9470, successfully separating all factors without overlap (Figure 2e), such results demonstrated its superior integration capability. In particular, compared to the domain identification results in Figure 2b, which was based only on the refined RNA modality, CANDIES achieved higher improvement by integrating the high‐quality modality and the refined low‐quality modality. As can be seen, CANDIES consistently outperformed the multi‐omics methods across various metrics (Figure 2f), demonstrating stable integration performance. Notably, spatial multi‐omics methods generally achieved better results with lower variance, suggesting that incorporating the tissue microenvironment plays an important role in capturing biologically meaningful structures. Moreover, CANDIES outperformed other multi‐omics integration methods as well as its ablation variant, CANDIES (w/o denoise), which performs integration without the denoising step. When the noise level in the RNA modality was varied (Figure 2g), the inconsistency in data quality across modalities significantly affected the performance of existing spatial multi‐omics methods. In contrast, CANDIES consistently achieved the highest and most stable ARI score under all conditions, underscoring the critical role of denoising in spatial multi‐omics analysis. To investigate the impact of our denoising module, we extended the experiments in Figure 2g by introducing higher noise levels in RNA modality. The results, shown in Figure S11, clearly illustrated that the performance gap between CANDIES and the variant without denoising component widened with the increasing of noise level. As the noise level increased, the performance difference between the two variants became increasingly pronounced, demonstrating that the denoising module played a more critical role under high‐noise conditions. These findings highlighted that the denoising module was particularly beneficial in challenging scenarios with higher technical noise, thereby enhancing the robustness and reliability of spatial multi‐omics integration.

Finally, to assess the robustness of CANDIES when both modalities are of comparable quality, we conducted additional simulation experiments in which both modalities are noisy. The results in Figure S12 showed that, despite the difficulty in distinguishing quality differences between the two noisy modalities, CANDIES still demonstrated strong denoising and integration performance compared to the baseline methods. Furthermore, we considered a scenario where the relative qualities of the input modalities were incorrectly determined by treating the higher‐quality modality as the target of diffusion‐based denoising while conditioning on the lower‐quality modality. As shown in Figure S13, even under this unfavorable setting, the high‐quality modality still exhibited a certain degree of improvement, indicating that the diffusion model itself has inherent denoising capability. We further investigated the robustness of CANDIES through hyperparameters sensitivity analysis (see details in “Sensitivity analysis of hyperparameters” section).

### 2.3 CANDIES Unveils Detailed Spatial Structures on Real‐World Spatial Transcriptome‐Epigenome Data

**FIGURE 3:**
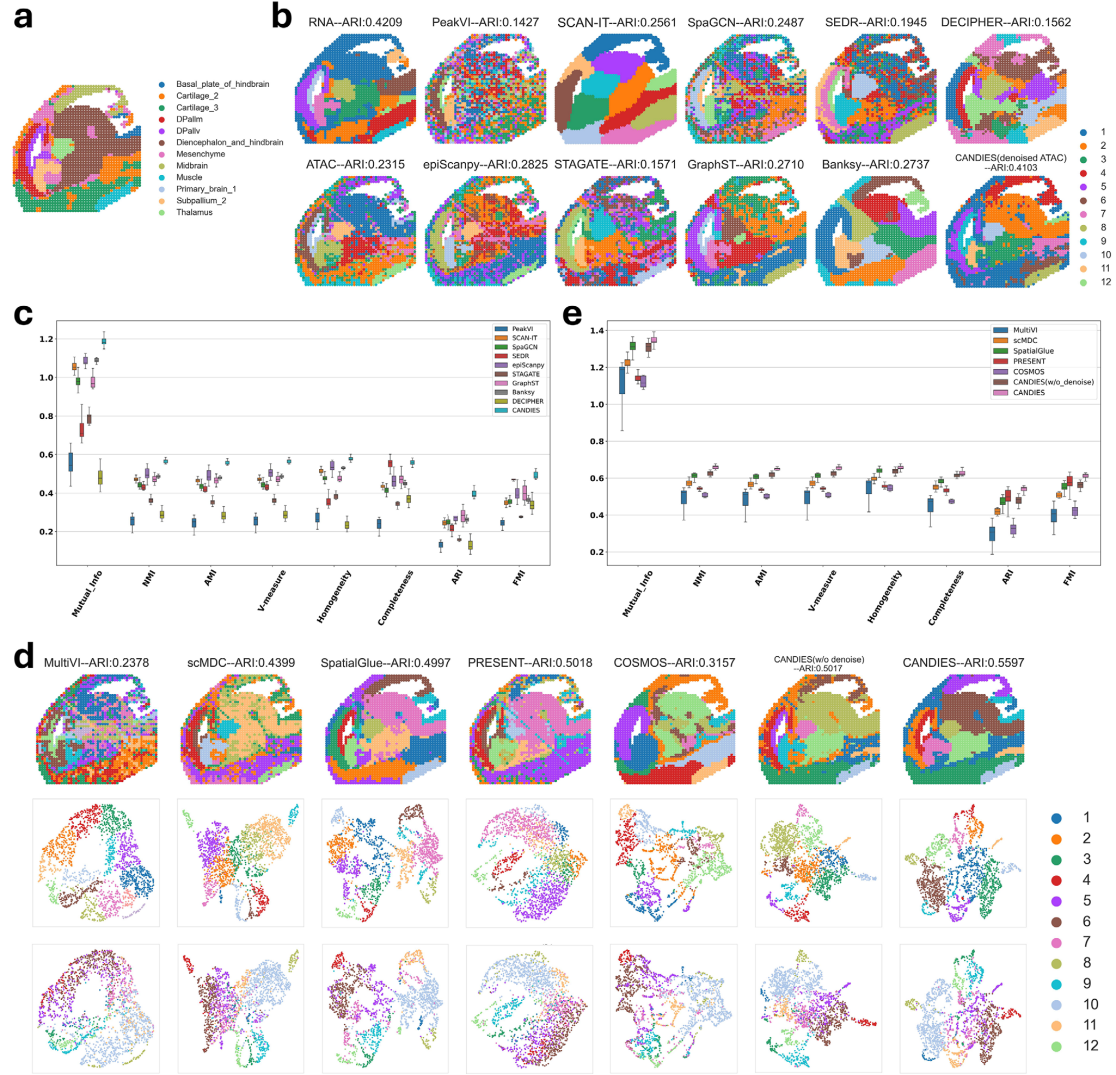
CANDIES unveils detailed spatial structures on E15.5 mouse brain data. (a) Manual annotations of the E15.5 mouse brain section. (b) Spatial plots of the E15.5 mouse brain ATAC modality across the nine representative methods, the first column is identified by Leiden on individual modality. (c) Box plots of the eight supervised metrics across ten clustering methods. (d) Spatial plots and UMAP visualization of the E15.5 mouse brain data across six single‐cell and spatial multi‐omics integration methods, the second row is colored by clusters identified by each method, and the third row is colored by manual annotations. (e) Box plots of the eight supervised metrics across six integration methods.

We first applied CANDIES to a real transcriptome‐epigenome dataset generated using MISAR‐seq platform [7], which includes sequencing data from eight mouse brain samples across four key developmental stages: E11.0, E13.5, E15.5, and E18.5. In order to evaluate the performance of denoising phase in CANDIES, we specifically focused on the E15.5 mouse brain data with manual anatomical annotation provided (Figure 3a). According to the original study, the annotations were made to highlight the major tissue organization, with the reference to Kaufman's Atlas of Mouse Development and Allen Brain Atlas. As demonstrated in the first column of Figure 3b, the RNA modality exhibited better clustering performance than the ATAC modality, characterized by well‐defined and clearer region boundaries. Therefore, we leveraged the RNA modality as the condition in the denoising phase of CANDIES to improve the quality of the ATAC modality.

To evaluate the effectiveness of the denoising framework in CANDIES, we compared it with two widely used single‐cell ATAC clustering methods, PeakVI [38] and epiScanpy [39], ensuring a fair and consistent benchmarking setup. As shown in Figure 3b, most baseline methods, including PeakVI, epiScanpy, SEDR, and STAGATE, failed to accurately delineate the major tissue regions of the mouse brain, exhibiting even poorer performance than the Leiden clustering result applied directly to the original ATAC modality. These methods struggled with the complex and inherent noise, leading to misclassified boundaries between tissue regions. In contrast, the denoised result produced by CANDIES not only provided clearer boundaries, but also successfully identified most of the key regions, such as the midbrain (cluster 4), DPallm (cluster 5), muscle (cluster 8), DPallv (cluster 9), thalamus (cluster 10), and subpallium_2 (cluster 11). This improvement was further supported by the superior metrics reported in Figure 3c, which highlight the effectiveness of CANDIES in unveiling detailed spatial regional structures by leveraging the complementary information from the modality with higher quality.

Next, we compared CANDIES' integration performance with several spatial multi‐omics integration methods, including SpatialGlue, PRESENT, and COSMOS, as well as the single‐cell integration methods such as MultiVI and scMDC [40]. TotalVI was not included as it is specifically designed for single‐cell RNA‐ADT data. As shown in Figure 3d, MultiVI struggled with fine spatial distinctions, particularly failing to identify the major regions such as diencephalon_and_hindbrain, thalamus and muscle. Similarly, scMDC showed some improvement but still poorly delineates regions such as midbrain. Among spatial multi‐omics methods, SpatialGlue performed moderately better, showing clearer boundaries in regions like the midbrain, DPallm, DPallv, and muscle, but still suffered from some noise and overlapping. In contrast, CANDIES outperformed all other methods with an ARI of 0.5597, clearly delineating major regions such as the DPallm (cluster 2), DPallv (cluster 4), midbrain (cluster 5), and thalamus (cluster 8), while accurately clustering finer regions like subpallium_2 (cluster 7). The superior performance of CANDIES was also reflected in its higher scores in other metrics (Figure 3e), indicating the effectiveness of integration with denoised modalities. To further investigate the role of denoising, we carried out ablation analysis by applying CANDIES without the denoising phase, denoted as CANDIES (w/o denoise). It turns out that the ARI was reduced to 0.5017, which had similar performance with PRESENT. These results demonstrate that the denoising and integration modules jointly contribute to effectively unveiling spatial domains. A similar conclusion was further validated through additional experiments on the E18.5 dataset (Figures S3 and S4), confirming the robustness and generalizability of CANDIES across different developmental stages.

### 2.4 CANDIES Captures Fine‐Grained Spatial Domain with Denoising on Spatial Transcriptome‐Proteome Data of a Human Skin Biopsy from a COVID‐19 Vaccine Injection Site

**FIGURE 4:**
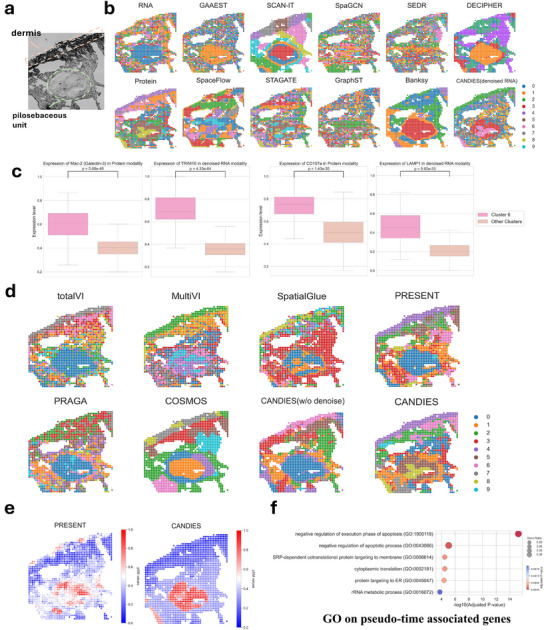
CANDIES achieves fine‐grained spatial partitioning on the human skin dataset generated using the spatial CITE‐seq technique. (a) The bright‐field image of the human skin tissue section. Highlighted regions are dermis and pilosebaceous unit. (b) Spatial plots of the human skin data only with RNA modality across nine representative spatial transcriptomics (ST) methods, the first column is identified by Leiden on individual modality. (c) Expression levels of *Mac‐2 (Galectin‐3)* and *TRIM16* across Protein and denoised RNA modalities in the left of box plots, as well as *CD107a* and *LAMP1* in the right of box plots. Cluster 6 was identified by the denoised RNA modality, and p‐values were calculated using the Mann‐Whitney U test. (d) Spatial plots of the human skin data across seven single‐cell and spatial multi‐omics integration methods. (e) Pseudo spatiotemporal maps (pSM) generated by CANDIES and PRESENT. (f) Gene Ontology Biological Process (GOBP) enrichment analysis on pseudo‐time associated genes.

Given the promising denoised results on E15.5 mouse brain data, we applied CANDIES to analyze spatial co‐profiling data of transcriptome and proteome obtained from skin biopsy tissue after COVID‐19 mRNA vaccination generated by spatial CITE‐seq technique [3] to identify new biological insights with denoising module. The tissue section contains a collagen‐rich dermis region with low cell density and a pilosebaceous unit characterized by high cellularity (Figure 4a). The competition between ADTs and mRNAs for in‐tissue reverse transcription reduces the efficiency of transcript detection [3], making the RNA modality highly noisy. Therefore, although the overall structures of the dermis and pilosebaceous unit were clearly discernible in the protein modality, these anatomical features appeared less distinguishable in the RNA modality (Figure 4b). The inherent noise in the RNA modality made it difficult to reliably identify biological clusters with existing methods. For example, SCAN‐IT, SpaGCN, GraphST, and STAGATE exhibited significant noise and failed to clearly identify the major regions. In contrast, CANDIES effectively leveraged the high‐quality protein modality for denoising, enabling accurate identification of the dermis and the pilosebaceous unit. We contrasted the spatial expression patterns of differentially expressed genes (DEGs) before and after denoising on the RNA modality of the human skin data (Figure S14). Among the identified top‐2 DEGs for each cluster from the denoised RNA data, we found multiple lines of evidence aligned with prior knowledge. Notably, these DEGs were not detectable in the raw data and were recovered after the denoising step. Consistent with the original study [3], the transmembrane protein‐encoding gene *TMEM132D* in cluster 2 showed specific expression in the dermis region, while the *CYP450* protein‐encoding gene *CYP4F8* was broadly expressed across most skin regions. Additionally, *MTRNR2L1* and *MTRNR2L12* showed specific enrichment in the pilosebaceous unit (cluster 3 and cluster 6). Notably, the spatial expression patterns of the four selected DEGs became substantially more distinct in the denoised RNA modality compared to their original profiles, demonstrating that CANDIES' denoise step effectively captures meaningful biological signals.

As shown in Figure 4b, it is evident that cluster 6 is not detected in the RNA modality, likely due to the inherently low expression level in the original RNA measurements. By leveraging information from the protein modality, CANDIES effectively recovered the underlying biological signal and uniquely identified cluster 6 in the denoised RNA modality. To assess the biological relevance of the clusters identified from the denoised RNA modality, we performed a domain‐specific expression analysis on cluster 6 and found multiple lines of evidence aligned with prior knowledge (Figure 4c). We first identified the top‐2 differentially expressed proteins in the ADT modality, i.e., *Mac‐2 (Galectin‐3)* and *CD107a*. As shown in Figure 4c, both differentially expressed proteins exhibited significantly higher expression levels in cluster 6 compared to the other clusters, with p‐values of $5.68\times 10^{-49}$ for *Mac‐2 (Galectin‐3)* and $1.40\times 10^{-35}$ for *CD107a*, respectively. In the denoised RNA modality, *CD107a*'s coding gene, *LAMP1*, was differentially expressed in the cluster 6 with a p‐value of $9.82\times 10^{-33}$. This protein plays a critical role in protecting natural killer cells from degranulation‐associated damage [41]. Besides, we found that *TRIM16*, which interacts with *Mac‐2 (Galectin‐3)* to mediate autophagic protection against lysosomal damage [42], was also differentially expressed in cluster 6 with a p‐value of $4.33\times 10^{-64}$ after denoising. Based on gene and protein expression, cluster 6 likely represents an immune‐responsive niche within the pilosebaceous unit of human skin. In the context of COVID‐19 vaccination, this activity may indicate a localized immune response, potentially involving the activation of innate immune pathways [43, 44]. This strong concordance observed between protein and gene expression in cluster 6 indicates that the denoising process performed in CANDIES effectively captured the biological relationships, thereby utilizing the critical protein‐level information to refine the RNA modality.

We noted that clusters 3 and 6 together constitute the pilosebaceous unit, corresponding well to the blue domain identified in the RNA modality‐based clustering results. To further highlight the distinctive characteristics of cluster 3 and cluster 6 in RNA modality, we identified two key differentially expressed genes in each cluster: *RASSF5* and *CTSZ* in cluster 6, and *SOAT1* and *UBC* in cluster 3. The expression levels of these four genes were shown in Figure S7. Specifically, *RASSF5* belongs to the Ras association domain family and functions as a tumor suppressor frequently inactivated in various cancers. It regulates key cellular processes including proliferation, cell cycle progression, microtubule stability, and apoptosis [45, 46]. *Cathepsin Z (CTSZ)* is a lysosomal cysteine protease within the papain superfamily, involved in immune defense through phagocytosis, signal transduction, intercellular communication, and migration of monocytes, macrophages, and dendritic cells [47]. Notably, the downregulation of *CTSZ* in human keratinocytes leads to elevated *EGFR* expression [48], which was consistent with the results shown in Figure S7c. In contrast, *SOAT1*, localizes to the endoplasmic reticulum and enzymatically esterifies cholesterol into cholesteryl esters as dimers or tetramers [49]. *SOAT1* has been found to play a crucial role in modulating cholesterol levels and maintaining lipid homeostasis, processes essential for sebaceous gland function and epidermal barrier integrity [50]. *Ubiquitin C (UBC)* is an essential mammalian polyubiquitin gene responsible for generating ubiquitin monomers, thereby maintaining ubiquitin (Ub) homeostasis, and is known to be upregulated under cellular stress conditions [51]. Together, these findings indicated that the two transcriptional niches identified by CANDIES likely serve distinct physiological roles. Cluster 6 may represent a regulatory niche involved in immune surveillance, suppression of hyperproliferation, and maintenance of epidermal homeostasis, whereas cluster 3 specializes in lipid metabolism and protein homeostasis, supporting sebum production and cellular stress adaptation within the pilosebaceous unit.

Next, we comprehensively benchmarked CANDIES' integration performance with totalVI, MultiVI, SpatialGlue, PRESENT, PRAGA, and COSMOS on spatial transcriptome‐proteome human skin data (Figure 4d). We also included a simplified version of CANDIES, i.e., CANDIES (w/o denoise), that takes the original modalities as input, allowing us to investigate the impact of denoising step. Without incorporating spatial information, totalVI and MultiVI produced less smooth boundaries between spatial domains. Meanwhile, spatial multi‐omics methods such as SpatialGlue and PRAGA failed to depict the dermal architecture since they do not consider the huge differences of data quality across modalities. PRESENT showed relatively better performance, successfully identifying the main regions, e.g., the dermis region and the pilosebaceous unit, but still exhibited limitations in handling fine‐grained details. The clustering patterns produced by CANDIES more closely resembled those identified on individual modality. For example, cluster 8 was only identified by CANDIES but remained undetected by PRESENT, MultiVI, and CANDIES without denoising, validating the effectiveness of denoising in distinguishing fine‐grained biological patterns.

Furthermore, to investigate the spatiotemporal dynamics across different regions, we leveraged CANDIES clustering results to generate a pseudo‐Spatiotemporal Map (pSM) for the skin biopsy [52] (Figure 4e), using PRESENT as a baseline for comparison (the pSM results for all the baseline methods were provided in Figure S10). For visualization, the pseudo‐spatiotemporal map (pSM) uses a color gradient from blue to red to represent early to late stages along the developmental process. We observed that the pSM values of CANDIES were the lowest in the dermal region and exhibited a gradual increase toward the pilosebaceous unit, reflecting a plausible spatial developmental trajectory from dermal to pilosebaceous. This spatial gradient in pSM values aligned with the known developmental sequence of these structures, where the dermis serves as the origin for sweat glands, sebaceous glands, apocrine glands, and hair follicles [53]. In contrast, as shown in Figure S10, totalVI and MultiVI failed to capture a clear spatial development process in pSM values, as these methods did not explicitly incorporate spatial information during model training. Among spatial‐aware methods, SpatialGlue and PRESENT exhibited partial development patterns. However, the resulting pSM values remained noisy and less well‐aligned with known developmental sequence of these regions, likely due to the intrinsic noise in RNA modality. Importantly, the ablation analysis of CANDIES (w/o denoise) further highlighted the contribution of diffusion‐based denoising. Although CANDIES (w/o denoise) recovered a coarse spatial trend, the pSM values were noticeably less smooth and less distinct compared with the full model. In addition to further understand the biological processes underlying the spatiotemporal dynamics, we performed Gene Ontology (GO) enrichment analysis on the pseudo‐time associated genes (Figure 4f). The GO terms with the most significant enrichment included negative regulation of apoptosis, SRP‐dependent cotranslational protein targeting to membrane, and cytoplasmic translation. These processes suggest that the pseudo‐time associated genes play crucial roles in regulating apoptosis and cellular translation, which are essential for skin cell differentiation and maturation [54, 55].

### 2.5 CANDIES Facilitates the Integrated Analysis of Spatial H3K27ac‐H3K27me3 and Epigenome‐Transcriptome Data from Mouse Embryo

To demonstrate applicability of CANDIES across diverse sequencing platforms, we applied CANDIES to integrate spatially resolved H3K27ac–H3K27me3 and epigenome–transcriptome datasets. The spatial H3K27ac–H3K27me3 and epigenome–transcriptome datasets are two spatially resolved mouse embryo datasets generated using spatial‐Mux‐seq [17] and spatial ATAC–RNA‐seq [6], respectively, representing distinct noise patterns introduced by different sequencing technologies.

**FIGURE 5:**
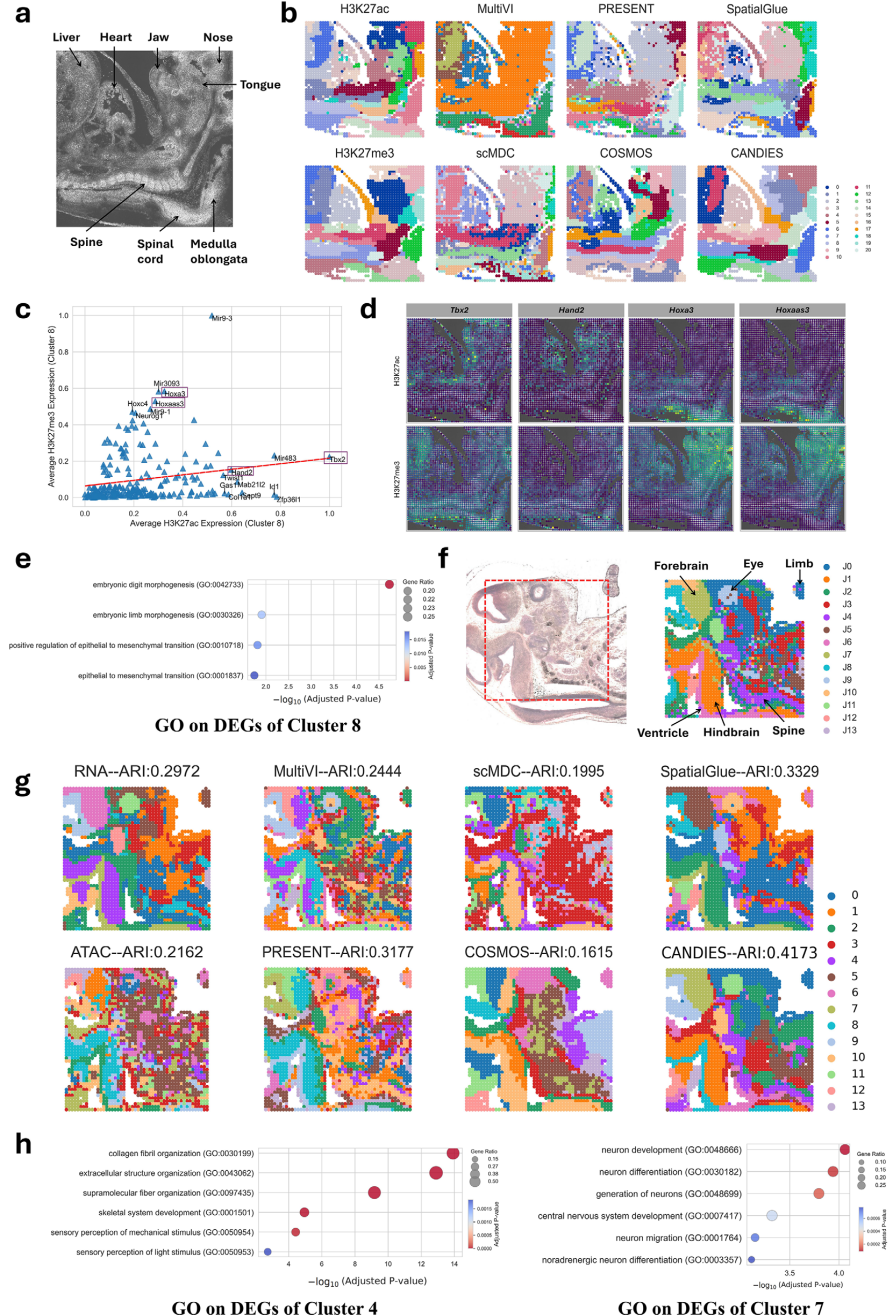
CANDIES accurately integrated E13 mouse embryo spatial multi‐omics data across different sequencing technologies. (a) Manual annotation of the E13 sagittal mouse embryo data generated by spatial‐Mux‐seq technology. (b) Spatial plots of the E13 mouse embryo data across six single‐cell and spatial multi‐omics integration methods, the first column is identified by Leiden on each individual modality. (c) Correlation plot of average H3K27ac and H3K27me3 expressions across overlapping DEGs in cluster 8. (d) Spatial expression distribution of the selected four DEGs across H3K27ac (top) and H3K27me3 (bottom) dataset. (e) Gene Ontology (GO) enrichment analysis on DEGs of cluster 8. (f) The bright‐field image (left) and the manual annotation (right) of the E13 mouse embryo data sequenced by spatial ATAC–RNA–seq platform. (g) Spatial plots of the E13 mouse embryo data across six single‐cell and spatial multi‐omics integration methods, the first column is identified by Leiden on each individual modality. (h) GO enrichment analysis on DEGs of cluster four (left) and cluster seven (right).

We first applied CANDIES to a E13 sagittal mouse embryo section generated by spatial‐Mux‐seq platform, which co‐profiles two mutually exclusive histone modifications: H3K27ac and H3K27me3. First, we compared CANDIES with the spatial multi‐omics integration methods, such as SpatialGlue, PRESENT, and COSMOS, as well as the single‐cell methods MultiVI and scMDC. To determine the relative quality of the two modalities, we applied Leiden method on the low‐dimensional embeddings of each modality obtained from a pre‐trained graph auto‐encoder and evaluated their quality using four unsupervised metrics (details in Materials and Methods). As shown in the first column of Figure 5b, it was clear that the H3K27me3 modality demonstrated better performance, as it precisely captured the core regions with well‐defined boundaries. This observation was quantitatively supported by the unsupervised metrics. As shown in Table. S7, the H3K27me3 modality consistently achieved better clustering quality than H3K27ac across these metrics, including higher Silhouette, Calinski‐Harabasz Index (CHI) and Moran's I scores, lower Davies‐Bouldin Index (DBI) value, indicating H3K27me3 modality exhibited higher structural consistency and spatial organization. Therefore, we used the H3K27me3 modality as the condition to refine the quality of the H3K27ac modality in the denoising phase of CANDIES.

As shown in Figure 5b, CANDIES demonstrated superior performance in both the detection of major domains and the preservation of rare spot types. Specifically, CANDIES successfully distinguished major anatomical categories, including spinal cord (cluster 2), heart (cluster 3), medulla oblongata (cluster 6), and liver (cluster 11), while also preserving the integrity of rare types such as tongue (cluster 1), nose (cluster 4) and jaw (cluster 8). In contrast, other methods exhibited notable limitations. For example, since single‐cell multi‐omics methods such as MultiVI and scMDC do not incorporate spatial information, their clustering results showed poor spatial continuity. This led to fragmented spatial distributions, where adjacent regions were often incorrectly assigned to different clusters, failing to accurately represent the true tissue organization. Spatial multi‐omics methods demonstrated improved performance; however, their inability to effectively handle noise sometimes led to incorrect partitioning of regions. For instance, PRESENT successfully identified liver and heart, but it erroneously merged the jaw and tongue into a single region. Similarly, COSMOS showed moderate performance, yet incorrectly clustered the spots within the craniofacial region (e.g., jaw, tongue, and nose), fragmenting them into multiple smaller regions that did not align with anatomical structures.

To investigate the biological relevance of the new regions identified by CANDIES, we focused on cluster 8, annotated as the jaw region. Notably, cluster 8 was not detected in either single modality (H3K27ac or H3K27me3) but emerged only when the two modalities were integrated. In particular, we first identified the overlapped DEGs in cluster 8 across both H3K27ac and H3K27me3 modalities based on their original count data. In Figure 5c, the x‐axis represented the average expression of H3K27ac in cluster 8, while the y‐axis represented the average expression of H3K27me3 in cluster 8, and the red dashed line corresponded to a least‐squares linear regression fitted to the data points, highlighting the strong correlation between H3K27ac and H3K27me3 expression levels in cluster 8. The analysis results are consistent with previous studies. Specifically, *Hoxa3* and *Hoxaas3* were enriched for H3K27me3 but not H3K27ac in this cluster. *Hoxaas3*, a long non‐coding RNA, is known to frequently function through interaction with *Hoxa3* [56]. The expression domains of *Hoxa3* broadly correspond to the sites of cranial neural crest cells (CNCCs) origin, which plays a significant role in the development of the jaw and related structures [57]. In line with this, mutant mice lacking *Hoxa3* exhibit malformations of jaw bones and throat cartilages [58]. Regarding DEGs enriched in H3K27ac modality, *Tbx2* is a member of T‐box transcriptional regulator family, which is involved in all stages throughout the embryonic development, including craniofacial, brain, heart, skeleton, and immune system [59]. Additionally, as reported in the original study [17], *Hand2* is an important transcription regulator in craniofacial development [60], which was enriched for H3K27ac but not H3K27me3 in the jaw region. Notably, the spatial expression pattern of *Hand2* is consistent with this observation (Figure 5d). Furthermore, based on the identified DEGs in H3K27ac modality, we performed the GO enrichment analysis (Figure 5e), which revealed several enriched biological processes related to jaw development, such as embryonic digit morphogenesis, embryonic limb morphogenesis, and positive regulation of epithelial to mesenchymal transition (EMT). Specifically, the positive regulation of EMT suggests that the jaw region may undergo CNCCs delamination via EMT processes, which aligns with prior researches [61, 62]. These results further support the biological relevance of the jaw region identified by CANDIES.

Furthermore, we applied CANDIES to integrate additional E13 mouse embryo epigenome‐transcriptome data generated from spatial ATAC–RNA–seq [6] platform. According to the original study [6], the authors performed spatial ATAC–RNA‐seq experiments on E13 mouse embryo tissue at a pixel size of 50 μm. They identified a total of 2,187 spots, which were further grouped into 14 RNA clusters based on specific marker genes. The spatial distribution of these clusters aligned well with tissue histology, covering regions such as the embryonic eye field, several developing internal organs, the ventricle, and the central nervous system (CNS). Therefore, we used these regional annotations as reference (Figure 5f, right), including the forebrain, eye, limb, ventricle, hindbrain, and spine. We observed that the RNA modality captured core regions with better‐defined boundaries than those in the ATAC modality, suggesting a better quality of the transcriptome profiles (Figure 5g, first column), which was consistent with the original study. Therefore, we utilized the RNA modality to enhance the ATAC modality in this data. As shown in Figure 5g, CANDIES produced the most accurate spatial domain identification among compared methods. MultiVI, SpatialGlue, and PRESENT generated less distinct clustering patterns with substantial overlap across different domains. In contrast, CANDIES effectively identified all major anatomical regions including hindbrain (cluster 2), spine (cluster 6), ventricle (cluster 8), and eye tissues (cluster 11), achieving the highest ARI score of 0.4173, which is 8.44% higher than the runner‐up.

Finally, to characterize the biological significance of the domains identified by CANDIES, we performed GO enrichment analysis (Figure 5h) with differentially expressed genes in two regions: cluster 4 (annotated as spine) and cluster 7 (annotated as forebrain). For each cluster, we identified 20 DEGs and performed GO enrichment analysis. We identified significant enrichment that aligns with the biological function of the corresponding clusters. Specifically, cluster 7, corresponding to forebrain, showed pronounced enrichment in fundamental neuro‐developmental processes, including generation of neurons, neuron differentiation, neuron development, and neuron migration, which are closely related to the forebrain's well‐established role in neurogenesis and cortical circuit formation [63]. For cluster 4, we found it strongly associated with the spine development process and enriched in pathways related to bone morphogenesis, particularly collagen fibril organization, and skeletal system development. These cluster‐specific GO enrichment results suggest that CANDIES successfully extracted biologically meaningful signals by integrating ATAC–RNA–seq data.

### 2.6 CANDIES Improves the Mapping of Complex Traits to Relevant Spatial Domains

Although the advancement of spatial omics has provided valuable insight into the spatial landscape of a cascade of biological process across a wide range of tissues, most of the available spatial data resources are not pertinent to a specific trait of interest. Integrating spatial omics data with complex trait GWASs is expected to offer a comprehensive characterization of how the spatial distribution of cells are associated to disease pathology. However, the strong noise present in spatial omics data makes it difficult to establish a reliable trait‐spot link. In this section, we show that our spatially informed cross‐modality embeddings produced by CANDIES can improve the mapping of trait‐spot associations. To illustrate this benefit, we carried out gsMap [64] analysis by taking the embeddings of CANDIES as input to identify the trait‐spot associations across three tissues, including mouse brain, mouse embryo, and human lymph, and 32 complex traits encompassing five broad biological clusters. Briefly, to link polygenic GWAS signals to spatial locations, we define each spot as an annotation of a set of single nucleotide polymorphisms (SNPs) based on its gene specificity score (GSS), and estimate the heritability enrichment of each spot for a target trait by employing the stratified linkage disequilibrium score regression (S‐LDSC). Since each spot represents a noisy observation of the true spatial profile, we shall aggregate the information across homogeneous spots to obtain a reliable annotation. Here, we first applied CANDIES to combine multi‐omics spatial data, leading to a more robust latent representation of the original profile. Then, a homogeneous microdomain was constructed for each spot based on the cosine similarity of the CANDIES latent embeddings. Such a microdomain includes a set of spots sharing spatial and biological similarities with the focal spot across different modalities. For each gene, we estimated its GSS within each focal spot by calculating a normalized geometric mean of its expression rank across the microdomain of the focal spot. Finally, we performed S‐LDSC analysis by using each spot's GSS as the annotation. As such, the trait‐associated spots can be identified by testing whether SNPs with higher GSS are enriched for heritability.

**FIGURE 6:**
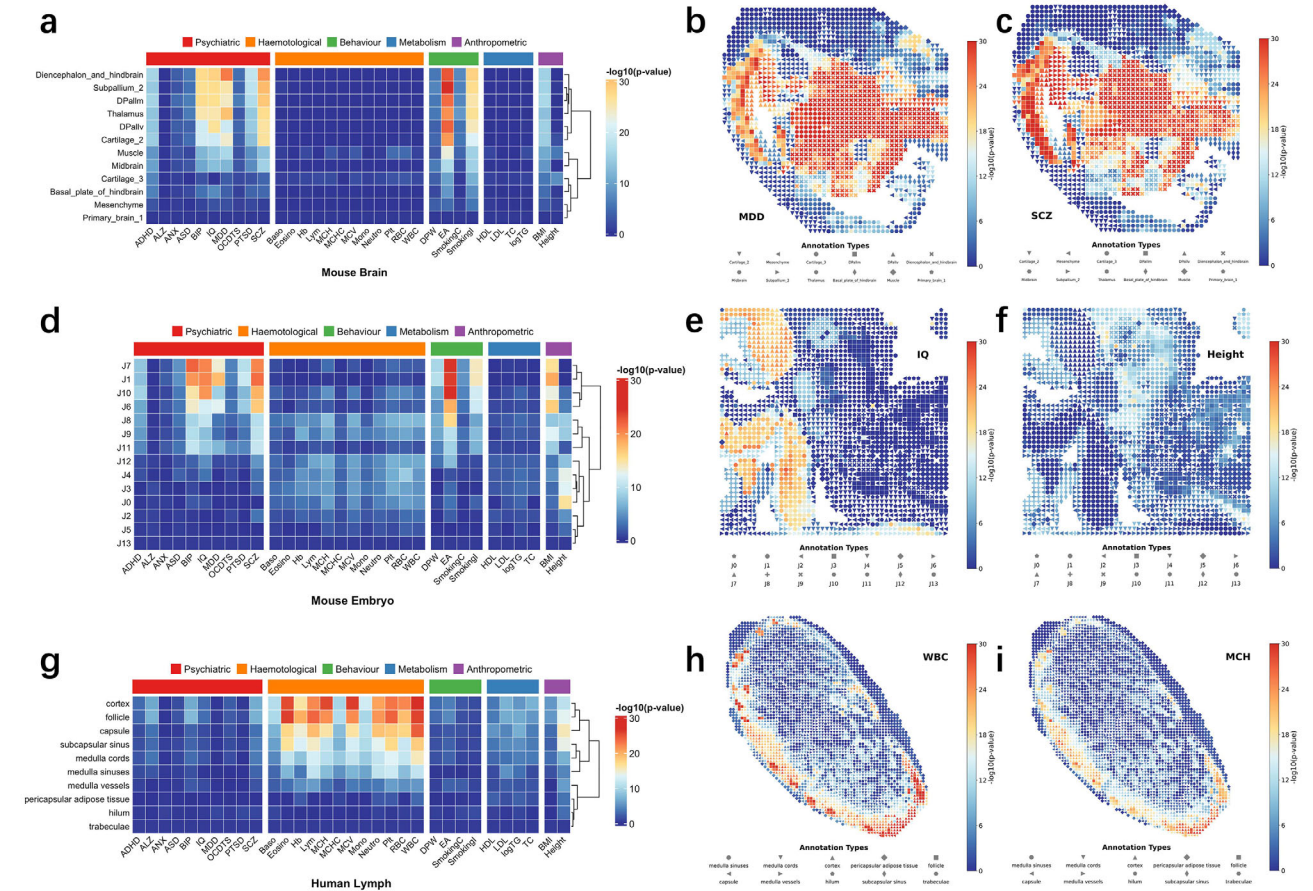
Trait‐spot associations identified by using CANDIES embeddings in three spatial omics datasets as input. (a,d,g) Heatmaps showing the significance level of associations between complex traits and spatial regions, with rows representing annotated regions in the mouse brain, mouse embryo, and human lymph respectively, and columns representing human traits categorized into five classes. p‐values are obtained with Cauchy combination test across spots within each region. Color scale indicates the $-\log_{10}p$‐values of the Cauchy combination test. (b,c) Heritability enrichment of two representative psychiatric traits, major depressive disorder and schizophrenia, in the mouse brain analysis, with spot annotation indicated by different point shapes and $-\log_{10}p$‐values reflected by the color. (e,f) Heritability enrichment of two representative traits, IQ and height, in the mouse embryo analysis, illustrating how developmental regions associate with cognition and anthropometric traits. Annotations J0–J13 is obtained from the joint clustering of spatial ATAC and RNA data, where J1 and J6 roughly map to the Hindbrain region, J7 and J10 map to the Forebrain region, J3 and J4 map to the spine, and the circular area marked by J9 maps to the eye region. (h,i) Heritability enrichment of two hematological traits, white blood cells count and mean corpuscular hemoglobin, in the human lymph analysis.

We summarized the identified trait‐associated spatial locations in Figure 6 and Figures S15–S22. Figure 6 shows the p‐values of enrichment across different spatial regions, obtained by aggregating spot‐level p‐values using Cauchy combination test. As we can observe, the enriched regions were highly aligned with our knowledge of tissue regions related to the 32 traits. For psychiatric traits, such as bipolar disorder (BIP), intelligence (IQ), major depression disorder (MDD), schizophrenia (SCZ), and behavioral phenotypes, including education attainment (EA) and smoking initiation (SmokingI), we observed significant enrichment for heritability in the dorsal pallium, thalamus, and hindbrain of the mouse brain data (Figure 6a–c, Figure S15a,b and Figure S16). In mouse embryo data (Figure 6d), psychiatric and behavioral traits were also strongly associated with regions related to CNS, which is consistent with our findings in the mouse brain data (Figure 6e; Figure S15c). Besides, height was mapped to embryo regions related to the formation of cartilage (Figure 6f). In the analysis of lymph node data, we found substantial enrichment of haemotological traits within regions of cortex, follicle, medulla, and capsule (Figure 6g–i; Figure S15). In contrast, when the embeddings were generated with only RNA modality, the lymph regions harboring immune cells were less enriched for relevant haemotological traits (Figure S15) due to the noisy nature of RNA modality. For instance, CANDIES embeddings yielded substantially higher significance for MCHC in the medulla cords (Figure S23a) and markedly stronger enrichment for Eosino and WBC in the subcapsular sinus (Figure S23b). Importantly, traits from other categories (psychiatric, behavioral, metabolic, and anthropometric) remained largely non‐significant in these immune compartments regardless of the embedding approach, serving as an effective negative control and demonstrating that CANDIES does not artificially inflate associations for biologically irrelevant traits. The three lines of evidence from different spatial multi‐omics data converged to well‐established biological relevance between traits and tissues, suggesting CANDIES embeddings successfully captured important biological information to facilitate interpreting GWAS discoveries. Given the promising results, we investigated the spatial distribution of trait associations at the spot level. We found that cells located closer to the dorsal end of the dorsal pallium exhibited a much stronger association with MDD (Figure 6b) and SCZ (Figure 6c). Previous studies have reported that such observation can be attributed to the spatially differential expression of calcium transport‐related genes in Glu‐neurons along the dorsal‐ventral axis [64]. Therefore, by integrating with GWAS data, the joint embeddings produced by CANDIES can offer a better understanding of the genetic architecture of complex human traits within spatially resolved tissue profiles.

## 3 Discussion

In this paper, we have introduced CANDIES, a novel multi‐omics integration framework based on conditional diffusion and contrastive learning‐based framework. CANDIES is able to integrate two modalities with varying qualities, thereby generating robust and unified spatial representations. Through extensive experiments, we showed that CANDIES outperforms top‐performing methods, including nine spatial single‐omic clustering methods, three single‐cell multi‐omics integration approaches, and four spatial multi‐omics integration techniques. We applied CANDIES to integrate spatially resolved multi‐omics data using six real datasets comprising varying tissue types, sequencing platforms, and noise patterns, producing high‐quality low‐dimensional representations that are versatile in multiple down‐stream biological analysis. We showed that CANDIES embeddings are highly reliable in denoising low‐quality modality, spatial domain identification and pseudo‐spatiotemporal map generation. By combing the output of CANDIES with complex trait GWAS, we illustrated that the output of CANDIES was particularly helpful to characterize the spatially‐resolved mapping of complex human traits. We believe that CANDIES will serve as a powerful analytic tool to facilitate spatial multi‐omics data analysis.

The key strength of CANDIES lies in its two‐stage framework, which first refines the lower‐quality modality using a conditional diffusion model and then integrates the enhanced data with another modality through contrastive learning. Unlike conventional spatial multi‐omics integration methods, CANDIES leverages guidance from the higher‐quality modality and spatial information based on DiT network to achieve effective noise reduction for the lower‐quality modality. During integration, CANDIES first employs graph auto‐encoders to derive both spatial and feature embeddings for each modality. Then, an innovative contrastive learning strategy is applied to align feature or spatial embeddings across different modalities. Finally, an attention mechanism dynamically balances spatial and feature information. This systematic approach not only enhances robustness and preserves cellular heterogeneity but also guarantees biologically meaningful results, positioning CANDIES as a uniquely effective solution for diverse spatial multi‐omics datasets.

In the future, there are several promising avenues for extending CANDIES. One potential direction is the incorporation of biological priors, such as known gene regulatory networks, pathway interactions, and spatial tissue organization constraints. Integrating these biologically informed relationships into the model architecture could improve both interpretability and biological fidelity, enabling the framework to better capture the underlying mechanisms driving spatial heterogeneity. In addition, we plan to extend CANDIES to integrate spatial imaging data, either during the denoising or the integration phase, to jointly model molecular and morphological information. Such multimodal integration would further enhance the analytical power of CANDIES, providing a more comprehensive understanding of tissue architecture and cellular dynamics. Additionally, we aim to expand CANDIES to support triple‐omics and single‐cell level analysis, addressing the growing complexity of multi‐omics datasets and enabling more comprehensive biological insights.

## 4 Materials and Methods

### 4.1 CANDIES Architecture

CANDIES utilized the powerful generative capabilities of diffusion models to refine the modality with lower quality, as described by Figure 1, the framework of it consisted of two main parts: denoising and integration. For better elaboration, we assumed there were two modalities, where the quality of modality 2 was superior than that of modality 1. Denoting the expression matrix of modality 1 and modality 2 are $X^{1}\in R^{N\times d_{1}}$ and $X^{2}\in R^{N\times d_{2}}$ respectively, where N represents the number of spots (or cells) in the dataset, and $d_{m}$ corresponds to the features dimension in the m‐th modality, m∈{1,2}. The spatial coordinates of these N sequencing spots (cells) are defined as $S={\left\{\left(x_{i},y_{i}\right)\right\}}_{i=1}^{N}$.

#### 4.1.1 Denoising Phase of CANDIES

In the denoising part, a pre‐trained dual graph auto‐encoder was first employed to obtain the low‐dimensional embeddings of all spots for two modalities. These embeddings were then fed into decoders to reconstruct the original expression matrix. For the embeddings of lower‐quality modality, it performed as the starting point of the diffusion process, and a DiT‐based conditional denoising model with the embeddings of the higher‐quality modality as condition was utilized to learn the reverse process, beginning from these noise‐perturbed initial embeddings.

**Construction of spatial neighbor graph**. For the given modality 1 or modality 2, we first utilized their spatial information to construct the spatial graph as $G_{s}^{m}=\left(V,E_{s},A_{s},X^{m}\right),m\in \left\{1,2\right\}$, where V denotes the set of N spots and $E_{s}$ represents the set of edges connecting these spots. $A_{s}\in R^{N\times N}$ is the adjacency matrix calculated by the spatial coordinates $S={\left\{\left(x_{i},y_{i}\right)\right\}}_{i=1}^{N}$, where $A_{s}\left(i,j\right)=1$ if the spot j is among the k‐nearest neighbors of the spots i (k=3 by default), otherwise $A_{s}\left(i,j\right)=0$.

**Pre‐trained graph auto‐encoders (GAEs) for individual modality**. Considering each modality captures complementary biological information in spatial multi‐omics data, we pretrained a dual graph autoencoder to separately encode each modality into its latent representation while incorporating spatial neighborhood structures. The dual graph autoencoder pretraining stage followed the standard paradigm adopted in latent diffusion models [65]. By projecting each modality into the latent space before denoising, it substantially reduced the computational cost associated with diffusion on high‐dimensional feature spaces such as RNA or ATAC count data. Specifically, two different encoders $E_{d}^{m},m\in \left\{1,2\right\}$, consisting of two graph convolution network (GCN) layers, were employed to generate the low‐dimensional embedding $Z_{s}^{m}\in R^{N\times d_{s}}$ from spatial graph $G_{s}^{m},m\in \left\{1,2\right\}$ respectively. Moreover, two two‐layers GCN decoders $D_{d}^{m}$ are employed to reconstruct the features for two modalities from these latent embeddings. Mathematically, the reconstruction process is expressed as follows:

(1) $$\left\{\begin{array}{c} Z_{s}^{m}=E_{d}^{m}\left(X^{m},{\tilde{A}}_{s}\right)\\ \left[0.5em\right]{\hat{X}}^{m}=D_{d}^{m}\left(Z_{m}^{s},{\tilde{A}}_{s}\right), \end{array}\right.$$

where $X^{m}$ and ${\hat{X}}^{m}$ represent the original expression matrix and reconstructed expression matrix of modality m. ${\tilde{A}}_{s}=D_{s}^{-1/2}A_{s}D_{s}^{-1/2}$ is the normalized adjacency matrix, $D_{s}$ is the degree matrix of $A_{s}$.

The reconstruction loss $L_{\mathrm{rec}1}$ is computed to measure the discrepancy between the original input features $X^{m}$ and the reconstructed features ${\hat{X}}^{m}$:

(2) $$L_{\mathrm{rec}1}=\sum_{m=1}^{2}{∥X^{m}-{\hat{X}}^{m}∥}_{F}^{2}$$

It should be noted that both RNA and ATAC modalities exhibit inherent sparsity and prevalent dropout events, which arise from technical and biological factors [66]. To address these challenges, we introduced modality‐specific decoders to account for the distinct properties of each omic. The RNA data was modeled by a Zero Inflated Negative Binomial (ZINB) [67] distribution. Specifically, the ZINB decoder contains three separate fully connected layers positioned after two‐layers GCN, which was designed to estimate the three key parameters of the ZINB distribution, including the dropout rate $\pi^{rna}$, the dispersion parameter $\theta^{rna}$, and the mean $\mu^{rna}$:

(3) $$\begin{array}{cc} & \pi^{rna}=\text{Sigmoid}\left(W_{\pi }^{rna}{\hat{X}}^{rna}\right),\;\theta^{rna}=\mathrm{Softplus}\left(W_{\theta }^{rna}{\hat{X}}^{rna}\right),\;\\ & \mu^{rna}=\mathrm{Exp}\left(W_{\mu }^{rna}{\hat{X}}^{rna}\right) \end{array}$$

where ${\hat{X}}^{rna}$ represents the reconstructed gene expression matrix generated by Equation (1), $W_{\pi }^{rna}$, $W_{\theta }^{rna}$, and $W_{\mu }^{rna}$ are learnable weight matrices of fully connected layers. Sigmoid(), Softplus() and Exp() denote the sigmoid, softplus, and exponential functions separately. Then the expression of the j‐th gene on the i‐th spot satisfies the ZINB distribution: ${\hat{X}}_{i,j}^{rna}∼\mathrm{ZINB}\left({\hat{X}}_{i,j}^{rna}|\mu_{i,j}^{rna},\theta_{i,j}^{rna},\pi_{i,j}^{rna}\right)=\pi_{i,j}^{rna}\cdot \delta \left({\hat{X}}_{i,j}^{rna}\right)+\left(1-\pi_{i,j}^{rna}\right)\cdot \mathrm{NB}\left({\hat{X}}_{i,j}^{rna}|\mu_{i,j}^{rna},\theta_{i,j}^{rna}\right)$, where δ() is the Dirac delta function, and $\mathrm{NB}({\hat{X}}_{i,j}^{rna}|\mu_{i,j}^{rna},\theta_{i,j}^{rna})$ is the Negative Binomial (NB) distribution defined as: $\mathrm{NB}\left({\hat{X}}_{i,j}^{rna}|\mu_{i,j}^{rna},\theta_{i,j}^{rna}\right)=\frac{\Gamma ({\hat{X}}_{i,j}^{rna}+\theta_{i,j}^{rna})}{\Gamma \left({\hat{X}}_{i,j}^{rna}+1\right)\cdot \Gamma \left(\theta_{i,j}^{rna}\right)}\cdot {\left(\frac{\theta_{i,j}^{rna}}{\theta_{i,j}^{rna}+\mu_{i,j}^{rna}}\right)}^{\theta_{i,j}^{rna}}\cdot {\left(\frac{\mu_{i,j}^{rna}}{\theta_{i,j}^{rna}+\mu_{i,j}^{rna}}\right)}^{{\hat{X}}_{i,j}^{rna}}$, where Γ() is the Gamma function. Therefore, the reconstruction loss for the RNA modality is employed as:

(4) $$\begin{array}{cc} L_{\mathrm{rec}1}^{rna} & =\alpha \cdot L_{\mathrm{rec}1}+\beta \cdot L_{\text{ZINB}}\text{, with}\;L_{\text{ZINB}}\\ & =\sum_{i,j}-\log\left(\mathrm{ZINB}\left({\hat{X}}_{i,j}^{rna}|\mu_{i,j}^{rna},\theta_{i,j}^{rna},\pi_{i,j}^{rna}\right)\right) \end{array}$$

where the coefficients α=2 and β=0.15 are adopted in the experiment.

Similarly, the ATAC modality is modeled by NB distribution according to previous research [68], The parameters $\theta^{atac}$ and $\mu^{atac}$ are estimated via:

(5) $$\;\theta^{atac}=\mathrm{Softplus}\left(W_{\theta }^{atac}{\hat{X}}^{atac}\right),\;\mu^{atac}=\mathrm{Exp}\left(W_{\mu }^{atac}{\hat{X}}^{atac}\right)$$

The expression of the j‐th peak on the i‐th spot follows the NB distribution: ${\hat{X}}_{i,j}^{atac}∼\mathrm{NB}\left({\hat{X}}_{i,j}^{atac}|\mu_{i,j}^{atac},\theta_{i,j}^{atac}\right)=\frac{\Gamma ({\hat{X}}_{i,j}^{atac}+\theta_{i,j}^{atac})}{\Gamma \left({\hat{X}}_{i,j}^{atac}+1\right)\cdot \Gamma \left(\theta_{i,j}^{atac}\right)}\cdot {\left(\frac{\theta_{i,j}^{atac}}{\theta_{i,j}^{atac}+\mu_{i,j}^{atac}}\right)}^{\theta_{i,j}^{atac}}\cdot {\left(\frac{\mu_{i,j}^{atac}}{\theta_{i,j}^{atac}+\mu_{i,j}^{atac}}\right)}^{{\hat{X}}_{i,j}^{atac}}$. For the ATAC modality, the reconstruction loss is defined as:

(6) $$\begin{array}{cc} L_{\mathrm{rec}1}^{atac} & =\alpha \cdot L_{\mathrm{rec}1}+\beta \cdot L_{\text{NB}}\text{, with}\;L_{\text{NB}}\\ & =\sum_{i,j}-\log\left(\mathrm{NB}\left({\hat{X}}_{i,j}^{atac}|\mu_{i,j}^{atac},\theta_{i,j}^{atac}\right)\right) \end{array}$$

where α=2 and β=0.15. By optimizing the modality‐specific reconstruction loss, we derive the low‐dimensional embeddings $Z_{s}^{1}$ and $Z_{s}^{2}$ for the two respective modalities.

**Training process of conditional diffusion model**. A conditional diffusion model [65, 69] is designed to leverage the embeddings of the higher‐quality modality to guide the refinement of the lower‐quality modality, ensuring a more accurate and biologically meaningful generation. For concise illustration, we denote the low‐dimensional embeddings of the lower‐quality and higher‐quality modalities as $Z_{s}^{1}$ and $Z_{s}^{2}$, respectively. The diffusion model involves two main processes: the forward and reverse processes. In the forward process, $Z_{s}^{1}$ is initialized as the starting point $Z_{0}$, and it is gradually transferred to pure Gaussian noise $Z_{T}$ based on the conditional probability $q\left(Z_{t}|Z_{0}\right)=N\left(Z_{t};\sqrt{{\bar{\alpha }}_{t}}Z_{0},\left(1-{\bar{\alpha }}_{t}\right)I\right)$. By applying the reparameterization trick, we can derive the noisy features $Z_{t}$ at the t‐th step:

(7) $$Z_{t}=\sqrt{{\bar{\alpha }}_{t}}Z_{0}+\sqrt{\left(1-{\bar{\alpha }}_{t}\right)}\varepsilon_{t},\varepsilon_{t}∼N\left(0,I\right)$$

here, ${\bar{\alpha }}_{t}=\prod_{i=1}^{T}\alpha_{i}$, and a cosine noise scheduling mechanism is employed to enable smooth noise distribution, that is:

(8) $$\alpha_{t}=\cos^{2}\left(\frac{t/T+s}{1+s}\cdot \frac{\pi }{2}\right)$$

where the hyper‐parameter s∈(0,1) is used to control the noise level at each iteration. As shown in Figure 1, the reverse process began from the input embeddings constructed by concatenating the two modalities ${\tilde{Z}}_{t}=\left[Z_{t},Z_{s}^{2}\right]$, providing the denoising network with a more comprehensive representation of the data, ensuring the conditional diffusion model can leverage the complementary information from two modalities as the condition during the reverse process. Importantly, the predicted noise corresponding to $Z_{s}^{2}$ was masked out and did not contribute to the loss, therefore, $Z_{s}^{2}$ served solely as a conditional guidance signal rather than a denoising target. Besides, the embeddings of the higher‐quality modality $Z_{s}^{2}$ are processed by a Multi‐Layer Perceptron (MLP) to obtain the representations $Z_{c}^{2}=\mathrm{MLP}\left(Z_{s}^{2}\right)$, which are employed as condition to denoise the embeddings of the lower‐quality modality through a learnable DiT based conditional denoising model. Diffusion Transformer (DiT) [29] utilized transformers as the backbone of diffusion models, achieving superior performance over traditional U‐Net‐based approaches. In implementation, spatial dependencies were encoded through learnable spatial embeddings obtained from a pretrained graph autoencoder, which were incorporated into the input features of DiT. The multi‐head self‐attention mechanism in DiT subsequently enabled the model to jointly attend to molecular and spatial information, thereby facilitating the learning of both local and global spatial relationships, while simultaneously enhancing the understanding of cross‐modality interactions. Specifically, the noisy features ${\tilde{Z}}_{t}$ are progressively restored by the following:

(9) $$p_{\theta }\left(Z_{t-1}|{\tilde{Z}}_{t},Z_{c}^{2}\right)=N\left(\mu_{\theta }\left({\tilde{Z}}_{t},Z_{c}^{2}\right),\Sigma_{\theta }\left({\tilde{Z}}_{t},Z_{c}^{2}\right)\right)$$

where N represents a Gaussian distribution parameterized by the mean $\mu_{\theta }\left({\tilde{Z}}_{t},Z_{c}^{2}\right)=\frac{1}{\sqrt{\alpha_{t}}}\left({\tilde{Z}}_{t}-\frac{1-\alpha_{t}}{\sqrt{1-{\bar{\alpha }}_{t}}}\varepsilon_{\theta }\left({\tilde{Z}}_{t},Z_{c}^{2}\right)\right)$ and variance $\Sigma_{\theta }\left({\tilde{Z}}_{t},Z_{c}^{2}\right)=\frac{\left(1-\alpha_{t}\right)\left(1-{\bar{\alpha }}_{t-1}\right)}{1-{\bar{\alpha }}_{t}}$, the neural network is employed to predict the statistics of $p_{\theta }$. By reparameterizing $\mu_{\theta }$ as a noise prediction network $\varepsilon_{\theta }$, the denoising model could be optimized using the mean squared error between the predicted noise $\varepsilon_{\theta }\left(Z_{t},Z_{c}^{2}\right)$ and the ground truth Gaussian noise $\varepsilon_{t}$:

(10) $$L_{\text{diff}}=E_{t∼\left[1,T\right],Z_{t},\varepsilon_{t}}{∥\left(\varepsilon_{t}-\varepsilon_{\theta }\left({\tilde{Z}}_{t},Z_{c}^{2}\right)\right)∥}_{2}^{2}$$

**Inference process of conditional diffusion model**. Once the DiT‐based conditional diffusion model was trained, the denoised embeddings $Z_{0}^{*}$ of the lower‐quality modality could be enhanced by the higher‐quality modality through the learned cross‐modality relationships. Concretely, a random initial value $Z_{T}^{*}∼N\left(0,I\right)$ is sampled and the inference process at each time step t is performed by using trained DiT based conditional denoising model (Equation (9)), i.e., $Z_{t-1}^{*}∼p_{\theta }\left(Z_{t-1}^{*}|{\tilde{Z}}_{t}^{*},Z_{c}^{2}\right)$, where ${\tilde{Z}}_{t}^{*}=\left[Z_{t}^{*},Z_{s}^{2}\right]$. Finally, the embeddings $Z_{0}^{*}$ of the lower‐quality modality could be iteratively recovered after T steps, and its corresponding expression matrix could be reconstructed through the pre‐trained decoder according to Equation (1), as formulated by ${\hat{X}}^{1^{*}}=D_{d}^{1}\left(Z_{0}^{*},{\tilde{A}}_{s}\right)$.

#### 4.1.2 Integration Phase of CANDIES

After the denoising phase, the quality of spatial multi‐omics data was significantly enhanced. In the integration phase, a unified representation was obtained by leveraging both the spatial information and expression data from multiple modalities. Specifically, graph convolutional networks (GCNs) were employed on spatial graph or feature graph to extract spatial or feature embeddings for every spot within each modality. Then, contrastive learning was utilized to effectively align heterogeneous cross‐modal spatial and feature information. Finally, an attention mechanism was exploited to generate the fused representations by integrating spatial and feature information.

**Construct feature neighbor graph and encode features**. In a complex tissue sample, cells having the same cell types/states might not be in spatial adjacency with one another; in fact, they could be located quite far apart. To preserve both spatial adjacent information and the unique characteristics of non‐adjacent cells with similar cell types/states, we propose to construct both the spatial graph $G_{s}^{m}=\left(V,E_{s},A_{s},X^{m}\right)$ and the feature graph $G_{f}^{m}=\left(V,E_{f},A_{f}^{m},X^{m}\right)$ for the m∈{1,2}‐th modality. For lower‐quality modality 1, the enhanced profiles ${\hat{X}}^{1^{*}}$ generated by denoising phase were adopted as feature matrix $X^{1}$. Besides, by applying KNN algorithm, spatial adjacency matrix $A_{s}\in R^{N\times N}$ is calculated according to the spatial coordinates $S={\left\{\left(x_{i},y_{i}\right)\right\}}_{i=1}^{N}$, and the adjacency matrix of the feature graph $A_{f}^{m}\in R^{N\times N}$ is computed according to the cosine similarity of expression feature matrix $X^{m}$. By default, k set as 3 and 20 for spatial graph and feature graph.

Next, for each modality, we employed a parameter‐sharing GCN to extract information separately from the spatial graph and the feature graph. By applying a shared‐parameter GCN, we ensured that spatial and feature‐based embeddings were aligned in a common representation space, which facilitates better integration of multi‐modal information. The spatial graph captured local tissue organization by encoding physical spot proximity, whereas the feature graph preserves molecular similarity, linking spots that share similar expression profiles despite being spatially distant. This dual‐graph structure allowed CANDIES to effectively learn both spatially and phenotypically relevant patterns, ensuring that biologically meaningful relationships were retained. Specifically, the representations of cells were updated by iteratively aggregating information from their neighboring nodes in each graph, the cell's embeddings in the l‐th layer for the spatial graph $G_{s}^{m}$ and the feature graph $G_{f}^{m}$ can be formalized as:

(11) $$\begin{array}{ccc} H_{s,l}^{m} & = & \sigma \left({\tilde{A}}_{s}H_{s,l-1}^{m}W_{s,l}^{m}\right) \end{array}$$

(12) $$\begin{array}{ccc} H_{f,l}^{m} & = & \sigma \left({\tilde{A}}_{f}^{m}H_{f,l-1}^{m}W_{f,l}^{m}\right) \end{array}$$

where the initial feature vectors of two graphs for N spots in GCN are both $X^{m}$, i.e., $H_{s,0}^{m}=H_{f,0}^{m}=X^{m}$. ${\tilde{A}}_{s}={D_{s}}^{-\frac{1}{2}}\left(A_{s}+I\right){D_{s}}^{-\frac{1}{2}}$ is the symmetrically normalized spatial adjacency matrix, ${\tilde{A}}_{f}^{m}={D_{f}^{m}}^{-\frac{1}{2}}\left(A_{f}^{m}+I\right){D_{f}^{m}}^{-\frac{1}{2}}$ is the normalized feature similarity matrix. $W_{s,l}^{m},W_{f,l}^{m}\in R^{D\times F}$ are the shared weight matrices in the l‐th layer of GCN. σ denotes the ReLU activation function. After L layers, the final spatial and feature embedding outputs of GCN in Equation (11) are $H_{s,L}^{m}$ and $H_{f,L}^{m}$.

**Contrastive loss for cross‐modality alignment**. Contrastive learning [70] was a self‐supervised learning technique that aims to learn useful representations by maximizing the similarity between positive pairs (similar samples) and minimizing the similarity between negative pairs (dissimilar samples). In spatial multi‐omics data, each spot comprises two modalities, and m‐th modality had spatial and feature embeddings (i.e., $H_{s,L}^{m}$ and $H_{f,L}^{m}$) calculated by Equation (11). In order to obtain effective integrated spot embeddings, a contrastive loss was employed to pull the spatial or feature embeddings of the same spot across the two modalities closer while pushing apart that of different spots. Importantly, the selection of positive and negative samples followed a standard practice [70]. Manually selecting neighboring spots as positive samples or randomly choosing spots as negative samples might introduce bias due to spatial heterogeneity and uneven cell‐type composition. Therefore, we treated all other spots as negative samples, avoiding such subjective bias and learned a more robust and unbiased cross‐modal alignment. By applying contrastive learning, CANDIES learned the shared latent space that is invariant to the modality, allowing better alignment among different modalities. For spatial embeddings $H_{s,L}^{m}$, the formulation is:

(13) $$L_{s}\left(H_{s,L}^{1},H_{s,L}^{2}\right)=-\sum_{i=1}^{N}\log\frac{e^{\frac{\text{sim}(H_{s,L}^{1}\left(i,:\right),H_{s,L}^{2}\left(i,:\right))}{\tau }}}{\sum_{j=1,j\neq i}^{N}e^{\frac{\text{sim}(H_{s,L}^{1}\left(i,:\right),H_{s,L}^{2}\left(j,:\right))}{\tau }}}$$

where sim(,) represents the similarity measurement function, τ is a temperature parameter, and $H_{s,L}^{1}\left(i,:\right)$ denotes the spatial embedding of the i‐th cell in the 1st modality. Similarly, for feature embeddings $H_{f,L}^{m}$, we can also obtain the formulation:

(14) $$L_{f}\left(H_{f,L}^{1},H_{f,L}^{2}\right)=-\sum_{i=1}^{N}\log\frac{e^{\frac{\text{sim}(H_{f,L}^{1}\left(i,:\right),H_{f,L}^{2}\left(i,:\right))}{\tau }}}{\sum_{j=1,j\neq i}^{N}e^{\frac{\text{sim}(H_{f,L}^{1}\left(i,:\right),H_{f,L}^{2}\left(j,:\right))}{\tau }}}$$

The overall contrastive loss is:

(15) $$L_{cl}=L_{s}\left(H_{s,L}^{1},H_{s,L}^{2}\right)+L_{f}\left(H_{f,L}^{1},H_{f,L}^{2}\right)$$

**Integrating spatial and feature embeddings by attention**. To further integrate the information from both modalities, we computed the mean of the spatial embeddings and feature embeddings across modalities for each spot. Specifically, the aggregated spatial embeddings $H_{s}$ and feature embeddings $H_{f}$ are derived as follows:

(16) $$H_{s}=\frac{1}{2}\left(H_{s,L}^{1}+H_{s,L}^{2}\right),\;H_{f}=\frac{1}{2}\left(H_{f,L}^{1}+H_{f,L}^{2}\right)$$

These aggregated representations capture shared biological structures and spatial relationships across different modalities. Following this, to effectively combine the aggregated spatial embeddings $H_{s}$ and feature embeddings $H_{f}$ into a unified representation H, we introduced a learnable attention mechanism that dynamically adjusts the contributions of two types of embeddings based on their relative importance for each cell:

(17) $$\begin{array}{ccc} v_{s}^{i} & = & u^{T}\cdot \sigma \left(W_{s}{\left[H_{s}\left(i,:\right)\right]}^{T}+b_{s}^{i}\right) \end{array}$$

(18) $$\begin{array}{ccc} v_{f}^{i} & = & u^{T}\cdot \sigma \left(W_{f}{\left[H_{f}\left(i,:\right)\right]}^{T}+b_{f}^{i}\right)\alpha_{s}^{i}\\ & = & \frac{\exp(v_{s}^{i}+\eta )}{\exp\left(v_{s}^{i}+\eta \right)+\exp\left(v_{f}^{i}+\eta \right)},\alpha_{f}^{i}=\frac{\exp(v_{f}^{i}+\eta )}{\exp\left(v_{s}^{i}+\eta \right)+\exp\left(v_{f}^{i}+\eta \right)}\\ & & \times \;H\left(i,:\right)=\alpha_{s}^{i}H_{s}\left(i,:\right)+\alpha_{f}^{i}H_{f}\left(i,:\right) \end{array}$$

where $W_{s},W_{f}$, $b_{s}^{i},b_{f}^{i}$, and u are trainable parameters, and $\eta =1\times 10^{-6}$. $v_{s}^{i}$ and $v_{f}^{i}$ are the attention coefficient, while $\alpha_{s}^{i}$ and $\alpha_{f}^{i}$ are the normalized attention scores, representing the importance of spatial or feature information to the representation of cell i.

**Feature reconstruction loss**. To ensure the fused representation H retains biologically meaningful information from spatial multi‐omics, we introduce a feature reconstruction function $f_{\text{rec}}^{m}$ for each modality, which maps the fused representation back to the original feature space:

(19) $$\begin{array}{ccc} {\hat{X}}_{s}^{m} & = & f_{\text{rec}}^{m}\left(H,{\tilde{A}}_{s}\right) \end{array}$$

(20) $$\begin{array}{ccc} {\hat{X}}_{f}^{m} & = & f_{\text{rec}}^{m}\left(H,{\tilde{A}}_{f}^{m}\right) \end{array}$$

where ${\hat{X}}_{s}^{m},{\hat{X}}_{f}^{m}$ are the reconstructed feature of modality m using GCN on spatial graph and feature graph. The reconstruction loss was then defined as the mean squared error (MSE) between the original input features $X^{m}$ and the reconstructed features:

(21) $$L_{\text{rec2}}=\sum_{m=1}^{2}(∥X^{m}-{\hat{X}}_{s}^{m}{∥}_{F}^{2}+∥X^{m}-{\hat{X}}_{f}^{m}{∥}_{F}^{2})$$

By incorporating the reconstruction loss, CANDIES maintains fidelity to the raw data while leveraging cross‐attention to integrate spatial and feature information.

**Integration model training** Combining the contrastive loss $L_{cl}$ in Equation (15) and feature reconstruction loss $L_{\text{rec2}}$ in Equation (21), the overall loss of integration phase in CANDIES is as follows:

(22) $$L_{int}=\lambda_{cl}L_{cl}+\lambda_{rec}L_{rec2}$$

where $\lambda_{cl}=\lambda_{rec}=0.5$ are adopted in the experiment. The final fused representation H by optimization Equation (22) can be used in various downstream analysis, including domain identification, pseudo‐spatiotemporal map construction, trait‐domain association map generation.

#### 4.1.3 Lower‐Quality Modality Determination in Denoising Phase

In the denoising phase, CANDIES aims to leverage the higher‐quality modality to refine the lower‐quality modality. Therefore, determining the relative quality between modalities was a crucial step. To achieve this, we established the following modality quality evaluation principles. During the denoising phase, we first pre‐trained a dual graph auto‐encoder to obtain low‐dimensional embeddings for all spots across both modalities. We applied the Leiden algorithm on these embeddings to derive clustering results for each modality. Based on the clustering outcomes, we identified the lower‐quality modality using four well‐established unsupervised metrics: the Silhouette Index (SI), DBI, Moran's I, and the CHI. A modality with a lower DBI or higher SI, Moran's I, and CHI was considered to have higher quality.

Furthermore, we tested several clustering methods, including k‐means, Leiden, and mclust on both simulated dataset and the human skin dataset. The corresponding results were shown in Tables S5–S6. It was apparent that the final selection of the lower‐quality modality was consistent across all three clustering methods. This consistency indicated that our selection strategy of the lower‐quality modality was robust and not dependent on a particular method.

#### 4.1.4 CANDIES Training Details

As CANDIES follows a two‐stage framework, we detailed the training process for each phase. In the denoising phase, the pre‐trained graph auto‐encoder was trained for 400 epochs, with the latent dimensionality fixed as 64 across all datasets. In the conditional diffusion model, CANDIES utilized AdamW optimizer with a default learning rate of 0.001. To maintain consistency with traditional diffusion models, we partitioned the datasets into k folds based on the number of spots, where k was set as three by default. The conditional diffusion model was trained for a maximum of 1,000 epochs, with the number of diffusion steps as 800. In the integration phase, the optimizer was Adam, also with a default learning rate of 0.001. The maximum number of total training epochs in this phase was set as 300.

### 4.2 Downstream Analysis

#### 4.2.1 Pseudo‐Spatiotemporal Map Generation

To construct the pseudo‐spatiotemporal map (pSM) of the tissue, we calculated a pseudo‐spatiotemporal value for each spot (or cell) using the *scanpy.tl.dpt* function implemented in the Scanpy package (v.1.10.3) [71]. Conceptually inspired by pseudotime analysis in single cell omics, the pSM extends this idea to a spatial context, aiming to model the progression of cellular states across the tissue. The root of the trajectory was manually defined as a biologically relevant starting region that represents the initial stage of a dynamic process. Specifically, the dermis region, diencephalon_and_hindbrain region, and forebrain region were selected as the root spots in the spatial CITE‐seq human skin data, MISAR‐seq E18.5 mouse brain data, and spatial ATAC–RNA‐seq mouse embryo data, respectively. The inferred pseudo‐spatiotemporal ordering captures how cells evolve spatially under the assumption that spatially localized cells may represent distinct stages of biological progression, such as development or tissue adaptation. For visualization, the pSM employed a color gradient from blue to red to denote early‐to‐late stages along the inferred trajectory, thereby revealing the spatial dynamics of cellular state transitions within the tissue.

#### 4.2.2 Pseudo‐Time Associated Genes

For each gene, we calculated the pearson correlation coefficient between its expression across cells and the pseudo‐spatiotemporal map (pSM) values derived from CANDIES. Genes exhibiting a significant correlation with a threshold of P ≤ $1\times 10^{-10}$ were identified as pseudo‐time‐associated genes.

#### 4.2.3 Cluster‐Specific Differentially Expressed Genes (DEGs)

For each cluster, we performed differential expressed genes analysis using the *scanpy.tl.rank_genes_groups* function from the scanpy package (v.1.10.3), and chose the Wilcoxon rank‐sum test as the statistical method. A gene was classified as differentially expressed if its adjusted p‐value (Q‐value) was below the significance threshold of 0.05. To obtain the overlapped DEGs, we first identified cluster‐specific DEGs independently for each modality. For each cluster, the overlapped DEGs were then defined as the intersection of DEGs across the modalities.

#### 4.2.4 GO Enrichment Analysis

GO (Gene Oncology) enrichment analysis were performed on pseudo‐time‐associated genes and cluster‐specific DEGs using the *gseapy.enrichr* function implemented in the gseapy package (v.1.1.6) [72]. The analysis was conducted with the *GO_Biological_Process_2021* database as the reference, enabling the identification of enriched biological processes associated with the identified gene sets.

#### 4.2.5 Spatially Resolved Trait Association Mapping

We carried out gsMap analysis using the CANDIES embeddings as input to identify trait–spot associations across three tissues, including the mouse brain, mouse embryo, and human lymph. In the gsMap analysis, we directly computed cosine similarity and GSS using the latent embeddings from CANDIES. gsMap assigned the GSS of each spot as annotations to SNPs within a window extending 50 kb upstream and 50 kb downstream of each gene's transcribed region, along with SNP‐to‐gene maps established using epigenomic data [64]. Within the S‐LDSC framework, we considered stratified LD scores with respect to both baseline annotations and GSS annotations, thus obtaining the GSS enrichment p‐value conditional on the baseline annotations. The baseline annotations include: (1) a constant value of 1 for all SNPs; (2) binary values (0 or 1) to indicate whether SNPs were mapped to genes; and (3) binary values to indicate whether SNPs were mapped to other functional annotations, including coding, conserved, and regulatory regions (e.g., promoter, enhancer, histone marks). Finally, to quantify the significance of the association of a specific spatial region with a trait, gsMap utilized the Cauchy combination test to aggregate the p‐values of individual spots within that spatial region.

### 4.3 Data Description and Preprocessing

To comprehensively evaluate the performance of CANDIES, we conducted extensive quantitative and qualitative experiments across a simulated spatial multi‐omics dataset, and six real‐word spatially resolved multi‐omics datasets.

#### 4.3.1 Simulated Data

To evaluate the spatial multi‐omics data analysis methods, we first followed SpatialGlue [25] to generate paired spatial multi‐omics datasets comprising two modalities (RNA and protein). Within it, the ‘ggblocks’ model [73] was employed to produce the spatial pattern with four different factors of 1296 spots (or cells). For the RNA modality, the gene expression matrix $X^{rna}\in R^{1296\times 800}$ was generated from a Zero Inflated Negative Binomial (ZINB) distribution formulated as:

(23) $$\begin{array}{cc} X_{i,j}^{rna} & ∼\mathrm{ZINB}(X_{i,j}^{rna}|M_{i,j}^{rna},\theta_{i,j}^{rna},\pi_{i,j}^{rna})\\ & =\pi_{i,j}^{rna}\cdot \delta \left(X_{i,j}^{rna}\right)+\left(1-\pi_{i,j}^{rna}\right)\cdot \mathrm{NB}\left(X_{i,j}^{rna}|M_{i,j}^{rna},\theta_{i,j}^{rna}\right) \end{array}$$

where δ(·) is the Dirac delta function representing zero‐inflated dropout events, $\pi_{i,j}^{rna}$ is the dropout probability, $M_{i,j}^{rna}$ represents the mean expression level, $\theta_{i,j}^{rna}$ is the dispersion (shape) parameter, and NB denotes Negative Binomial (NB) distribution. For the protein modality, the expression matrix $X^{pro}\in R^{1296\times 100}$ was generated from a NB distribution:

(24) $$X_{i,j}^{pro}∼\mathrm{NB}\left(X_{i,j}^{pro}|M_{i,j}^{pro},\theta_{i,j}^{pro}\right)$$

To further simulate modality‐specific quality differences, we introduced controlled noise into the mean terms of Equations (23) and (24). Specifically, for the RNA modality, the mean in Equation (23) is defined as: $M^{rna}=B^{rna}+F^{rna}W^{rna}+\varepsilon^{rna}$, where $\varepsilon^{rna}$ represents noise sampled from Gaussian distribution $\varepsilon^{rna}∼N\left(2,\sigma^{2}\right)$ with σ ranging from 2 to 5 (in increments of 0.5), and the value of every element in $B^{rna}$ was 0.5, representing the mean value of background expression. $F^{rna}\in R^{1296\times 4}$ was a binary matrix indicating the activity of spatial factors at each spot, $F_{i,l}^{rna}=1$ if the spatial factor l was active at spatial spot i, otherwise $F_{i,l}^{rna}=0$. $W^{rna}\in R^{4\times 800}$ was the mean expression matrix for spatial factors. The dropout rate $\pi_{i,j}^{rna}$ was fixed at π, and the dispersion parameter $\theta_{i,j}^{rna}$ was set to 3. For the protein modality, the mean in Equation (24) is defined as: $M^{pro}=B^{pro}+F^{pro}W^{pro}+\varepsilon^{pro}$, where each element in $B^{pro}$ was 2, and $F^{pro}\in R^{1296\times 4}$ and $W^{pro}\in R^{4\times 100}$ were defined analogously to the RNA modality. Gaussian noise $\varepsilon^{pro}∼N\left(2,1\right)$ with a mean of 2 and a standard deviation of 1 was applied to ensure that the protein modality maintained higher data quality than the RNA modality. In order to simulate varying data sparsity, the dropout rate $\pi_{i,j}^{rna}$ in Equation (23) varied from 0.1 to 0.4 (step size 0.1). The detailed parameters are shown in the Tables S3 and S4. This simulation design ensures that the generated data exhibit realistic sparsity, modality heterogeneity, and biological variability.

#### 4.3.2 MISAR‐seq E15.5 and E18.5 Mouse Brain Dataset

MISAR‐seq enabled the simultaneous profiling of chromatin accessibility and gene expression during mouse brain development [7]. E15.5 mouse brain dataset comprised 1,949 spots, 32,285 genes, and a peak count ranging up to 191,034. E18.5 mouse brain dataset comprises 2,129 spots, 32,285 genes, and a peak count ranging up to 161,461.

Both datasets underwent identical preprocessing steps. For the RNA modality, we removed genes expressed in fewer than 20 spots and selected spots that contain more than 100 expressed genes. Then, we normalized the gene expression count followed by a log transformation. Finally, we identified the top 4,000 HVGs by scanpy package (v.1.10.3) [71]. For the ATAC modality, as the dimensions were much higher than that of the RNA modality, we applied Latent Semantic Indexing (LSI) to select informative features [74, 75]. Specifically, we first used TF‐IDF (Term Frequency‐Inverse Document Frequency) normalization, and the normalized data were log‐transformed and further processed with randomized Singular Value Decomposition (SVD) to reduce dimensionality and capture the most meaningful features in the count data.

#### 4.3.3 Spatial CITE‐seq Human Skin Dataset

Spatial CITE‐seq was employed to map early immune cell activation within a skin biopsy tissue collected from the injection site of a Coronavirus Disease 2019 (COVID‐19) mRNA vaccine, co‐profiling spatially resolved proteome and transcriptome [3]. Unlike previous study, which analyzed a much smaller set of proteins and struggled with tissue region clustering based solely on protein profiles, spatial CITE‐seq employs a cocktail of approximately 200–300 antibody‐derived tags (ADTs) to stain a tissue slide. This dataset comprises 15,486 genes and 283 proteins on 1691 spots.

For the RNA modality, we first removed genes expressed in fewer than ten spots to eliminate low‐quality features. Subsequently, we normalized the gene expression count using the *scanpy.pp.normalize_total* function, followed by a log transformation and standardization to ensure comparability across cells. Finally, we identified the top 3000 highly variable genes (HVGs) using the Seurat_v3 [76] implemented in scanpy package (v.1.10.3) [71]. For the protein modality, we employed the centered log‐ratio (CLR) transformation to normalize the count data for each spot [77]. Following CLR transformation, we standardized the data using the *scanpy.pp.scale* function.

#### 4.3.4 Spatial‐Mux‐seq E13 Mouse Embryo Dataset

Spatial‐Mux‐seq enabled simultaneous profiling of five different modalities: two histone modifications, chromatin accessibility, whole transcriptome, and a panel of proteins in a spatially resolved manner [17]. In experiments conducted on E13 sagittal mouse embryo sections at 50‐μm resolution, spatial‐Mux‐seq targeted two histone modifications: H3K27me3 and H3K27ac, the dataset comprises 2,102 spots and 24,333 genes for each modality.

For the two modalities, we removed genes expressed in fewer than 20 spots, then normalized and log‐transformed the gene expression count. Finally, we identified the top 3,000 HVGs using the scanpy package (v.1.10.3).

#### 4.3.5 Spatial ATAC–RNA‐seq E13 Mouse Embryo Dataset

Spatial ATAC‐RNA‐seq enabled spatially resolved, genome‐wide co‐mapping of the epigenome and transcriptome by simultaneously profiling chromatin accessibility and messenger RNA expression [6]. In experiments conducted on embryonic day 13 (E13) mouse embryos, with a pixel size of 50 μm, the dataset comprises 2,186 spots, 20,900 genes, and a peak count reaching up to 87,173, offering a detailed multi‐omics view of embryonic development.

For both the RNA and ATAC modality, we performed preprocessing steps similar as the MISAR‐seq E13 mouse brain dataset.

#### 4.3.6 10x Visium Human Lymph Node Dataset

The human lymph node dataset was generated using 10x Genomics Visium spatial RNA‐protein co‐profiling technology. This spatially resolved multi‐omics dataset originates from a comprehensive study focused on multi‐modal representation learning, capturing both transcriptomic and proteomic profiles within their native tissue architecture. The dataset contains 3,484 spots, 18,085 genes, and 31 proteins.

For the RNA modality, we first filtered out low‐quality features by removing genes expressed in fewer than 20 spots. The remaining gene expression counts were then normalized and followed by a log transformation. To enable cross‐sample comparisons, we standardized the transformed data. Finally, we identified the top 3000 highly variable genes (HVGs). For the protein modality, we performed the same preprocessing workflow as the human skin dataset.

### 4.4 Baseline Methods

For the denoising task, nine methods designed for spatial omics data are included for benchmarking:

- GAAEST: https://github.com/tqwang743/GAAEST‐main
- SCAN‐IT: https://github.com/zcang/SCAN‐IT
- SpaGCN: https://github.com/jianhuupenn/SpaGCN
- SEDR: https://github.com/JinmiaoChenLab/SEDR
- SpaceFlow: https://github.com/hongleir/SpaceFlow
- STAGATE: https://github.com/zhanglabtools/STAGATE
- GraphST: https://github.com/JinmiaoChenLab/GraphST
- BANKSY: https://github.com/prabhakarlab/Banksy_py
- DECIPHER: https://github.com/gao‐lab/DECIPHER

For the integration task, seven methods designed for multi‐omics data are included for benchmarking:

- MultiVI, totalVI: https://github.com/scverse/scvi‐tools
- scMDC: https://github.com/xianglin226/scMDC
- SpatialGlue: https://github.com/JinmiaoChenLab/SpatialGlue
- PRESENT: https://github.com/lizhen18THU/PRESENT
- PRAGA: https://github.com/Xubin‐s‐Lab/PRAGA
- COSMOS: https://github.com/Lin‐Xu‐lab/COSMOS

Among these baselines, SEDR is specially designed for spatial transcriptomic data denoising. SpaGCN was performed solely based on spatial transcriptomic data, without incorporating histological image information. The clustering results for DECIPHER were derived from its spatial embeddings. MultiVI and totalVI were implemented via the scvi‐tools package (v.1.3.0). To ensure a fair comparison, we performed the experiments using the default hyperparameters provided in their official tutorials.

### 4.5 Evaluation Metrics

In our experiments, the evaluation of domain identification performance was conducted using a comprehensive set of metrics. For datasets with available ground truth, we employed supervised clustering metrics, including Mutual Information (Mutual_Info), Normalized Mutual Information (NMI), Adjusted Mutual Information (AMI), V‐measure, Homogeneity, Completeness, Adjusted Rand Index (ARI) and Fowlkes‐Mallows Index (FMI). These metrics were designed to quantify the agreement between the predicted clusters and the annotated labels, offering valuable insights into the accuracy and consistency of the clustering results. On the other hand, for datasets without annotated labels, we utilized unsupervised clustering evaluation metrics, such as the SI, DBI, Moran's I, and CHI. These metrics assess clustering quality by measuring intra‐cluster cohesion, inter‐cluster separation, spatial autocorrelation, and variance ratios, enabling a robust evaluation of clustering structures in the absence of annotated data. For the calculation of Moran's I, we employed the esda package (v.2.7.0) [78]. For other metrics, we utilized functions from the scikit‐learn package (v.1.5.2) [79]. The detailed introduction of metrics used in this paper are listed as follows:

**Mutual Information (MI)**: it is a fundamental concept in information theory that measures the amount of information shared between the predicted labels and the true labels, ranging from 0 to positive values. A higher MI value indicates a stronger relationship between the predicted and the ground truth, the formulation is:

(25) $$\mathrm{MI}\left(U,V\right)=\sum_{u\in U}\sum_{v\in V}P\left(u,v\right)\log\frac{P(u,v)}{P(u)P(v)}$$

where U represents the set of ground truth, and V represents the set of predicted labels. P(u) and P(v) denote the marginal probabilities of the ground truth u and the predicted label v, P(u,v) represents the joint probabilities observing u and v simultaneously.

**Normalized Mutual Information (NMI)**: it is a normalized version of MI that adjusts for the bias introduced by different numbers of labels. It ensures that values remain between 0 and 1, where 1 indicates perfect clustering alignment, and 0 implies no correlation between predicted and ground truth labels. The NMI is defined as:

(26) $$\mathrm{NMI}\left(U,V\right)=\frac{\mathrm{MI}(U,V)}{\sqrt{H(U)H(V)}}$$

where $H\left(U\right)=-\sum_{u\in U}P\left(u\right)\log P\left(u\right)$ and $H\left(V\right)=-\sum_{v\in V}P\left(v\right)\log P\left(v\right)$ denote the entropy of the ground truth and predicted labels, respectively, measuring the uncertainty or variability in each label set.

**Adjusted Mutual Information (AMI)**: it is a variant of MI that corrects for randomness by adjusting the expected mutual information under a random clustering assumption. The higher the AMI value, the more similar the two sets of clusters are. AMI is calculated as follows:

(27) $$\mathrm{AMI}\left(U,V\right)=\frac{\mathrm{MI}(U,V)-E[\mathrm{MI}(U,V)]}{\max(H(U),H(V))-E[\mathrm{MI}(U,V)]}$$

where E[MI(U,V)] represents the expected mutual information given a random labels assignment.

**Homogeneity**. Homogeneity measures whether each predicted cluster contains members of only a single ground truth category. The value of homogeneity ranges from 0 to 1, a higher homogeneity score means that each predicted cluster contains primarily samples from a single ground truth category. Homogeneity is defined as:

(28) $$\mathrm{Homogeneity}\left(U,V\right)=1-\frac{H(V|U)}{H(V)}$$

where $H\left(V|U\right)=-\sum_{u\in U}P\left(u\right)\sum_{v\in V}P\left(v|u\right)\log P\left(v|u\right)$ is the conditional entropy of predicted labels V given the true labels U.

**Completeness**. Completeness evaluates whether all members of a ground truth labels are assigned to the same predicted labels, a higher completeness score indicates that samples belonging to the same ground truth class are well grouped into the same predicted labels. Completeness is computed as:

(29) $$\mathrm{Completeness}\left(U,V\right)=1-\frac{H(U|V)}{H(U)}$$

where $H\left(U|V\right)=-\sum_{v\in V}P\left(v\right)\sum_{u\in U}P\left(u|v\right)\log P\left(u|v\right)$ is the conditional entropy of true labels U given the predicted labels V.

**V‐measure**. V‐measure is a clustering evaluation metric that balances homogeneity and completeness to assess clustering quality. V‐measure range from 0 to 1, where 1 indicates perfect clustering and 0 imply poor clustering, defined as:

(30) $$V=\frac{(1+\beta )\cdot \mathrm{Homogeneity}\cdot \mathrm{Completeness}}{\beta \cdot \mathrm{Homogeneity}+\mathrm{Completeness}}$$

where β controls the weighting between homogeneity and completeness, typically set to 1 for an equal balance.

**Adjusted Rand Index (ARI)**: ARI is a clustering similarity measure that corrects the Rand Index (RI) for random chance. It evaluates how well the predicted clusters match the ground truth labels while considering random assignments. The ARI is computed as:

(31) $$\mathrm{ARI}\left(U,V\right)=\frac{\mathrm{RI}(U,V)-E[\mathrm{RI}(U,V)]}{\max[\mathrm{RI}(U,V)]-E[\mathrm{RI}(U,V)]}$$

(32) $$\mathrm{RI}\left(U,V\right)=\frac{TP+TN}{TP+FP+FN+TN}$$

where TP is the number of true positives, TN is the number of true negatives, FP is the number of false positives, and FN is the number of false negatives.

**Fowlkes‐Mallows Index (FMI)**: FMI measures the geometric mean of precision and recall between the true labels and predicted labels. FMI is defined as:

(33) $$\mathrm{FMI}\left(U,V\right)=\sqrt{\frac{TP}{TP+FP}\cdot \frac{TP}{TP+FN}}$$

a higher FMI score indicates better clustering quality, with 1 being ideal and 0 indicating poor clustering performance.

**Silhouette Index (SI)**: SI measures the quality of clustering by evaluating both intra‐labels cohesion and inter‐labels separation. It is defined for each spot i and then averaged over all the samples. SI ranges from ‐1 to 1, where a high value indicates that the spot is well matched to its own labels and poorly matched to neighboring labels. SI is defined as:

(34) $$\mathrm{SI}=\frac{1}{N}\sum_{i=1}^{N}\frac{b(i)-a(i)}{\max(a(i),b(i))}$$

where a(i) is the average distance of sample i to other spots in the same label, and b(i) is the minimum average distance of i to spots in other labels.

**Davies‐Bouldin Index (DBI)**: DBI evaluates clustering quality by measuring the average similarity between each label and its most similar label, based on the ratio of intra‐label dispersion to inter‐label separation. A lower DB score indicates better clustering, and it is defined as:

(35) $$\mathrm{DBI}=\frac{1}{N}\sum_{i=1}^{N}\max_{j\neq i}\frac{s_{i}+s_{j}}{d_{ij}}$$

where $s_{i}$ is the average intra‐label distance for label i, and $d_{ij}$ is the distance between the centroids of labels i and j.

**Moran's I**. Moran's I is a statistical measure used to evaluate spatial autocorrelation, determining whether similar values are clustered, dispersed, or randomly distributed across a spatial setting. It is widely employed in spatial clustering tasks to assess the degree of spatial dependence within the data. It is computed as

(36) $$I=\frac{N}{\sum_{i}\sum_{j}w_{ij}}\frac{\sum_{i}\sum_{j}w_{ij}\left(x_{i}-\bar{x}\right)\left(x_{j}-\bar{x}\right)}{\sum_{i}{\left(x_{i}-\bar{x}\right)}^{2}}$$

where N is the total number of observations, $x_{i}$ and $x_{j}$ are the observed values, $\bar{x}$ is the mean of x, $w_{ij}$ represents the spatial weight between i and j. Moran's I ranges from ‐1 to 1, where values close to 1 indicate positive spatial autocorrelation (clustering of similar values), values close to ‐1 suggest negative spatial autocorrelation (dispersion of dissimilar values), and values near 0 imply a random spatial distribution.

**Calinski‐Harabasz Index (CHI)**: CHI also known as the Variance Ratio Criterion (VRC), evaluates clustering quality defined as the ratio of the between‐cluster separation (B) to the within‐cluster dispersion (W), normalized by their number of freedom degrees. A higher score indicates better‐defined clusters. CHI is given by

(37) $$\mathrm{CHI}=\frac{\mathrm{Tr}(B)}{\mathrm{Tr}(W)}\times \frac{N-k}{k-1}$$

where B (between‐cluster scatter matrix) is the weighted sum of squared Euclidean distances between each cluster centroid and the overall data centroid: $B=\sum_{i=1}^{k}n_{i}{\left|\left|c_{i}-c\right|\right|}^{2}$, $n_{i}$ is the number of samples in cluster $C_{i}$, $c_{i}$ is the centroid of $C_{i}$, and c is the overall centroid of the data. B measures how well the clusters are separated from each other (the higher, the better). W (within‐cluster sum of squares) is the sum of squared Euclidean distances between the data points x and their respective cluster centroids: $W=\sum_{i=1}^{k}\sum_{x\in C_{i}}{\left|\left|x-c_{i}\right|\right|}^{2}$. W measures the compactness or cohesiveness of the clusters (the smaller, the better). Furthermore, Tr() is the trace function, N is the total number of samples, and k is the number of clusters.

### 4.6 Running Time and Memory Usage

As shown in Figure S9, we included a comprehensive comparison of running time and memory usage across multiple datasets with representative baseline methods. In Figure S9a,b, CANDIES exhibited a relatively higher running time compared to several baseline methods, while maintaining a moderate GPU memory across all datasets. In particular, although the overall computational cost was higher, the peak memory usage remained comparable to or lower than that of other baseline methods, indicating that CANDIES is memory‐efficient in practice.

The increased running time was primarily attributed to the diffusion‐based denoising phase. Unlike traditional encoder–decoder architectures, the conditional diffusion model required multiple diffusion steps to progressively refine latent representations. Nevertheless, as demonstrated in the parameter sensitivity analysis, a moderate number of diffusion steps was sufficient to achieve a balance between optimal performance and computational efficiency, which helped mitigate excessive computational overhead. To alleviate this limitation, future work will explore acceleration strategies for diffusion models, such as adopting Denoising Diffusion Implicit Models (DDIM) [80].

### 4.7 Sensitivity Analysis of Hyperparameters

To demonstrate the robustness of CANDIES, we conducted a sensitivity analysis on the key parameters of CANDIES in Figure S8. Specifically, we evaluated the impact of the numbers of spatial neighbors and feature neighbors used in constructing the spatial graph $G_{s}^{m}$ and feature graph $G_{f}^{m}$, the balance coefficients α and β in Equations (4) and (6), the number of diffusion steps T in Equation (10), and the trade‐off weights $\lambda_{cl}$ and $\lambda_{rec}$ between the contrastive loss and the reconstruction loss in Equation (22).

First, We investigated the sensitivity of the weighting parameters α (ranging from 1.0, 1.5, 2.0, 2.5, to 3.0) and β (ranging from 0.05, 0.10, 0.15, 0.20, to 0.30) in Equations (4) and (6). As shown in Figure S8a, the performance reached its optimum when α=2.0, after which further increasing α leaded to slight performance degradation. Therefore, α=2.0 was selected as the default weight in Equation (4) and (6). Moreover, Figure S8b presented the sensitivity analysis with respect to β. Across both denoising and integration phases, the performance improved as β increases from 0.05 to 0.15, indicating that incorporating the modality‐specific distribution term effectively accounts for the characteristics of each modality. Consequently, β was set to 0.15 as all evaluation metrics reach their highest values.

Second, we investigated the sensitivity of the number of diffusion steps T in Equation (10), which was ranged from 300, 500, 800, 1000, to 1200. As shown in Figure S8c, both denoising and integration performance gradually improved as T increases from 300 to 800, indicating that a sufficient number of diffusion steps is essential for effective noise removal and representation learning. The best performance across all evaluation metrics was consistently achieved at T=800, therefore, we set T=800 to achieve optimal clustering performance while maintaining a relatively low computational cost.

Third, we evaluated the impact of initial KNN graphs constructed with different values of k, ranging from 1, 3, 5, 8, to 10 for spatial k and from 10, 15, 20, 25, to 30 for feature k. As shown in Figure S8d, when the neighbors of spatial graph increased from 1 to 3, all evaluation metrics exhibited a clear improvement, indicating that incorporating spatial context is beneficial for clustering performance. Importantly, the performance remained relatively stable for larger spatial k values beyond 3, demonstrating that CANDIES is robust to the choice of spatial neighborhoods once sufficient local spatial information was captured. Therefore, CANDIES set the spatial k=3 in all experiments for a lower computational cost. Figure S8e evaluated the impact of the feature k on integration performance. Owing to the denoising phase of CANDIES, the performance was insensitive to the choice of the neighbors in feature graph, exhibiting consistently stable values across a broad range. Therefore, the feature k was set to 20 as most evaluation metrics reach their highest values.

Finally, We investigated the trade‐off between the contrastive loss and the reconstruction loss by varying the weighting parameters $\lambda_{cl}$ and $\lambda_{rec}$ in Equation (22). Specifically, the ratios $\lambda_{cl}/\lambda_{rec}$ were varied from 0.1/0.9, 0.3/0.7, 0.5/0.5, 0.7/0.3 to 0.9/0.1. As shown in Figure S8f, the integration performance consistently improved as the contributions of the two loss terms become more balanced, reaching its optimum when $\lambda_{cl}=\lambda_{rec}=0.5$. When one loss term dominates the other, the performance degraded slightly, indicating that overemphasizing contrastive learning or data reconstruction alone was insufficient for effective integration. Consequently, we adopted $\lambda_{cl}=\lambda_{rec}=0.5$ in all experiments.

## Author Contributions

Conceptualization: Y.L., W.Z‥ Methodology: Y.L., W.Z‥ Investigation: W.Z., J.W‥ Visualization: W.Z., Y.L., J.W‥ Writing – original draft: Y.L., W.Z., Y.L‥ Writing – review and editing: Y.L., W.Z., Y.L., M.C., H.C‥ Supervision: Y.L., M.C., H.C‥

## Funding

This work was supported in part by the National Key Research and Development Program of China (2025YFE0216700), National Natural Science Foundation of China (U21A20520, 62325204, 62306118, 12501402), Hong Kong Research Grant Council (21305525), Guangdong Basic and Applied Basic Research Foundation (2026A1515010725, 2026A1515010402), Fundamental Research Funds for the Central Universities (2025ZYGXZR054), and City University of Hong Kong (21300423, 7020141) .

## Conflicts of Interest

The authors declare no conflicts of interest.

## Supporting information


**Supporting File**: advs75404‐sup‐0001‐SuppMat.pdf.

## Data Availability

Data sharing is not applicable to this article as no new data were created or analyzed in this study.
