# Supplementary material for: Cross‐Modal Denoising and Integration of Spatial Multi‐Omics Data with CANDIES
Source: Adv Sci (Weinh). 2026 Apr 27;13(40):e23754. doi: 10.1002/advs.202523754 (PMC13335510; doi:10.1002/advs.202523754)
Supplement: Supplementary file 1 — Supporting File: advs75404‐sup‐0001‐SuppMat.pdf. [file ADVS-13-e23754-s001.pdf]

Supplementary Materials for ”Cross-modal Denoising and Integration of Spatial Multi-omics data with CANDIES”

Dataset name	Platform (omics)	High-quality modality	Size (spots × genes/proteins/peaks)
Synthetic data	- (RNA-protein)	protein	1,296 × 800 / 100
E15.5 Mouse Brain	MISAR-seq (RNA-ATAC)	RNA	1,949 × 32,285 / 191,034
Human Skin	Spatial CITE-seq (RNA-protein)	protein	1,691 × 15,486 / 283
E13 Mouse Embryo	Spatial-Mux-seq (H3K27ac-H3K27me3)	H3K27me3	2,102 × 24,333 / 24,333
E13 Mouse Embryo	Spatial ATAC–RNA-seq (RNA-ATAC)	RNA	2,186 × 20,900 / 87,173
E18.5 Mouse Brain	MISAR-seq (RNA-ATAC)	ATAC	2,129 × 32,285 / 161,461
Human Lymph Node	10x Genomics Visium (RNA-protein)	RNA	3,484 × 18,085 / 31

Table S1: Experimental datasets used in the manuscript

	totalVI	MultiVI	scMDC	SpatialGlue	PRESENT	PRAGA	COSMOS	CANDIES
RNA & protein	✓	✓	✓	✓	✓	✓	✓	✓
RNA & ATAC	×	✓	✓	✓	✓	×	✓	✓

Table S2: Applicability of integration methods for different data types

**Modality 1(RNA)**						**Modality 2(Protein)**				
ZINB			Gaussian noise		Dimension	NB		Gaussian noise		Dimension
π	bkg_mean	expr_mean	mean	std		bkg_mean	expr_mean	mean	std	
0.2	0.5	1.5	2	2.0	800	2	3	2	1.0	100
0.2	0.5	1.5	2	2.5	800	2	3	2	1.0	100
0.2	0.5	1.5	2	3.0	800	2	3	2	1.0	100
0.2	0.5	1.5	2	3.5	800	2	3	2	1.0	100
0.2	0.5	1.5	2	4.0	800	2	3	2	1.0	100
0.2	0.5	1.5	2	4.5	800	2	3	2	1.0	100
0.2	0.5	1.5	2	5.0	800	2	3	2	1.0	100

Table S3: Detailed parameter setting of simulated data generation with varying standard deviation

Modality 1(RNA)						Modality 2(Protein)				
ZINB			Gaussian		Dimension	NB		Gaussian noise		Dimension
π	bkg_mean	expr_mean	mean	std		bkg_mean	expr_mean	mean	std	
0.1	0.5	1.5	2	3	800	2	3	2	1	100
0.2	0.5	1.5	2	3	800	2	3	2	1	100
0.3	0.5	1.5	2	3	800	2	3	2	1	100
0.4	0.5	1.5	2	3	800	2	3	2	1	100

Table S4: Detailed parameter setting of simulated data generation with varying dropout rate

Cluster Method	RNA				ADT				Condition Modality
	Silhouette ↑	DBI ↓	CHI ↑	Moran's I ↑	Silhouette ↑	DBI ↓	CHI ↑	Moran's I ↑	
kmeans	0.054908	3.722839	51.722596	0.642575	0.068931	3.454493	61.351897	0.867207	ADT
leiden	0.048348	4.018209	43.572158	0.686003	0.068317	3.504401	60.771311	0.835424	ADT
mclust	0.051944	4.202843	48.803004	0.774588	0.053446	3.875602	45.541555	0.921345	ADT

Table S5: Selection of condition modality for different clustering methods on simulated dataset

Cluster Method	RNA				ADT				Condition Modality
	Silhouette ↑	DBI ↓	CHI ↑	Moran's I ↑	Silhouette ↑	DBI ↓	CHI ↑	Moran's I ↑	
kmeans	0.056674	4.442508	96.979916	0.354281	0.062148	3.016452	102.537610	0.429725	ADT
leiden	-0.073093	5.249265	38.153515	0.408976	0.054549	3.188531	94.683464	0.453294	ADT
mclust	0.036857	4.269030	80.520900	0.305829	0.084479	2.942547	122.851440	0.541877	ADT

Table S6: Selection of condition modality for different clustering methods on human skin dataset

Cluster Method	H3K27ac				H3K27me3				Condition Modality
	Silhouette ↑	DBI ↓	CHI ↑	Moran's I ↑	Silhouette ↑	DBI ↓	CHI ↑	Moran's I ↑	
leiden	0.032303	3.202254	72.66329	0.767353	0.071240	2.593456	83.43229	0.840121	H3K27me3

Table S7: Selection of condition modality on E13 mouse embryo dataset generated by spatial-Mux-seq platform

Complex Trait	Abbreviation	Category	Link
Intelligence Quotient	IQ	Psychiatric	https://www.nature.com/articles/s41588-018-0152-6
Height	Height	Anthropometric	https://www.nature.com/articles/s41586-022-05275-y
Schizophrenia	SCZ	Psychiatric	https://www.nature.com/articles/s41586-022-04434-5
Major Depressive Disorder	MDD	Psychiatric	https://www.nature.com/articles/s41593-018-0326-7
Mean Corpuscular Hemoglobin Concentration	MCHC	Haemotological	https://www.nature.com/articles/s41588-018-0047-6
Mean Corpuscular Hemoglobin	MCH	Haemotological	https://www.nature.com/articles/s41588-018-0047-6
Body Mass Index	BMI	Anthropometric	https://www.nature.com/articles/s41588-018-0144-6
Mean Corpuscular Volume	MCV	Haemotological	https://www.nature.com/articles/s41588-018-0047-6
Basophils	Baso	Haemotological	https://www.cell.com/cell/fulltext/S0092-8674(20)30999-5
Monocytes	Mono	Haemotological	https://www.cell.com/cell/fulltext/S0092-8674(20)30999-5
Neutrophils	Neutro	Haemotological	https://www.cell.com/cell/fulltext/S0092-8674(20)30999-5
Eosinophils	Eosino	Haemotological	https://www.cell.com/cell/fulltext/S0092-8674(20)30999-5
Platelets	Plt	Haemotological	https://www.cell.com/cell/fulltext/S0092-8674(20)30999-5
Red Blood Cells	RBC	Haemotological	https://www.cell.com/cell/fulltext/S0092-8674(20)30999-5
Hemoglobin	Hb	Haemotological	https://www.cell.com/cell/fulltext/S0092-8674(20)30999-5
Lymphocytes	Lym	Haemotological	https://www.cell.com/cell/fulltext/S0092-8674(20)30999-5
White Blood Cells	WBC	Haemotological	https://www.cell.com/cell/fulltext/S0092-8674(20)30999-5
High-Density Lipoprotein	HDL	Metabolism	https://csg.sph.umich.edu/willer/public/glgc-lipids2021/
Low-Density Lipoprotein	LDL	Metabolism	https://csg.sph.umich.edu/willer/public/glgc-lipids2021/
Total Cholesterol	TC	Metabolism	https://csg.sph.umich.edu/willer/public/glgc-lipids2021/
Log-transformed Triglycerides	logTG	Metabolism	https://csg.sph.umich.edu/willer/public/glgc-lipids2021/
Attention Deficit Hyperactivity Disorder	ADHD	Psychiatric	https://pgc.unc.edu/for-researchers/download-results/
Alzheimer's Disease	ALZ	Psychiatric	https://pgc.unc.edu/for-researchers/download-results/
Anxiety Disorder	ANX	Psychiatric	https://pgc.unc.edu/for-researchers/download-results/
Autism Spectrum Disorder	ASD	Psychiatric	https://pgc.unc.edu/for-researchers/download-results/
Bipolar Disorder	BIP	Psychiatric	https://pgc.unc.edu/for-researchers/download-results/
Obsessive-Compulsive Disorder / Tourette Syndrome	OCDTS	Psychiatric	https://pgc.unc.edu/for-researchers/download-results/
Post-Traumatic Stress Disorder	PTSD	Psychiatric	https://pgc.unc.edu/for-researchers/download-results/
Education attainment	EA	Behaviour	https://www.nature.com/articles/s41588-018-0147-3
Drinks per week	DPW	Behaviour	https://www.nature.com/articles/s41588-018-0307-5
Smoking Cessation	SmokingC	Behaviour	https://www.nature.com/articles/s41588-018-0307-5
Smoking Initiation	SmokingI	Behaviour	https://www.nature.com/articles/s41588-018-0307-5

Table S8: 32 complex traits used for downstream analysis

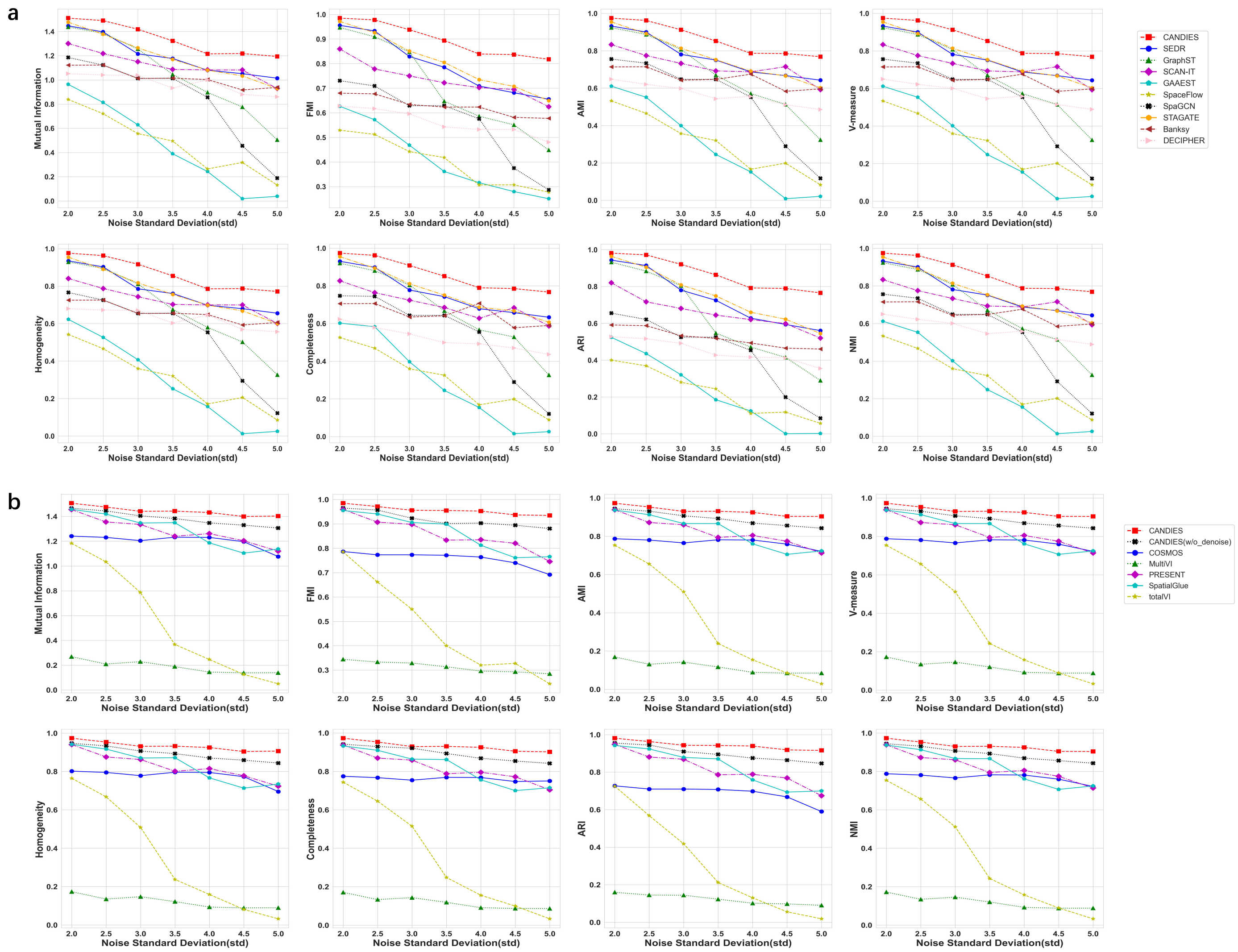

Fig. S1: **Quantitative analysis of simulated data generated with varying standard deviations. a**, Spatial transcriptome denoising results across eight supervised evaluation metrics. **b**, Multi-omics integration results across eight supervised evaluation metrics.

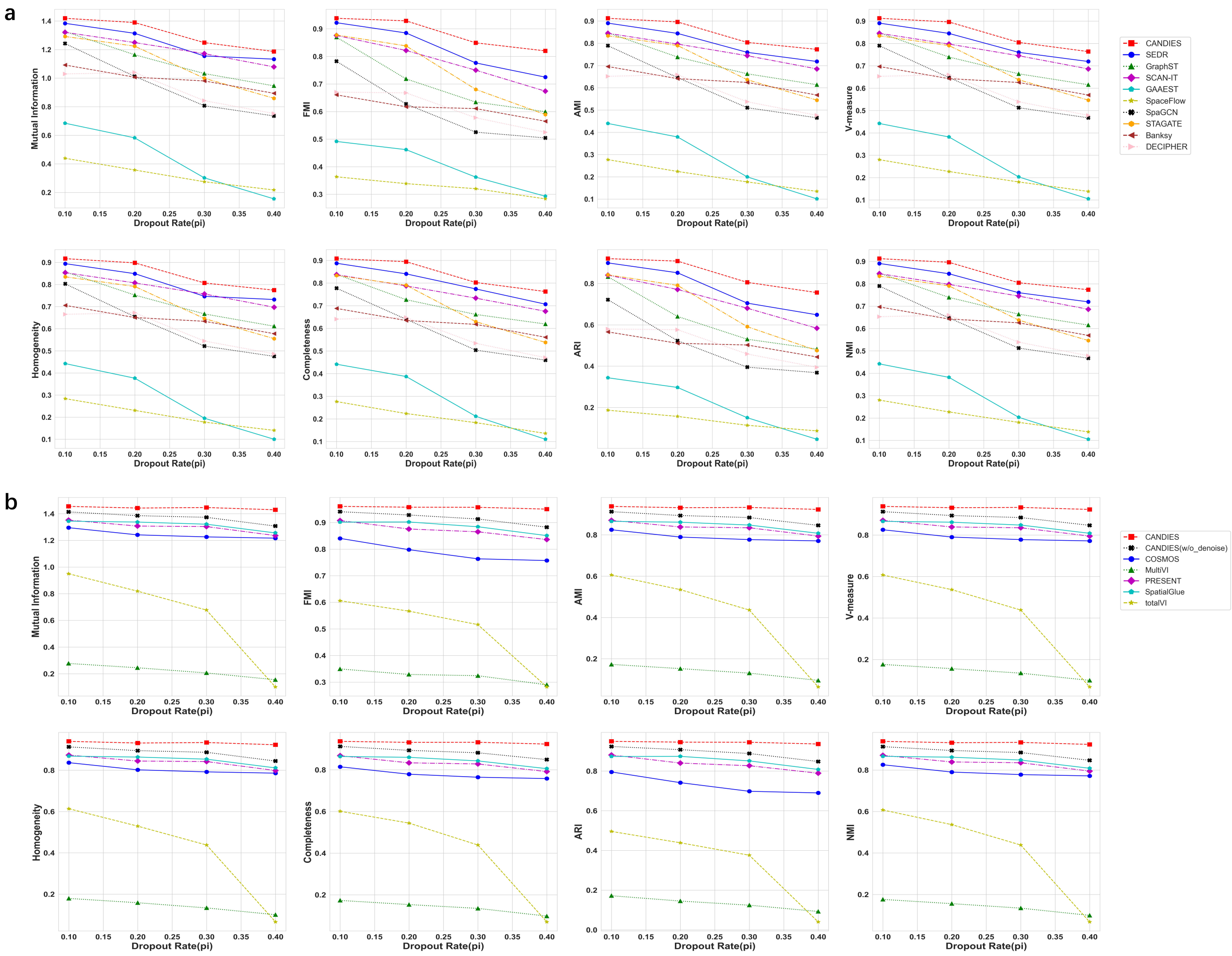

Fig. S2: **Quantitative analysis of simulated data generated with varying dropout rate. a**, Spatial transcriptome denoising results across eight supervised evaluation metrics. **b**, Multi-omics integration results across eight supervised evaluation metrics.

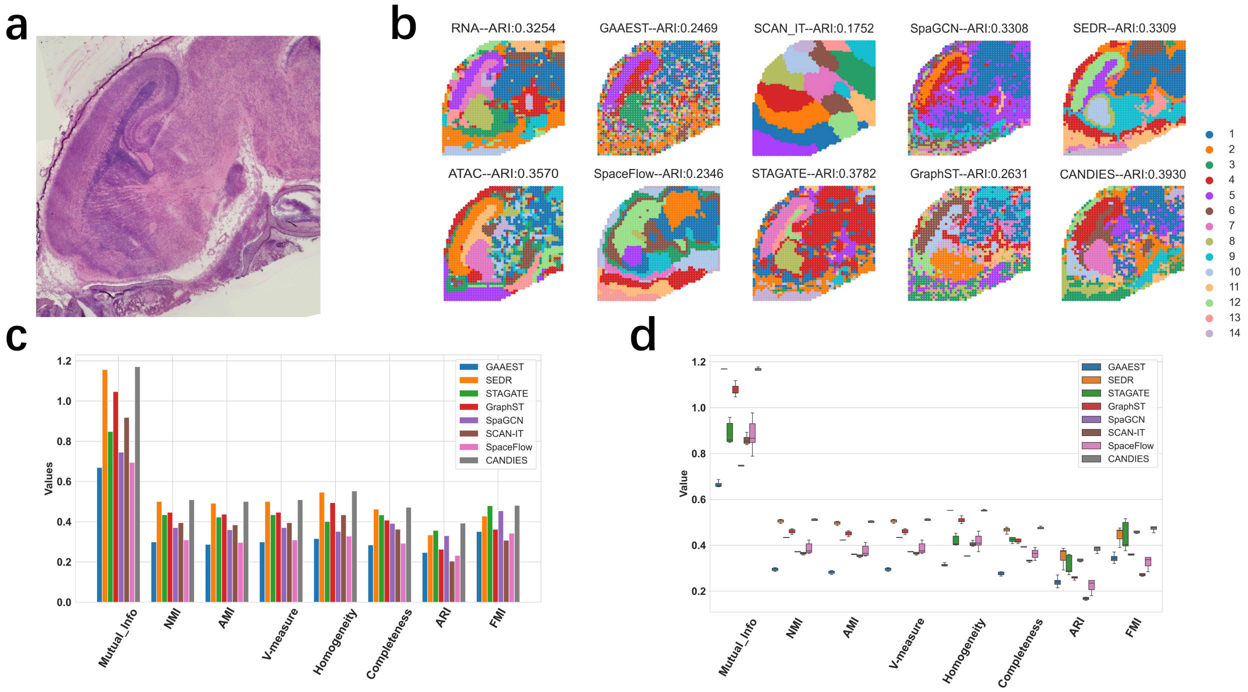

Fig. S3: **Denoising results for E18.5 mouse brain data. a**, The bright-field image of the E18.5 mouse brain tissue section. **b**, Spatial plots of the E18.5 mouse brain data only with RNA modality across eight representative spatial transcriptomics (ST) methods, the first column is identified by Leiden after encoding phase. **c**, Bar plots comparing the performance of eight methods across eight supervised metrics. **d**, Box plots comparing the performance of eight methods across eight supervised metrics.

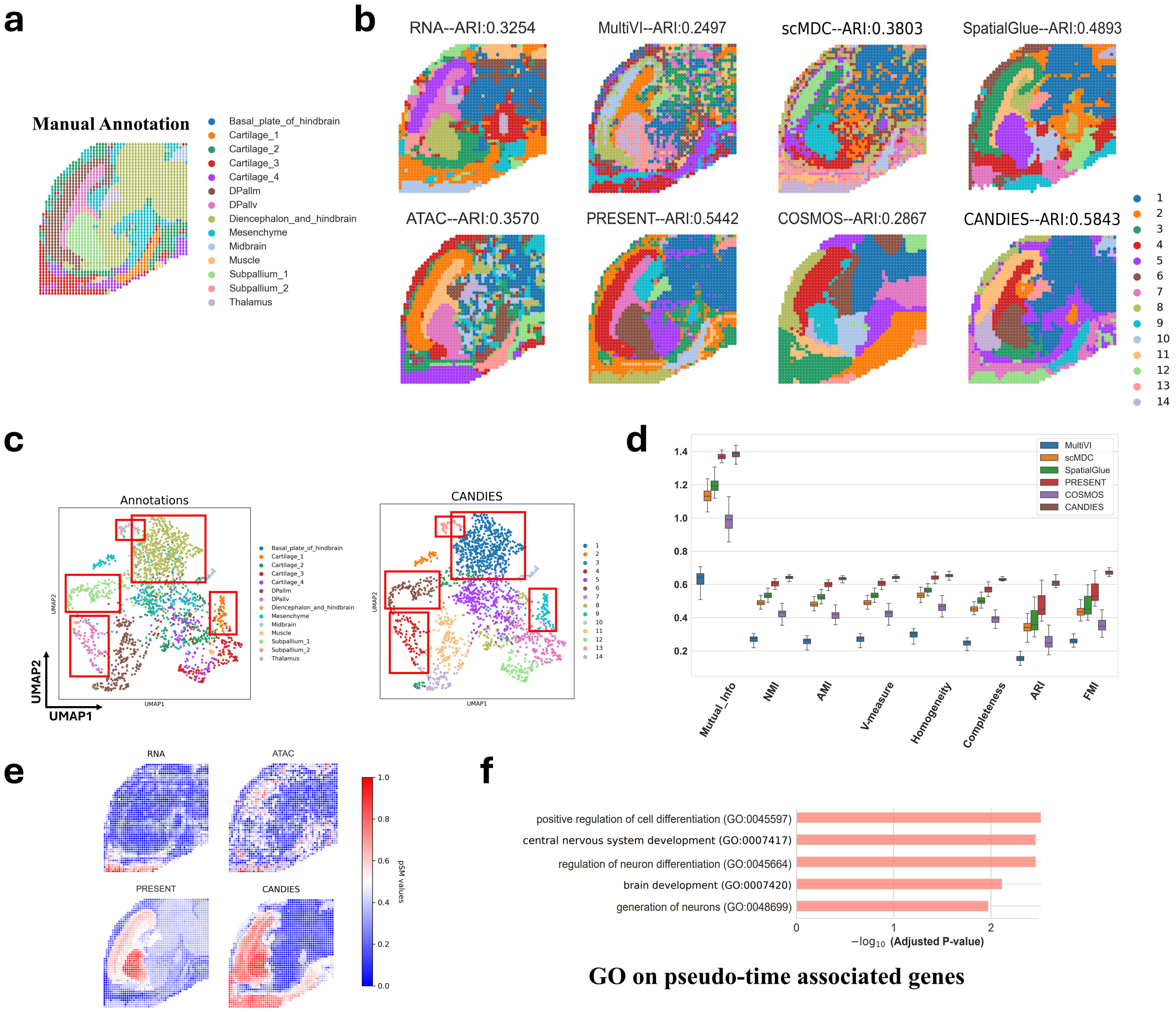

Fig. S4: **Integration results for E18.5 mouse brain data.** **a**, Manual annotation of the E18.5 mouse brain data. **b**, Spatial plots of the E18.5 mouse brain data across six single-cell and spatial multi-omics integration methods, the first column is identified by Leiden on each modality. **c**, The UMAP visualization of CANDIES' latent representations colored by manual annotation (left) and CANDIES (right). **d**, Box plots of the eight supervised metrics across the six methods. **e**, Pseudo spatiotemporal maps (pSM) generated by each individual modality, PRESENT and CANDIES. **f**, Gene Ontology Biological Process (GOBP) enrichment analysis of pseudo-time associated genes.

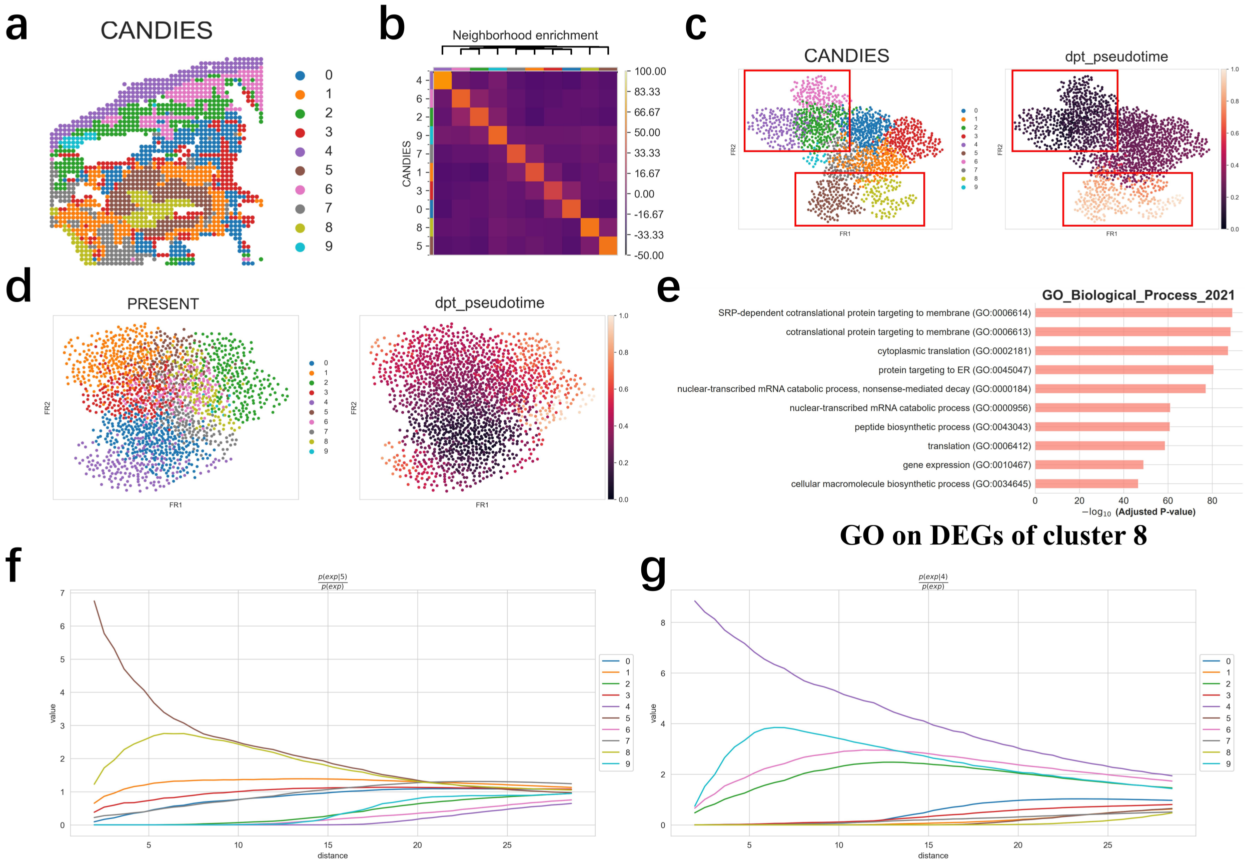

Fig. S5: **Additional results of the human skin data after integration.** **a**, Spatial plot of CANDIES integration clustering result. **b**, Similarity heatmap of clusters identified by CANDIES, illustrating the spatial proximity relationships between different clusters. **c**, The UMAP visualization of CANDIES' latent representations colored by clusters (left) and pSM values (right). **d**, The UMAP visualization of PRESENT's latent representations colored by clusters (left) and pSM values (right). **e**, Gene Ontology (GO) enrichment analysis on differentially expressed genes (DEGs) of cluster 8. **f**, Cluster co-occurrence score for cluster 5 at increasing distances. **g**, Cluster co-occurrence score for cluster 4 at increasing distances.

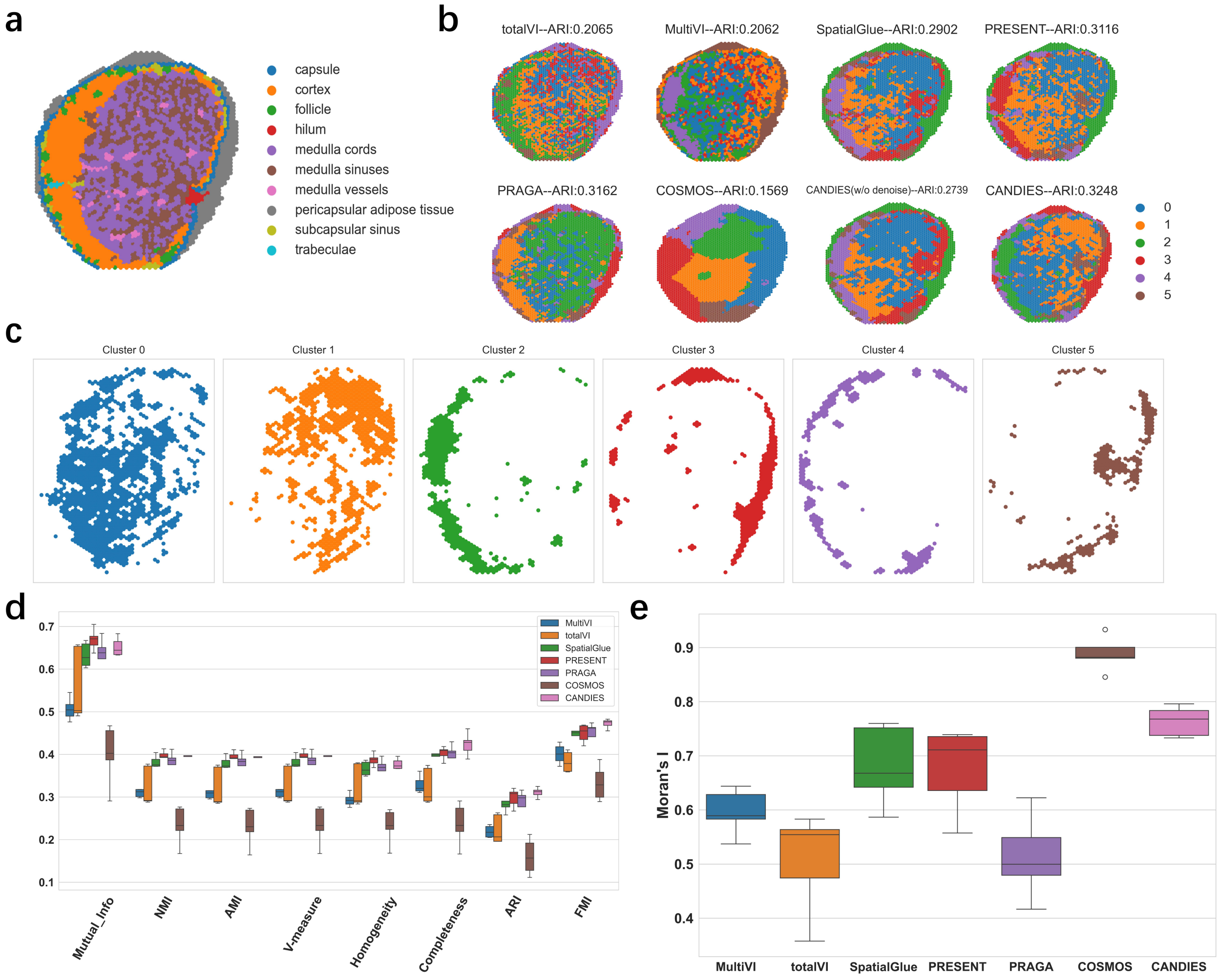

Fig. S6: **Integration results for human lymph node data. a**, Manual annotation of the human lymph node data. **b**, Spatial plots of the human lymph node data across seven single-cell and spatial multi-omics integration methods, CANDIES (w/o denoise) refers to the variant of CANDIES without the denoising phase. **c**, Separate spatial plots of all clusters identified by CANDIES in the human lymph node data. **d**, Box plots of the eight supervised metrics across the seven methods. **e**, Box plots of the Moran's I scores across the seven methods.

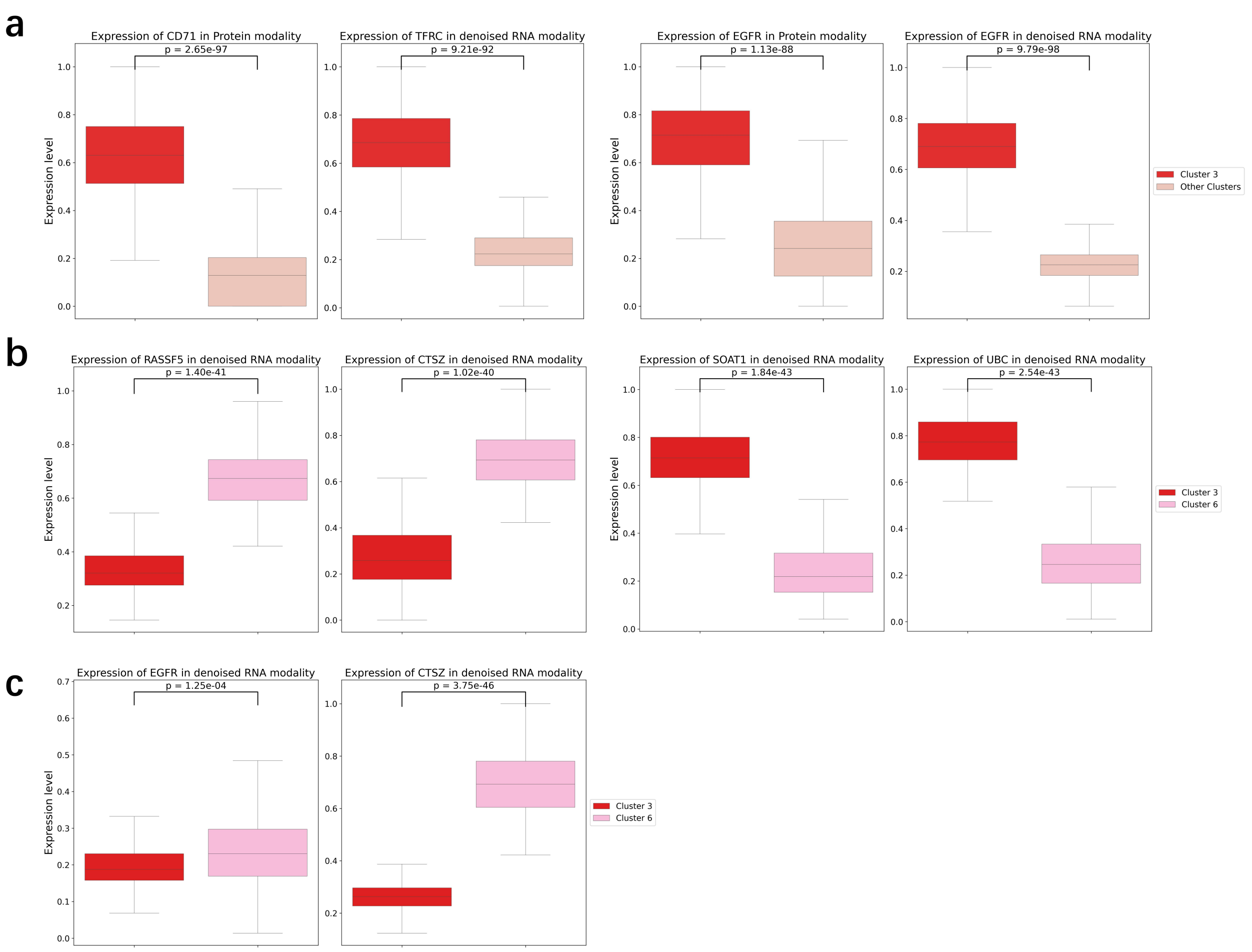

Fig. S7: **Analysis of the cluster 3 and cluster 6 identified by CANDIES on RNA modality of human skin data.** **a**, Expression levels of *CD71* and *TFRC* across Protein and denoised RNA modalities in the left of box plots, as well as *EGFR* and *EGFR* in the right of box plots. P-values were calculated using the Mann-Whitney U test. **b**, Expression levels of *RASSF5*, *CTSZ*, *SOAT1* and *UBC* across cluster 3 and cluster 6 in denoised RNA modality. **c**, Expression levels of *EGFR* and *CTSZ* across cluster 3 and cluster 6 in denoised RNA modality.

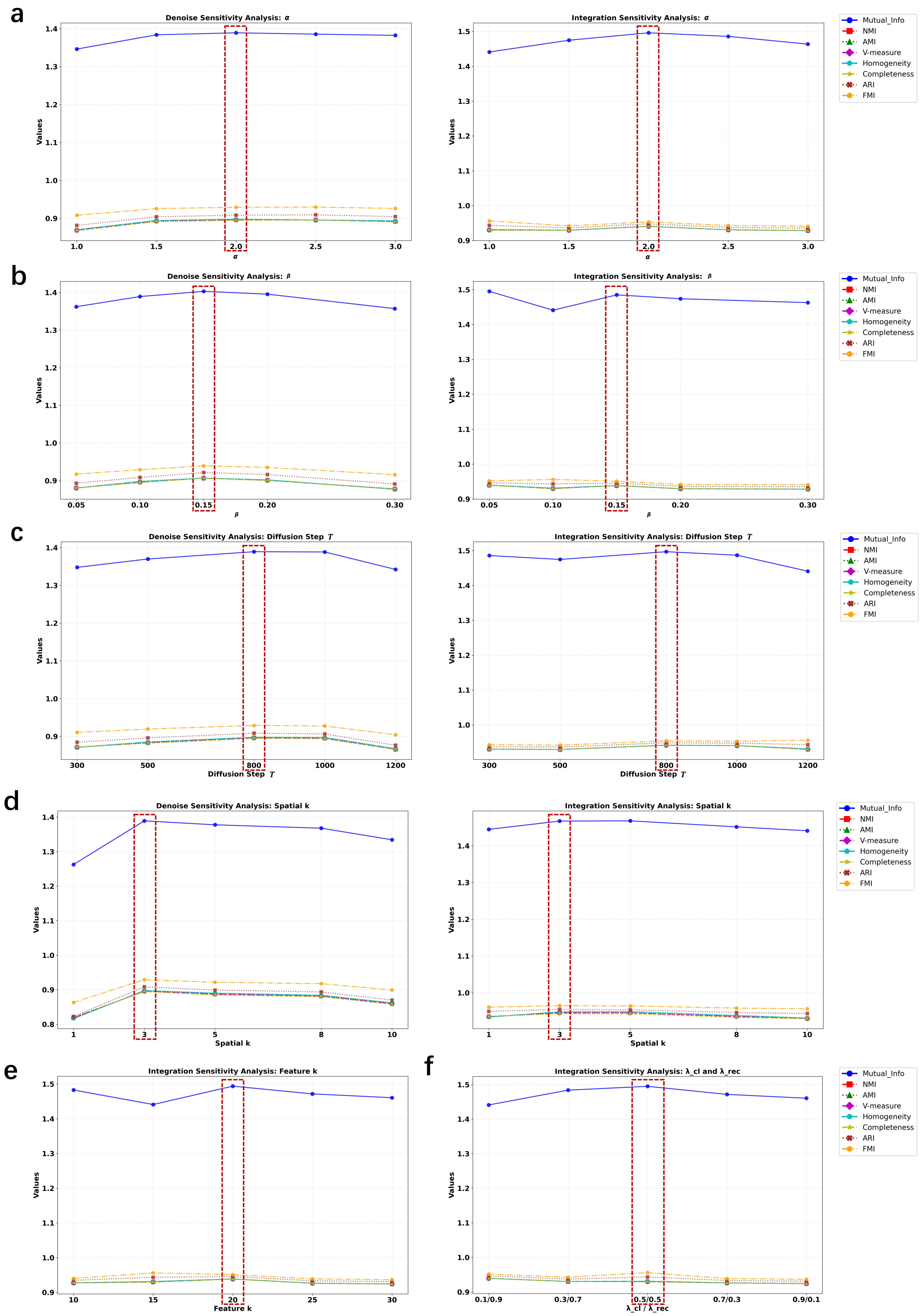

Fig. S8: **Analysis of parameter sensitivity on simulated data.** **a**, Comparison of eight supervised metrics across different values of α in Eqs. (4) and (6), obtained from denoising results (left panel) and integration results (right panel), the red dashed box indicates the default setting. **b**, Comparison of eight supervised metrics across different values of β in Eqs. (4) and (6), obtained from denoising results (left panel) and integration results (right panel). **c**, Comparison of eight supervised metrics across different diffusion steps T in Eq. (10), obtained from denoising results (left panel) and integration results (right panel). **d**, Comparison of eight supervised metrics across different numbers of spatial neighbors k, obtained from denoising results (left panel) and integration results (right panel). **e**, Comparison of eight supervised metrics across different numbers of feature neighbors k, obtained from integration results. **f**, Comparison of eight supervised metrics across different trade off between λ_{rec} and λ_{cl} in Eq. (22), obtained from integration results.

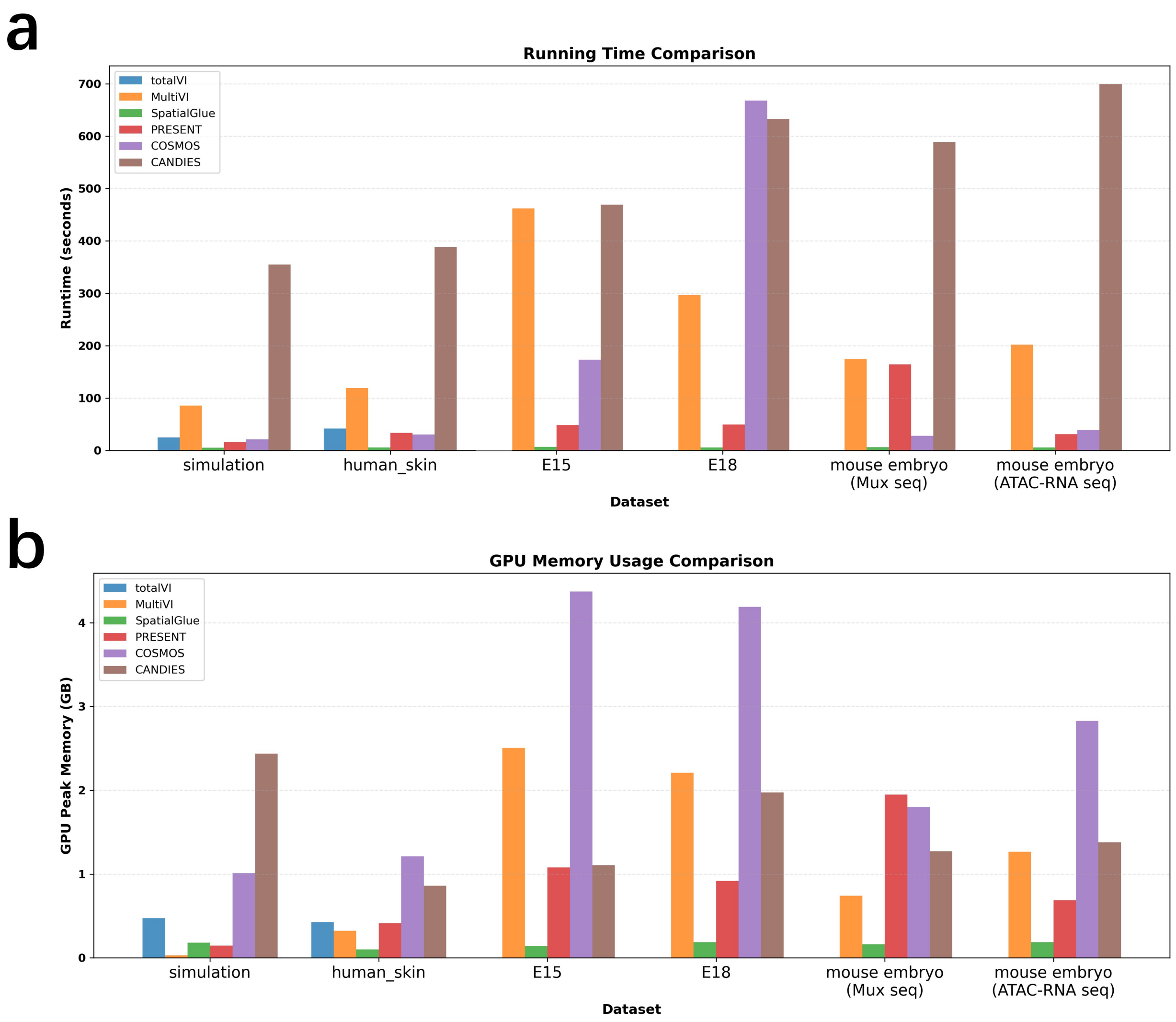

Fig. S9: **Analysis of running time and GPU memory usage across multiple datasets with representative baseline methods. a**, Comparison of time complexity of CANDIES and competing methods (totalVI, MultiVI, SpatialGlue, PRESENT, COSMOS) across six datasets of different scales. **b**, Comparison of GPU memory usage of CANDIES and competing methods across six datasets of different scales.

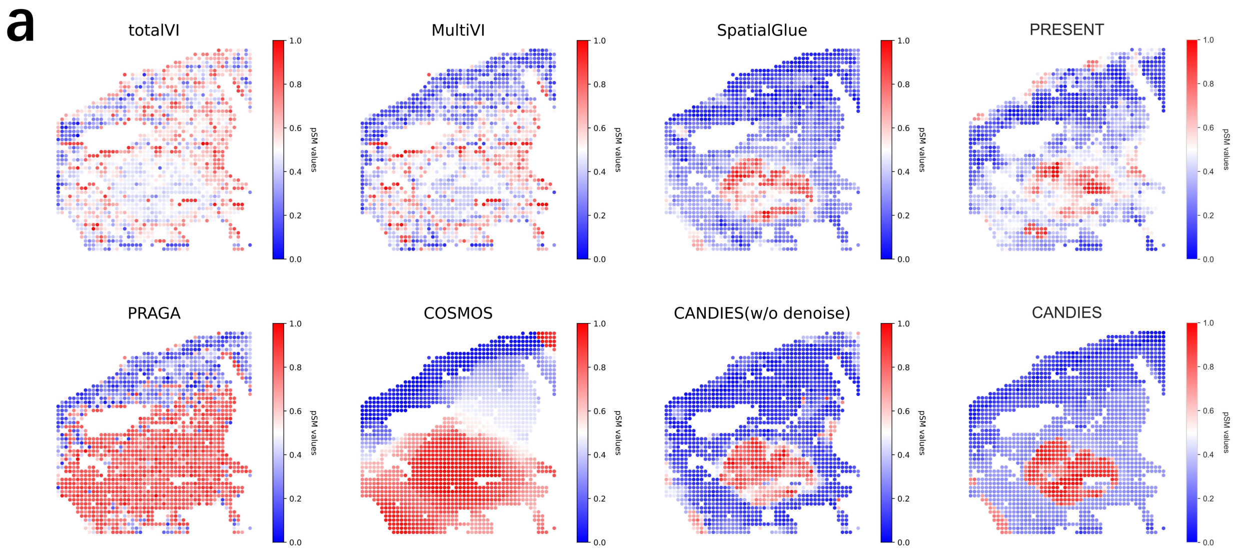

Fig. S10: **Analysis of pseudo spatiotemporal maps on human skin data. a**, Pseudo spatiotemporal maps (pSM) generated by all compared methods (totalVI, MultiVI, SpatialGlue, PRESENT, PRAGA, COSMOS).

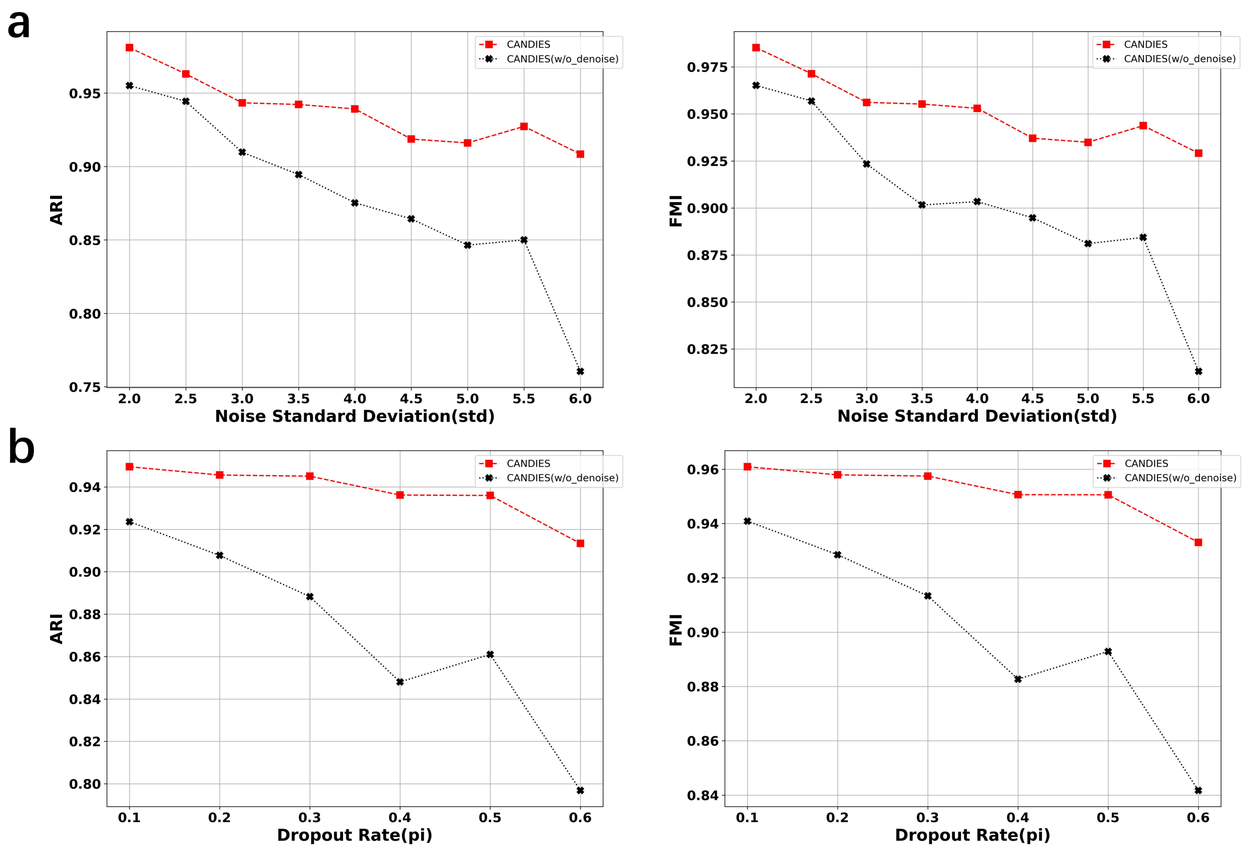

Fig. S11: **Analysis of CANDIES and its variant (CANDIES w/o denoise) across varying noise levels. a**, Comparison of ARI values (left panel) and FMI values (right panel) obtained under different noise standard deviations in RNA modality. **b**, Comparison of ARI values (left panel) and FMI values (right panel) obtained under different dropout rates in RNA modality.

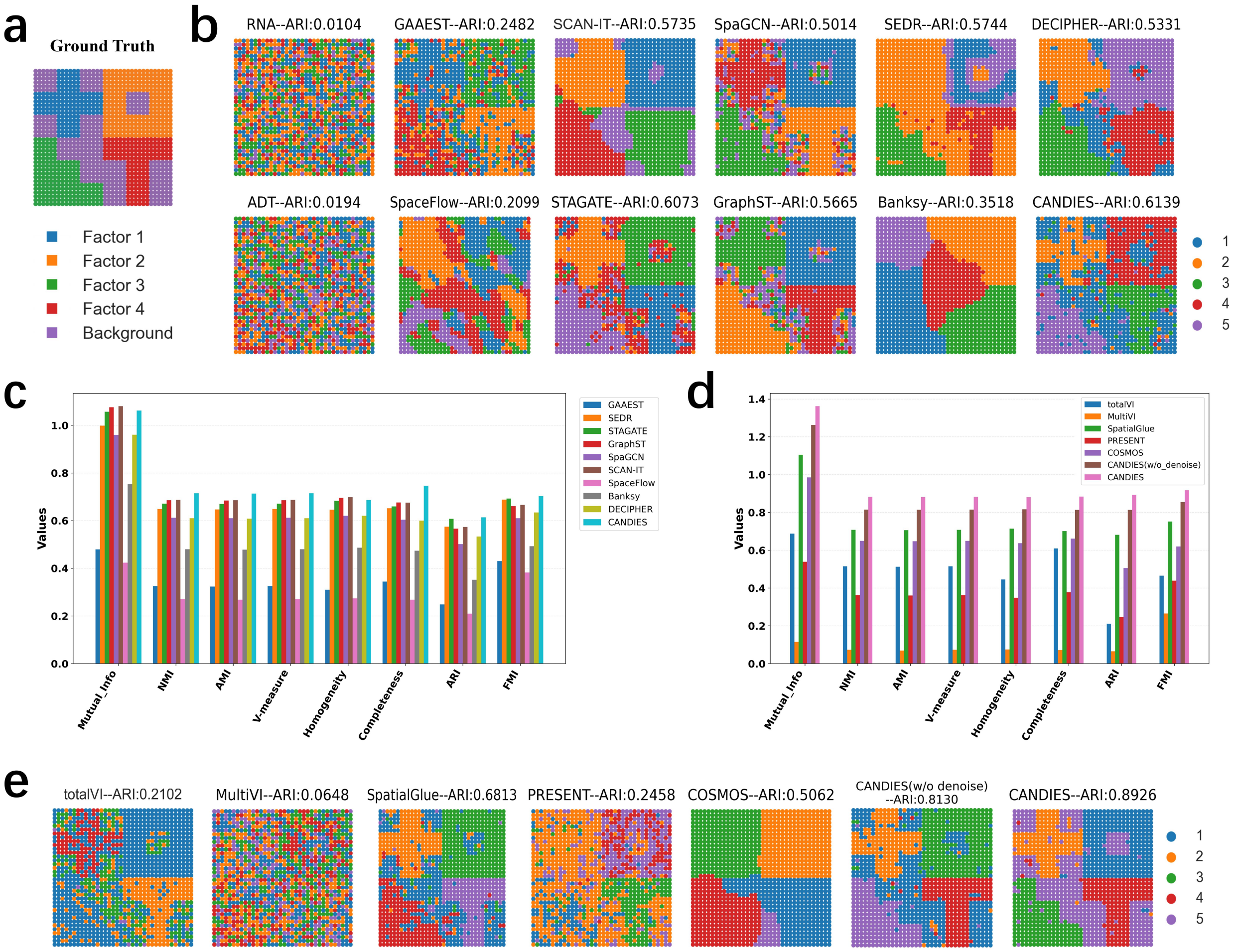

Fig. S12: **Analysis under comparable quality conditions (both modalities are noisy) on simulated data. a**, Ground truth of the simulated spatial multi-omics data. **b**, Spatial plots of the simulated data with only RNA modality across nine methods designed for spatial transcriptomics (ST) data, the first column is identified by Leiden on each modality. **c**, Bar plots of the eight supervised metrics across the ten ST analysis methods. **d**, Bar plots of the eight supervised metrics across the six multi-omics integration methods. **e**, Spatial plots of the simulated data across six single-cell and spatial multi-omics integration methods, CANDIES (w/o denoise) refers to the variant of CANDIES without the denoising phase.

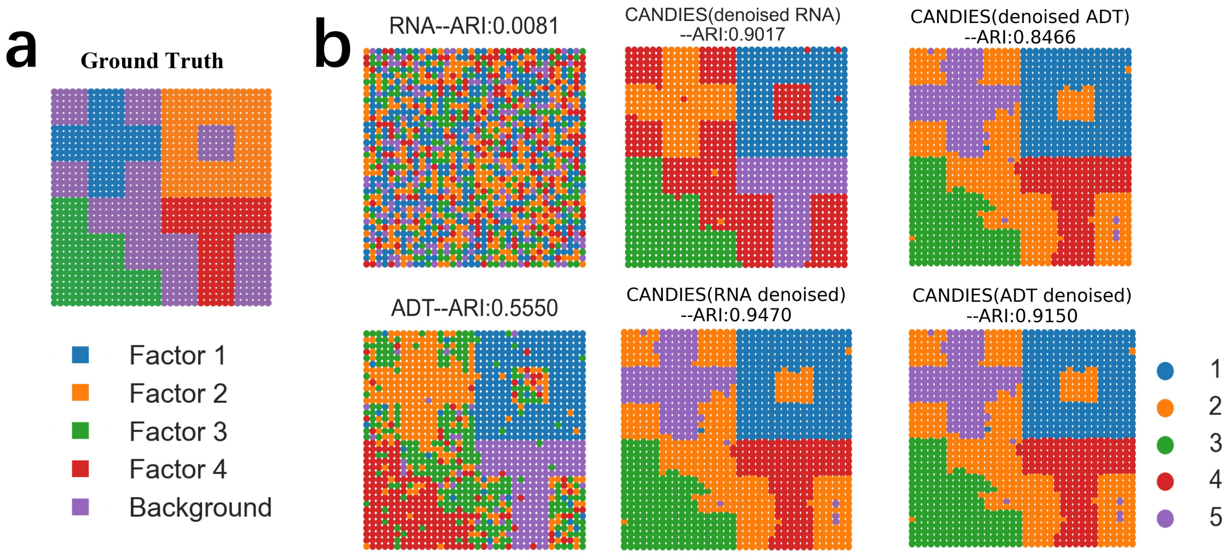

Fig. S13: **Analysis of using the lower-quality modality as the condition in the diffusion model on simulated data. a**, Ground truth of the simulated spatial multi-omics data. **b**, Spatial plots of the simulated data, the first column is identified by Leiden on each modality. The second column shows the RNA denoising and final integration results conditioned on ADT modality (higher quality), and the third column is the ADT denoising and final integration results conditioned on RNA modality (lower quality).

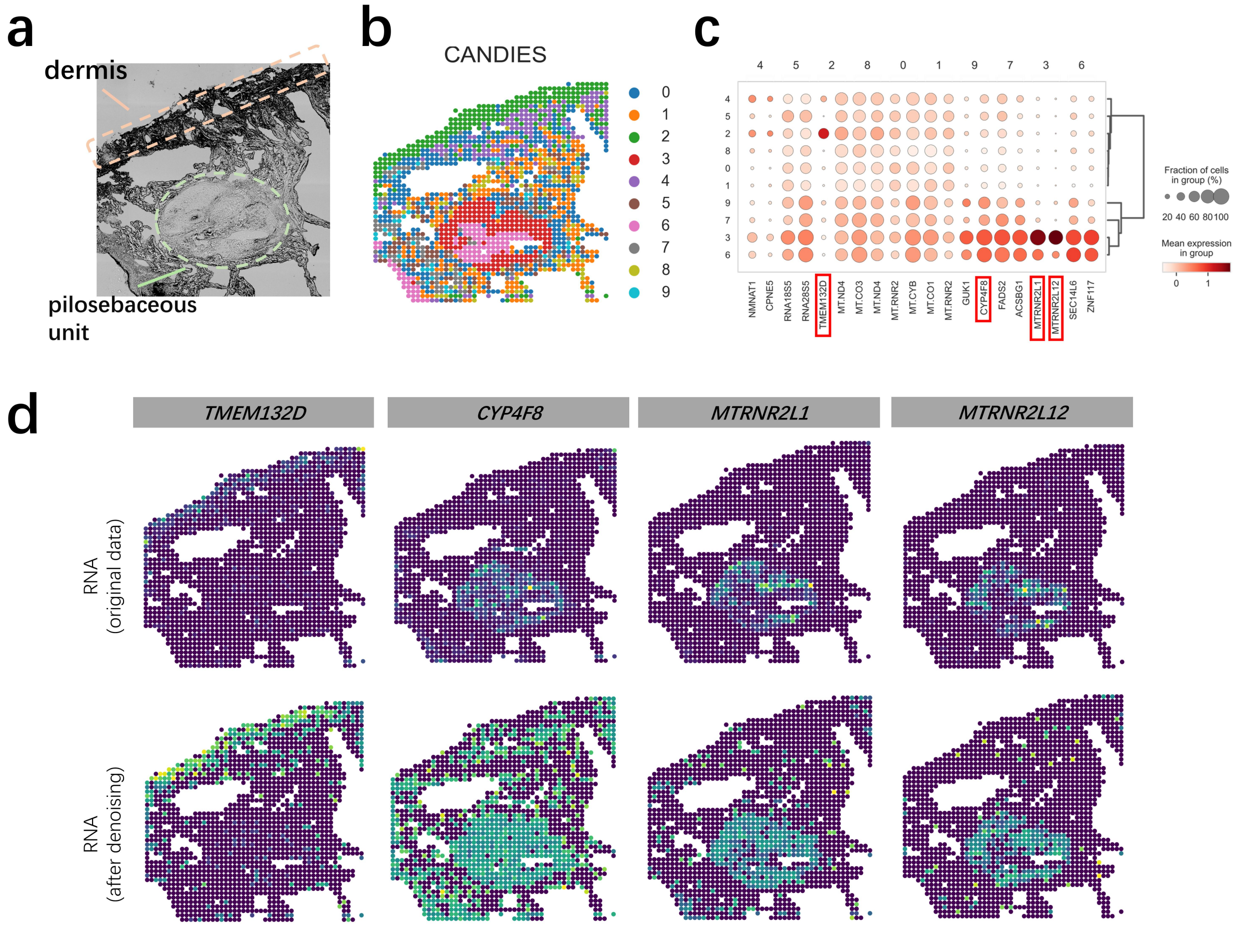

Fig. S14: **Analysis of the denoising capability of CANDIES on human skin data. a**, The bright-field image of the human skin tissue section. Highlighted regions are dermis and pilosebaceous unit. **b**, Spatial plots of the human skin data with RNA modality identified by CANDIES. **c**, Top-2 differentially expressed genes (DEGs) identified for each cluster discovered by CANDIES on the denoised ST dataset. The dot size represents the proportion of spots within a cluster that express the corresponding DEG, and the color intensity denotes the average expression level of that DEG in the cluster. **d**, The first and second rows correspond to spatial distribution of the selected four DEGs before and after denoising.

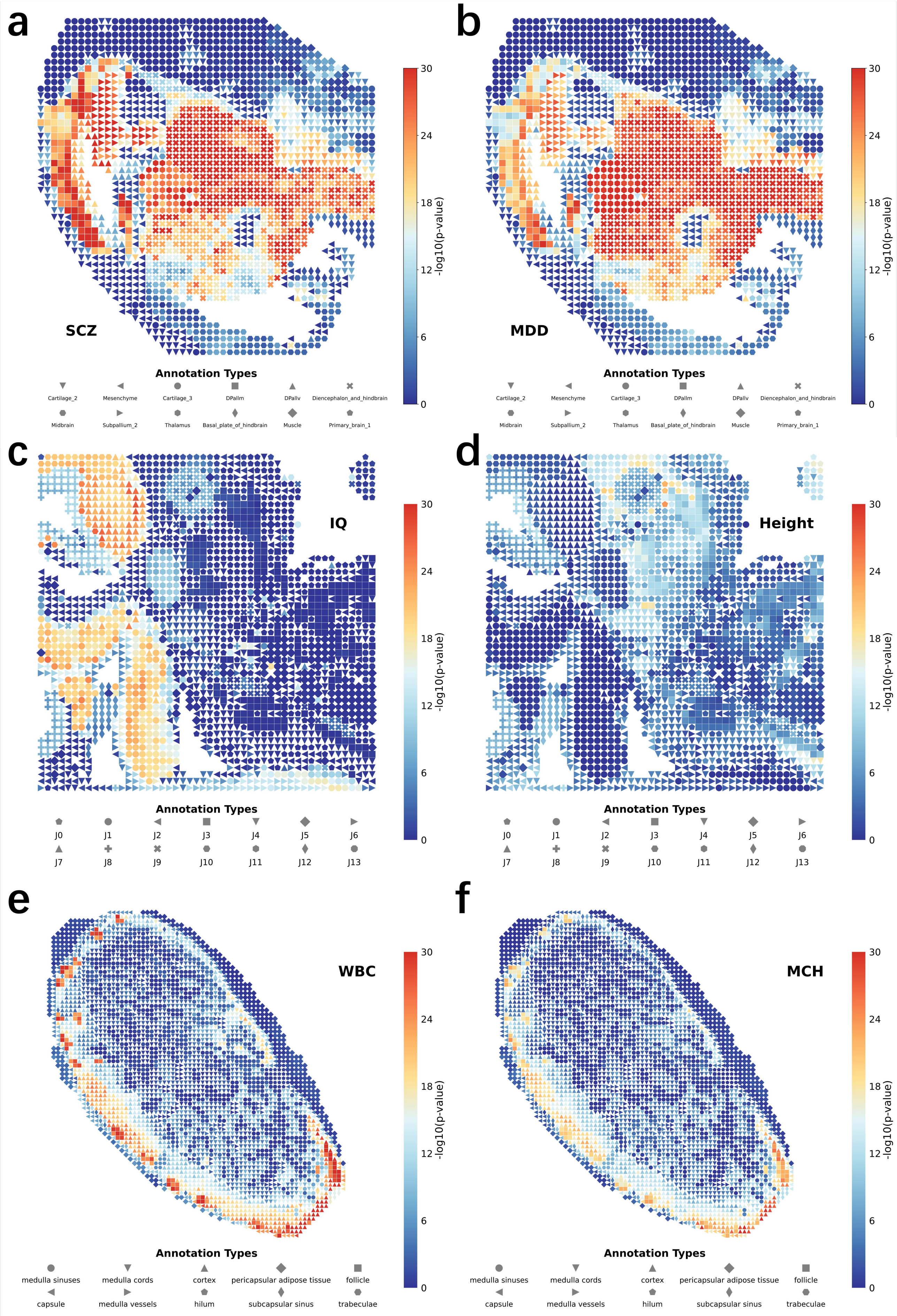

Fig. S15: **Trait-spot associations identified by using gsMap embeddings in three spatial omics datasets as input. a,b**, Two psychiatric traits (major depressive disorder and schizophrenia) highlighted in mouse brain, with spot annotation indicated by different point shapes and $\log_{10} p$-values reflected by the color. **c,d**, Two representative traits (IQ and height) in mouse embryo, illustrating how developmental regions associate with cognition and anthropometric traits. **e,f**, Two hematological traits (white blood cells count and mean corpuscular hemoglobin) in human lymph, illustrating localized enrichment patterns.

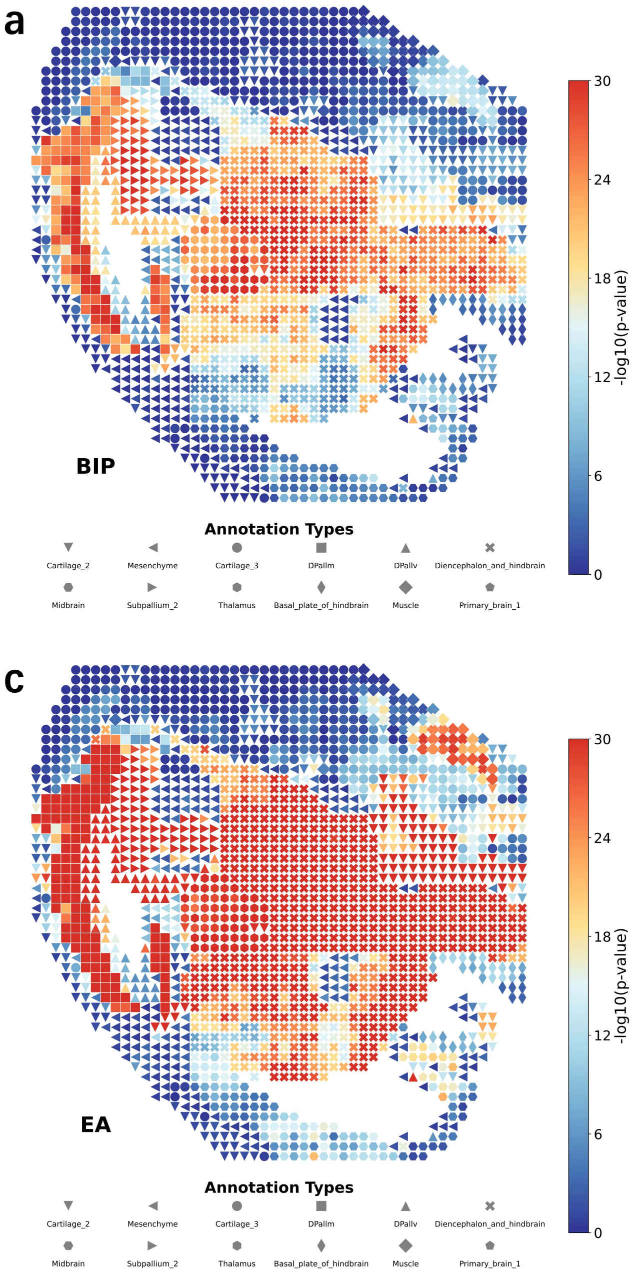

Fig. S16: Trait-spot associations for BIP, IQ, EA and Smoking-I traits identified by using CANDIES embeddings in mouse brain datasets as input.

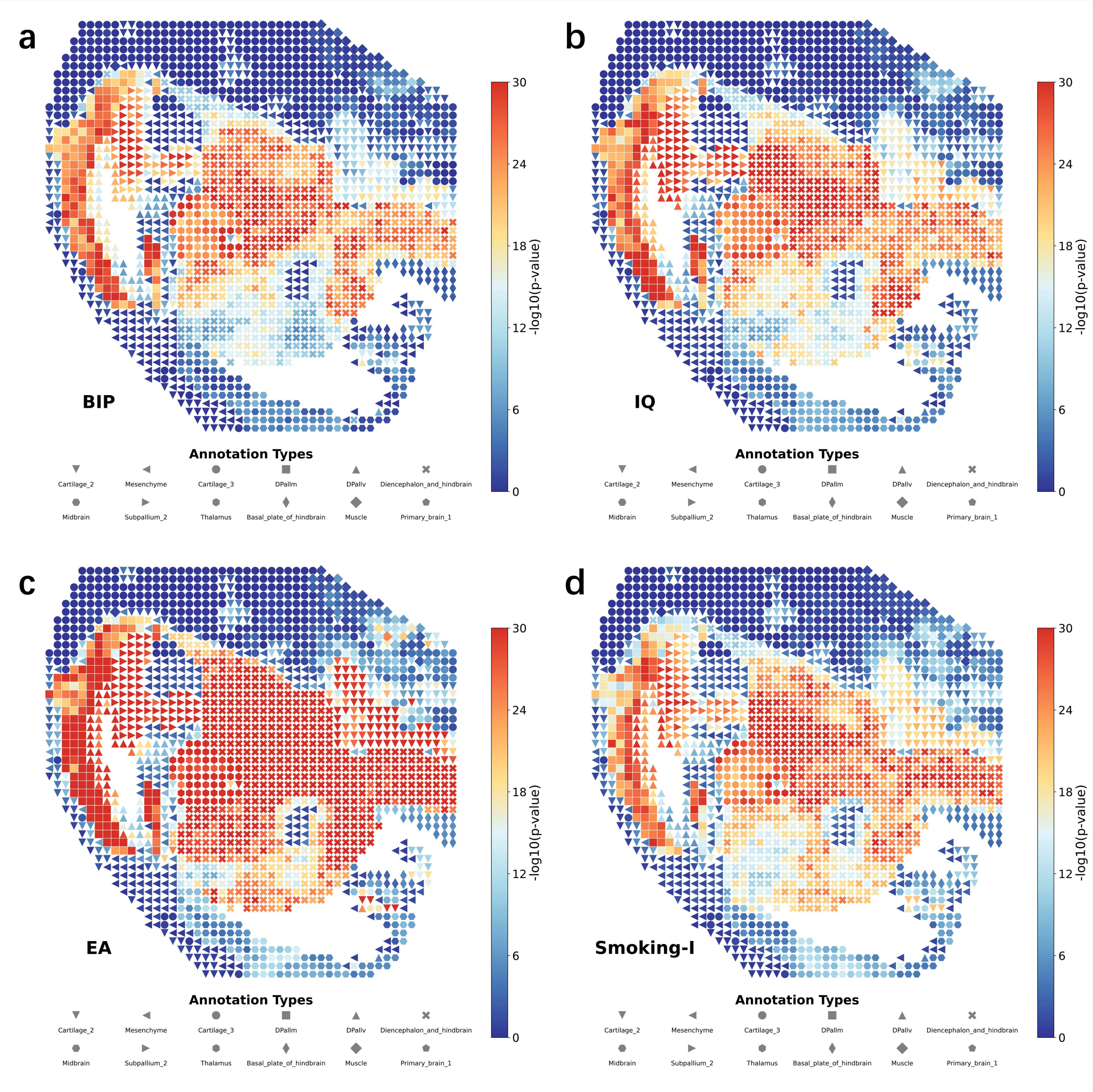

Fig. S17: Trait-spot associations for BIP, IQ, EA and Smoking-I traits identified by using gsMap embeddings in mouse brain datasets as input.

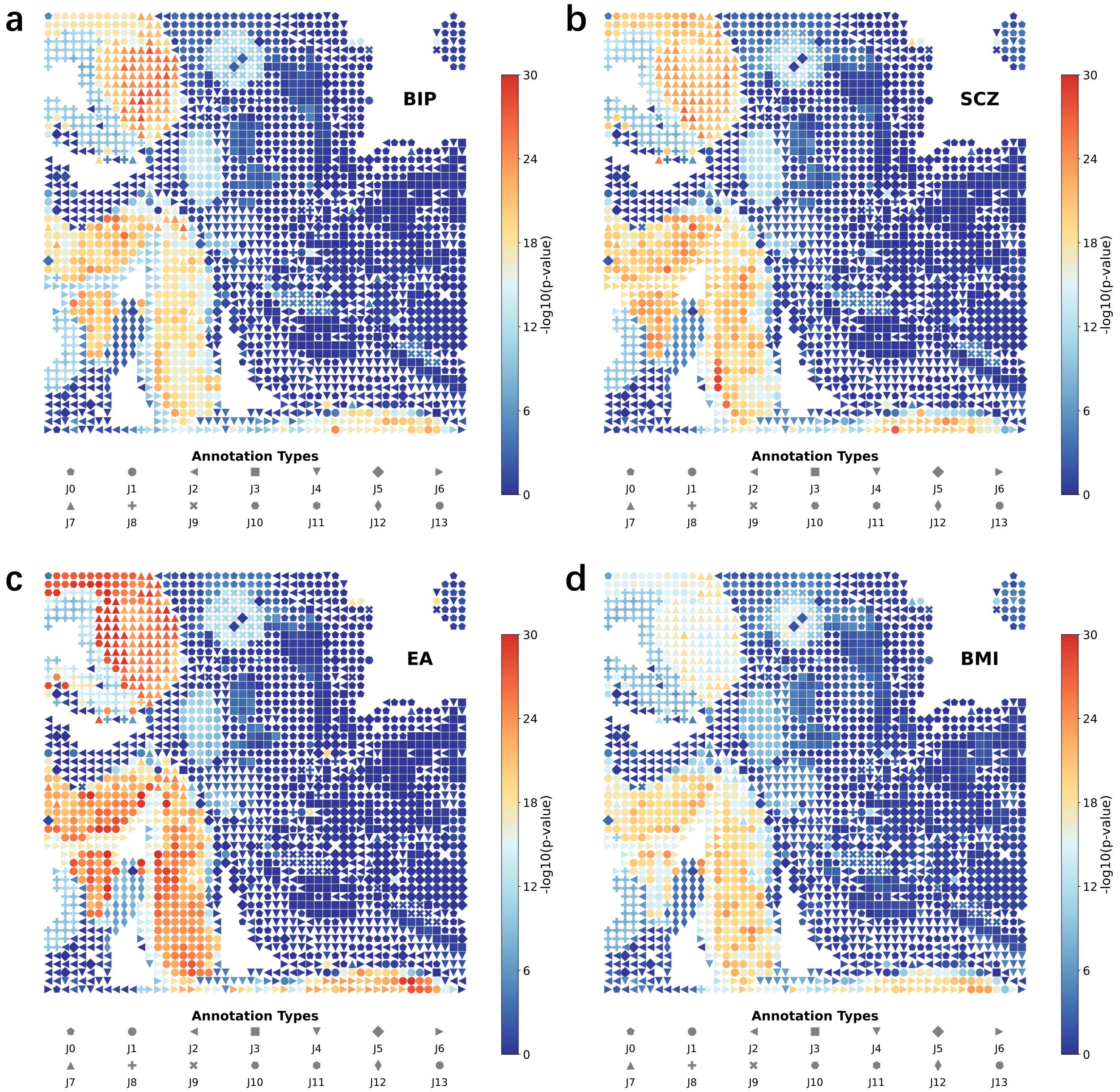

Fig. S18: Trait-spot associations for BIP, SCZ, EA, BMI traits identified by using CANDIES embeddings in mouse embryo datasets as input.

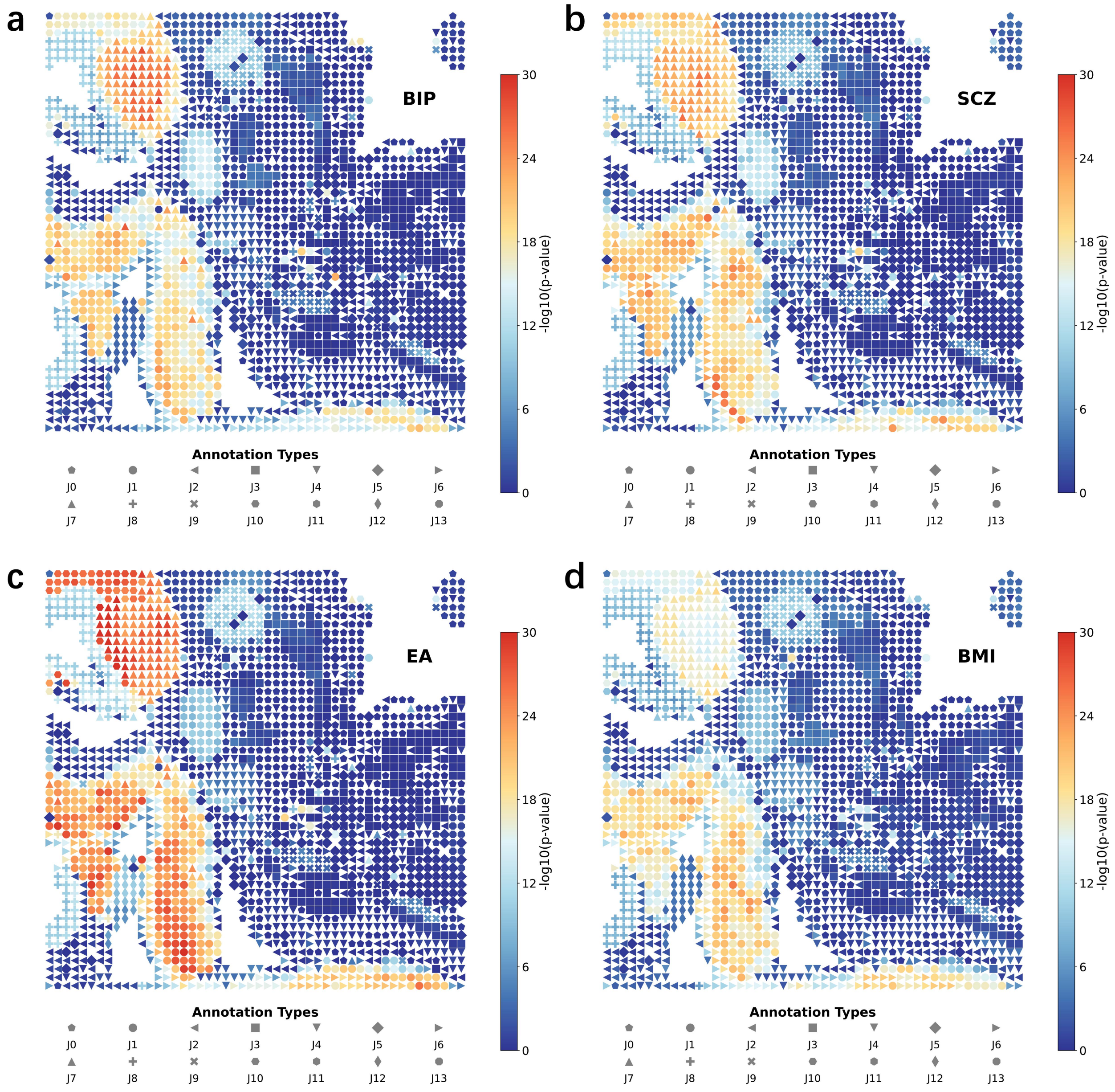

Fig. S19: Trait-spot associations for BIP, SCZ, EA, BMI traits identified by using gsMap embeddings in mouse embryo datasets as input.

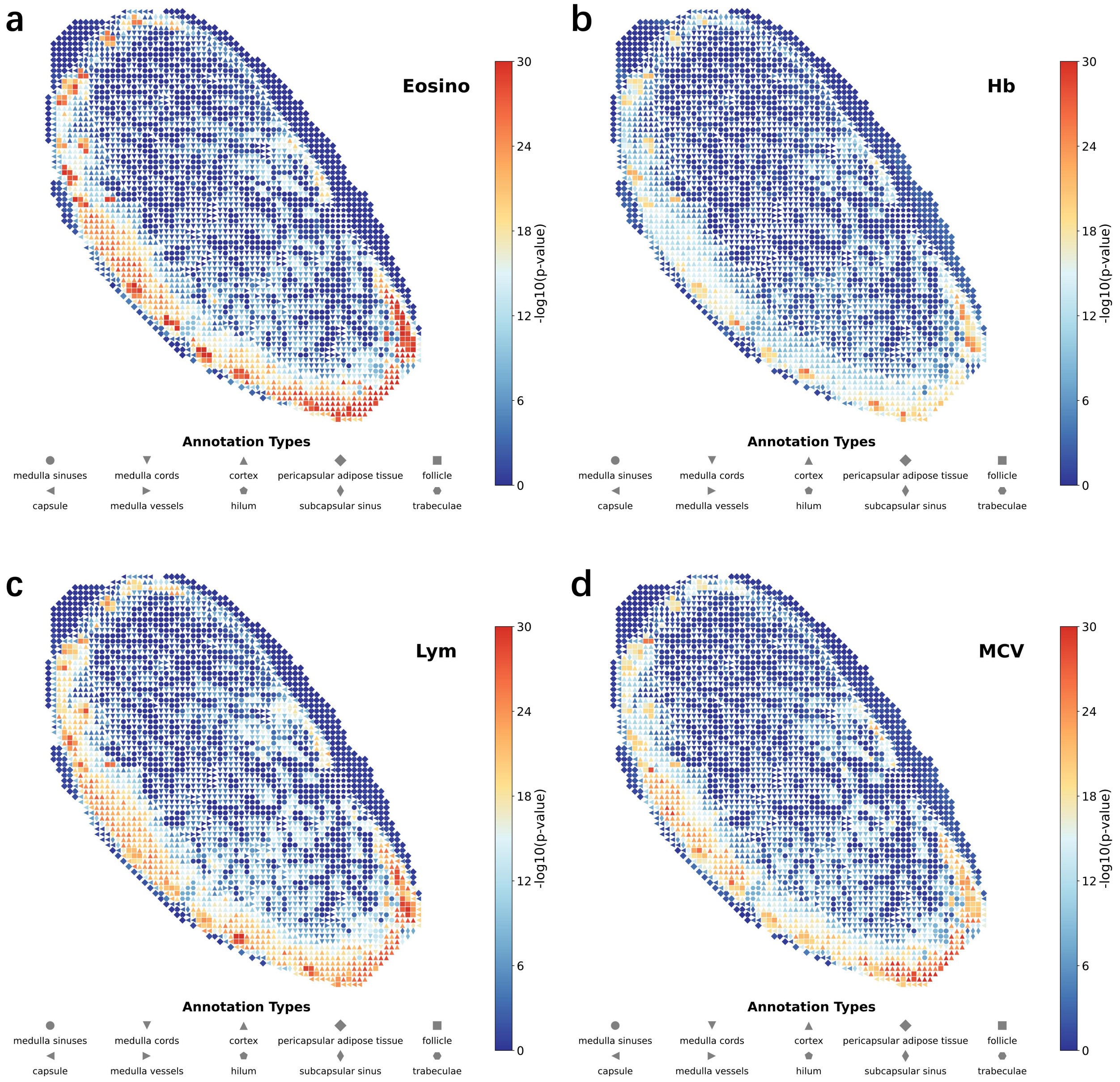

Fig. S20: Trait-spot associations for Eosino, Hb, Lym, MCV traits identified by using CANDIES embeddings in human lymph datasets as input.

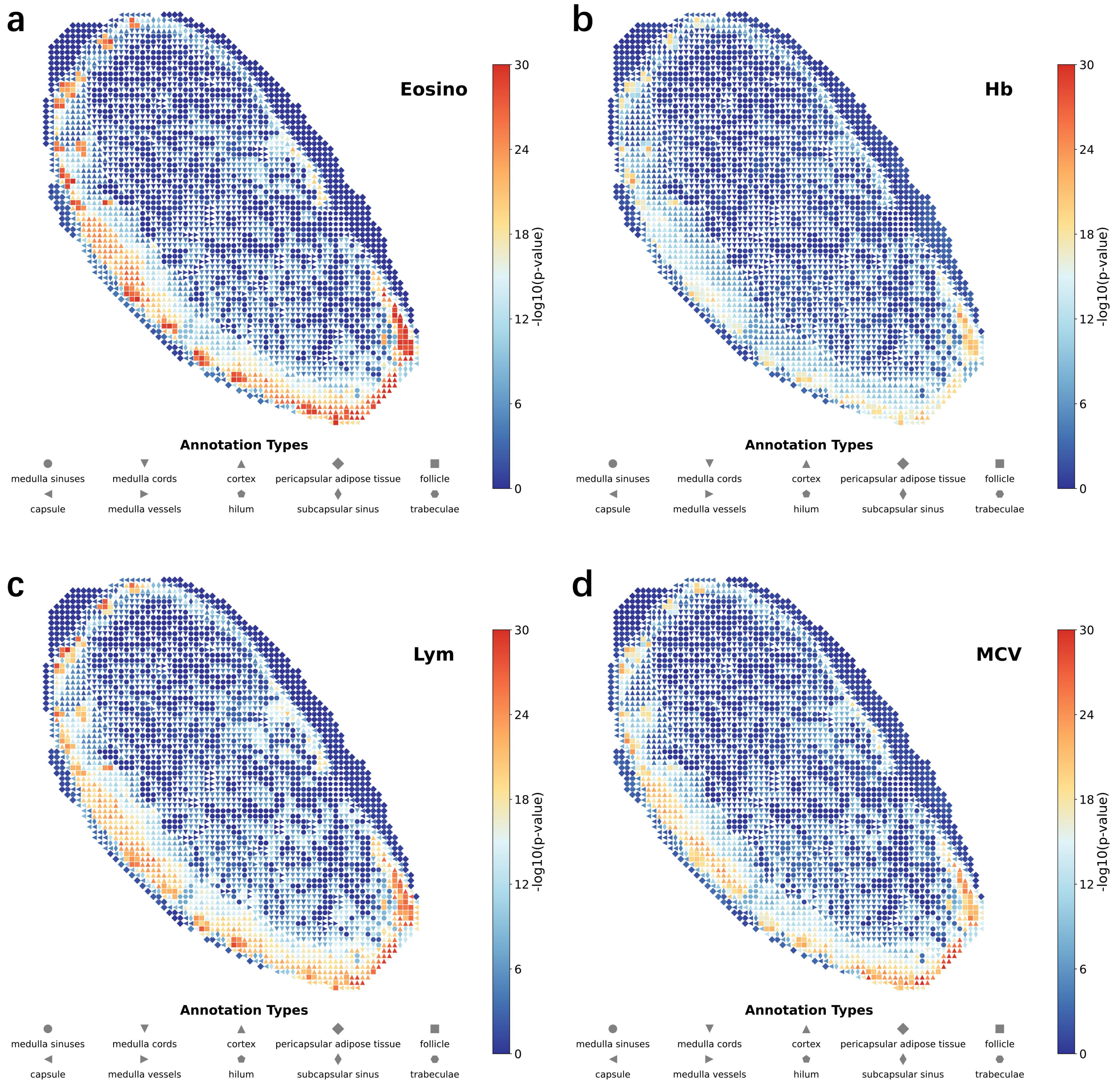

Fig. S21: Trait-spot associations for Eosino, Hb, Lym, MCV traits identified by using gsMap embeddings in human lymph datasets as input.

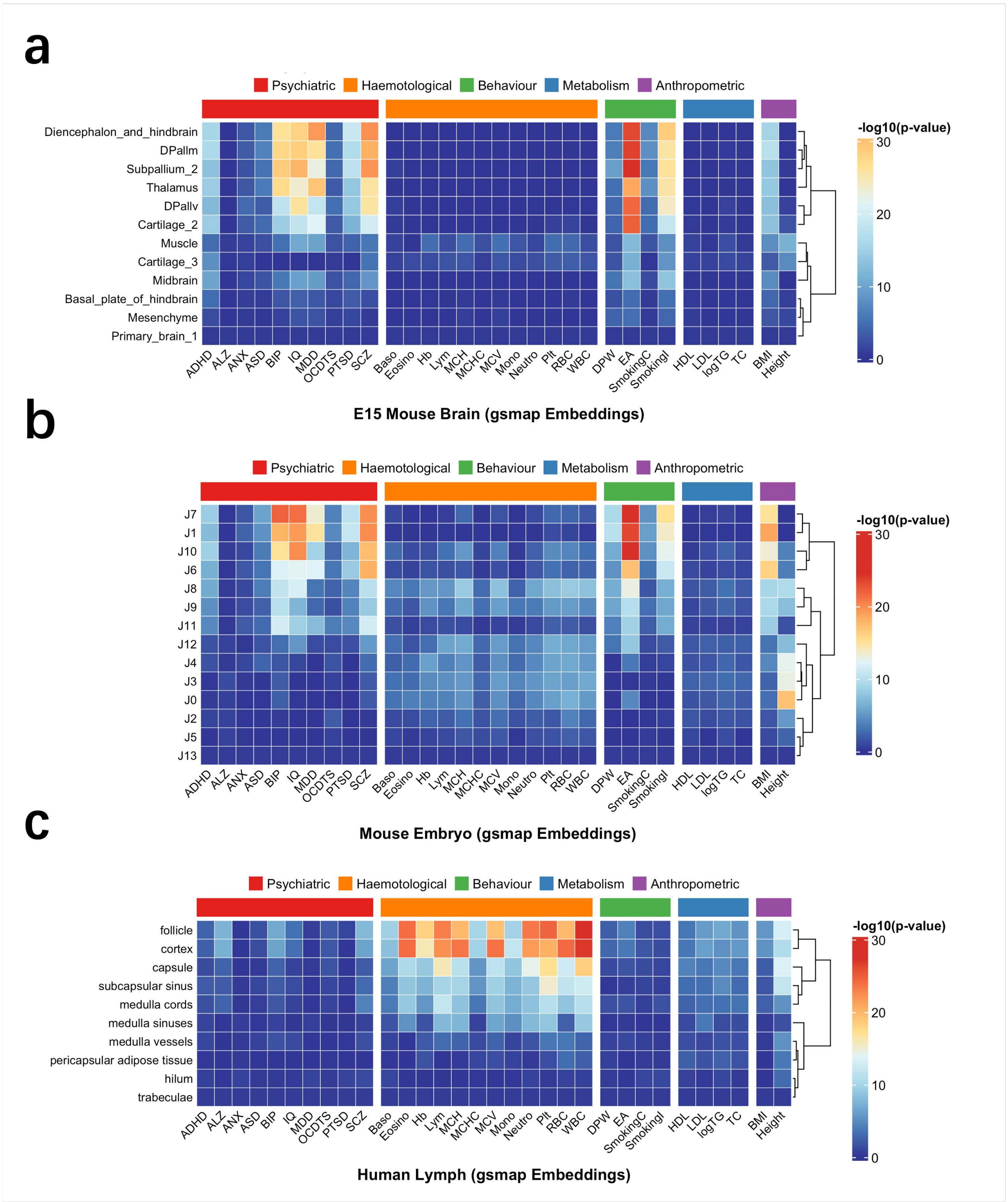

Fig. S22: Heatmaps showing the significance of associations between complex traits and spatial regions by using gsMap embedding.

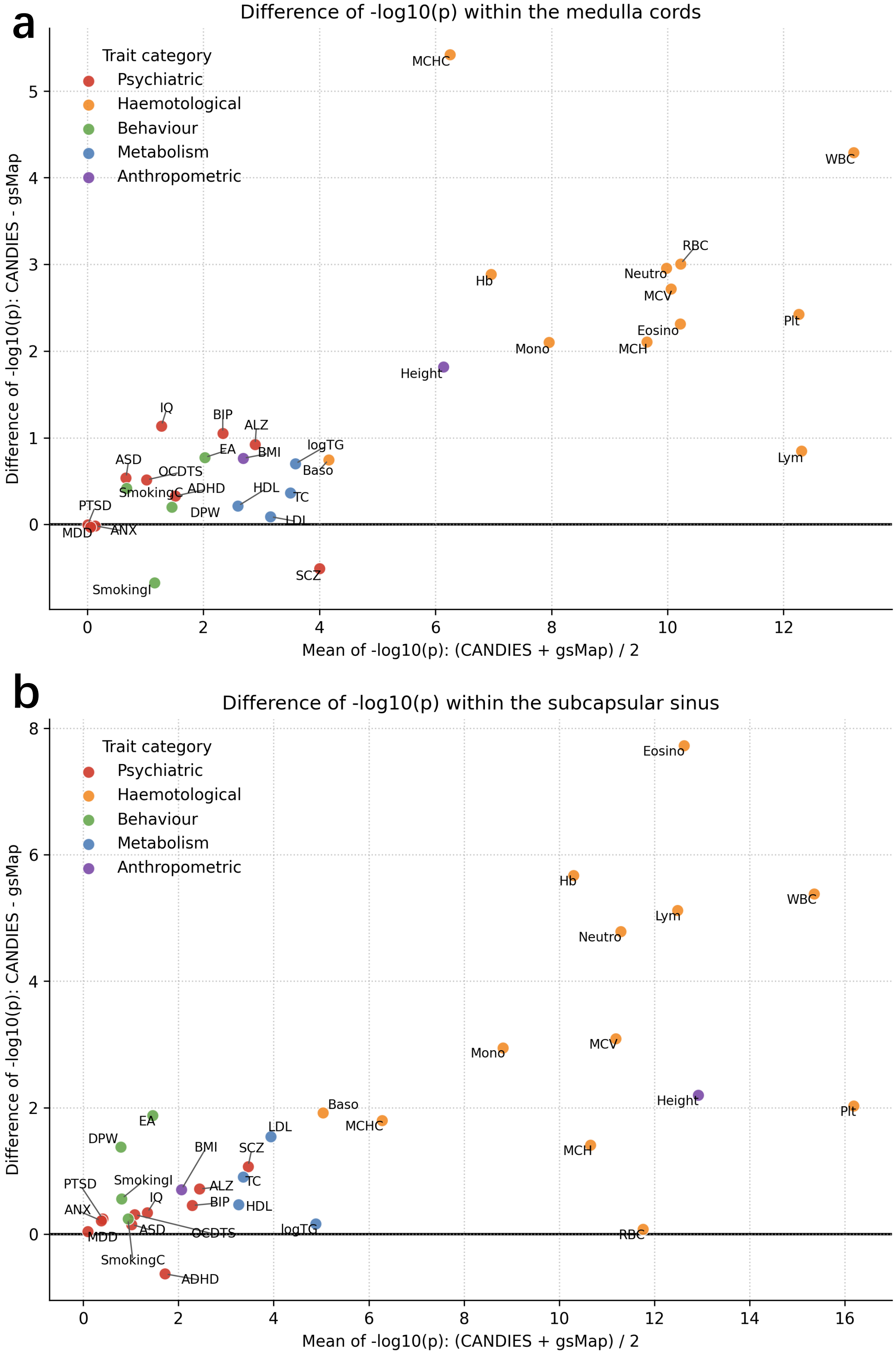

Fig. S23: Comparison of trait-level significance between CANDIES and gsMap across lymph node regions. **a**, Medulla cords. Each point represents one trait; the x-axis shows the mean $-\log_{10}(p)$ across methods (CANDIES + gsMap)/2, and the y-axis shows the difference $-\log_{10}(p)_{\text{CANDIES}} - (-\log_{10}(p)_{\text{gsMap}})$. The horizontal line denotes zero difference. Points are colored by trait category (Psychiatric, Haematological, Behaviour, Metabolism, Anthropometric), as indicated in the legend. **b**, Subcapsular sinus, shown with the same axes.
